# Bonding Trends in Pyridine-2-thiolato Complexes of Tetravalent Actinides

**DOI:** 10.1021/acs.inorgchem.5c03369

**Published:** 2025-10-23

**Authors:** Johannes Balas, Christian Urbank, Peter Kaden, Michael Patzschke, Juliane März, Kristina O. Kvashnina, Moritz Schmidt, Thorsten Stumpf, Robert Gericke

**Affiliations:** † 28414 Helmholtz-Zentrum Dresden—Rossendorf, Institute of Resource Ecology, Bautzner Landstraße 400, 01328 Dresden, Germany; ‡ Institute of Inorganic and Applied Chemistry, University of Hamburg, Martin-Luther-King-Platz 6, 20146 Hamburg, Germany; § The Rossendorf Beamline at ESRF—The European Synchrotron, CS40220, 38043 Grenoble, Cedex 9, France; ∥ Institute of Materials Chemistry, Brandenburg University of Technology Cottbus-Senftenberg, 01968 Senftenberg, Germany

## Abstract

Complexes of tetravalent actinides (An: Th, U, Np, and Pu) with the bidentate (*N,S*)-donor ligand pyridine-2-thiolate (2-PyS, PyS^–^) were synthesized in 1:4 or 1:5 ratios. This includes the first structurally characterized Np complex with (*N,S*)-donor ligands, filling a notable gap in the An coordination chemistry. An improved synthetic approach with PyS–SiMe_3_ enabled efficient formation of the 1:4 complexes in THF as a coordinating solvent. The compounds were comprehensively characterized in solution and in the solid phase, supported by quantum chemical calculations. Experimental and theoretical results show matching trends in the binding behavior of An^IV^. The covalent bond contributions in An–N and An–S bonding increase along the series of An from Th to Pu. The bonds to the soft sulfur donors consistently have the highest covalent contributions with a remarkably high percentage for Pu–S (IQA analysis: >34%) over the harder N donors or An–O bonds of coordinating THF. High-resolution X-ray absorption and infrared spectroscopy indicate similar electronic and structural properties across An^IV^ complexes, while SQUID magnetometry uncovered significant differences in magnetic behavior at low temperatures depending on the complex compositions. This work advances the understanding of An-ligand bonding, emphasizing covalency and electronic structures, and expands fundamental insights into An chemistry.

## Graphical abstract

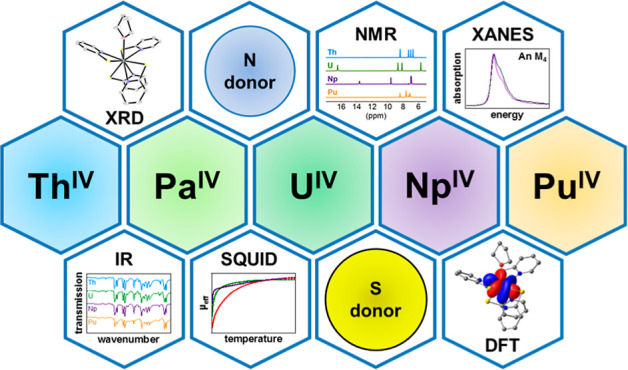

## Introduction

The treatment of spent nuclear fuel is currently one of the major challenges with regard to the sustainable and efficient use of nuclear power to cover the growing global energy demand. The separation of actinides (An) from fission products is of particular interest in order to reduce the level of radioactivity as well as the thermal load during the long-term storage of waste.^1,2^ In the development of tailored ligands for partitioning, those with soft N or S donors have proven to be highly efficient.^1,3–5^ Despite this progress, which can contribute to optimizing the nuclear fuel cycle, numerous fundamental questions regarding the binding behavior of An and its electronic and magnetic properties remain unanswered. For instance, how do the 5f orbitals of An influence chemical bonds, what is the character of the interactions between metal and ligand orbitals, and to what extent does covalency play a role in actinide compounds?^6^ It is often assumed that An bonds with soft donors are more covalent than those involving lanthanides or harder donor atoms,^3,4,6–9^ although with limited quantification. For a deeper understanding of the fundamental properties of An, a detailed analysis of the bonds to different donor atoms, as well as the investigation of trends with experimental and quantum chemical methods, is necessary.

Besides the difficulties in handling radioactive and toxic compounds, especially those containing transuranium (TRU) elements, previous publications on the coordination chemistry of An mainly focused on ligand systems containing hard donor atoms according to Pearson’s hard and soft acids and bases principle, for example, amines and alkoxides.^9,10^ In consequence, ligands with soft sulfur donors are rarely studied for the coordination of An, as indicated by a limited number of structurally characterized compounds. 23 TRU compounds with sulfur donor ligands and only 7 with (*N,S*)-donor ligands have been analyzed using single-crystal X-ray diffraction, where an example for Np was missing entirely so far.^11^ The growing number of studies and investigated compounds shows that bonds between hard An and soft chalcogen donors are not disfavored,^12^ but using sulfur-containing ligands always poses challenges. For example, An complex syntheses with (*N,S*)-donor ligands in coordinating solvents have already been described as infeasible, as the soft S donor of the ligand could not displace the hard O donor of the reaction medium.^13^ In addition, redox-active (*N,S*)-donor ligands such as pyridine-2-thiolate (PyS) can lead to a reduction of the metal centers,^1^ preventing the comparison of isoelectronic An complexes.

However, by choosing suitable ligands and effective synthetic routes, An complexes with soft (*N,S*)-donors can be synthesized. Supporting ligands to improve complex stability, i.e., cyclopentadienyl (Cp) and its derivatives, are commonly used.^11^ Another approach is provided by multidentate ligands, where the chelate effect is thought to contribute significantly to ligand-actinide bonding.^14^ The ambidentate PyS ligand, which was previously used for the coordination of group 10 and group 14 elements as a monoanion,^15,16^ as well as in protonated form for a wide range of lanthanides,^17,18^ is also known in An coordination chemistry. An excellent example for the complexation of a tetravalent An was provided by Neu *et al.*, who oxidized metallic uranium with dipyridyl disulfide to form [U(PyS)_4_(THF)].^19^ Based on these results, we present the synthesis in THF and characterization of tetravalent An complexes with PyS ligands. Targeting a comprehensive bond analysis, complexes of the type [An­(PyS)_4_(THF)_n_] (An: Th, U, Np {*n* = 1}, and Pu {*n* = 0}) were investigated in solution and in the solid phase in combination with quantum chemical calculations. We aim to investigate not only the coordination behavior of An with S, N, and O donors and their corresponding binding strengths but also to quantitatively assess the contributions of covalent bonding. Furthermore, synthetic access to homoleptic complexes of the type K­[An­(PyS)_5_] (An: Th and U) enables a comparison to their [An­(PyS)_4_(THF)_n_] homologues, also with regard to magnetic properties, which are so far underrepresented in literature.^20^

Building on these insights and addressing the challenges inherent to the coordination of actinides with soft donor ligands, this study aims to expand our understanding of An-ligand bonding. The detailed exploration of bonding interactions, covalency, and electronic and magnetic properties deepens our understanding of fundamental aspects of An chemistry.

## Results and Discussion

### Complexes of the Type [An­(PyS)_4_(THF)_n_]

The complexation of An^IV^ was performed via salt metathesis reactions (Scheme 1, left). After the addition of 1 eq. [AnCl_4_(DME)_x_] (An: Th, Np, Pu {*x* = 2}, and U {*x* = 0}) in THF to 4 eq. of the potassium salt of deprotonated 2-mercaptopyridine (KPyS), color changes of the reaction mixtures were observed (details see Supporting Information). After stirring for approximately 1 day at ambient temperature, off-white KCl was centrifuged off. Complexes of the composition [An(PyS)_4_(THF)_n_] (An: Th (**1**), U (**2**), Np (**3**) {*n* = 1}, and Pu (**4**) {*n* = 0}) were obtained from THF/*n*-pentane. Thus, the complexation of An with soft (*N,S*)-donors in THF is possible using the PyS ligand, although ether solvents are described as unsuitable in the literature.^13^ An alternative synthetic route for An^IV^ complexes was developed with the reaction of the actinide precursors and 2-(trimethylsilylmercapto)­pyridine (PyS–SiMe_3_), which was previously used successfully for the synthesis of Si and Ge complexes, but rarely for An compounds.^15,21,22^ The reaction of 4.4 eq. PyS–SiMe_3_ and 1 eq. of [AnCl_4_(DME)_x_] (An: Th, Np {*x* = 2}, and U {*x* = 0}) in THF leads to the formation of **1–3** (Scheme 1, right) in excellent purity. Significant advantages of the PyS–SiMe_3_ synthetic route over KPyS are that the ligand can be used in excess, removing possible traces of moisture and the formation of volatile byproducts. However, for compound **4**, only an insoluble green species could be isolated by this method. Using HERFD-XANES analysis, this species could be identified as a reduced Pu^III^ byproduct (*vide infra*).

**Scheme 1:**
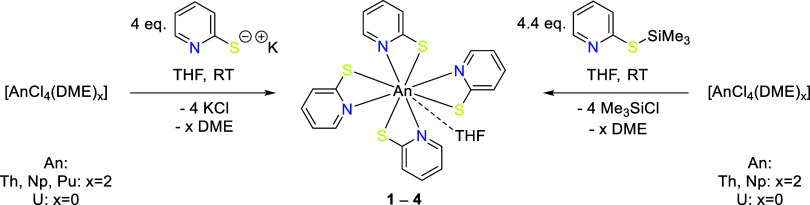
Synthesis Routes of the An Complexes [Th­(PyS)_4_(THF)] **1**, [U­(PyS)_4_(THF)] **2**, [Np­(PyS)_4_(THF)] **3**, and [Pu­(PyS)_4_] **4**, with KPyS (Left) or PyS–SiMe_3_ (Right)

In the ^1^H nuclear magnetic resonance (NMR) spectra of all complexes **1**–**4** in THF-*d*_8_, one set of four signals was observed (Figure 1). This indicates either a fast exchange of binding PyS or high symmetry (*D*_2_ or *D*_2*d*_ due to loss of THF) in solution over time and bulk average. The diamagnetic [Th^IV^(PyS)_4_(THF)] complex **1** shows four temperature-independent signals (Figures S1 and S2) in the aromatic region, which were assigned as depicted in Figure 1 using 2D NMR techniques (see Supporting Information for details). In complexes **2**–**4**, unpaired electrons cause a paramagnetic contribution along the bonds (*Fermi*-contact shift, FCS) and through space (pseudocontact shift, PCS) to NMR chemical shifts.^23^ The ^1^H NMR signals of the aromatic pyridine rings show the strongest influence of paramagnetic shifts for the [U^IV^(PyS)_4_(THF)] complex **2** (δ = 5.67, 8.09, 8.62, and 16.47 ppm in THF-*d*_8_). In contrast, the three ^1^H NMR signals in a 4:8:4 ratio of the Pu^IV^ complex **4** (δ = 7.05, 7.54, and 8.33 ppm in THF-*d*_8_) show only a weak paramagnetic influence. For both complex **2** and **3**, in variable temperature 1D ^1^H NMR spectra, a pronounced low-field shift of H-6 is observed upon cooling (2–4 ppm), whereas for the other signals only a minor change (∼1 ppm) is visible (Figures S12 and S17). Complex **4** shows only minor changes (<1 ppm) for all PyS protons in the ^1^H NMR spectra during cooling (Figure S23).

**Figure 1:**
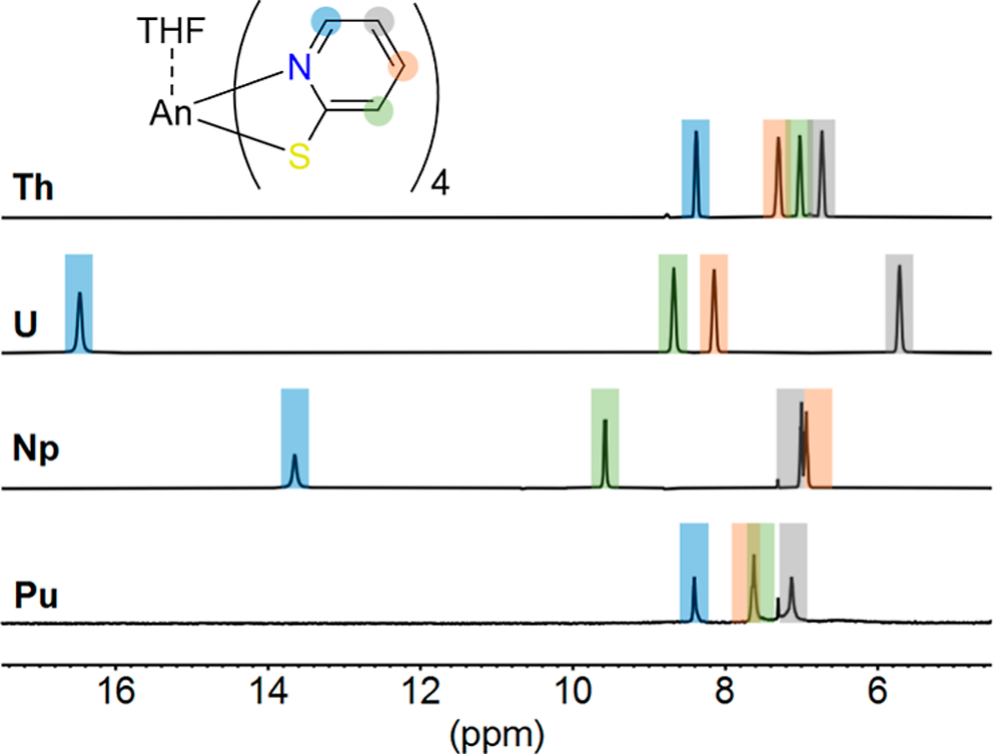
^1^H NMR spectra of **1**–**4** at room temperature in THF-*d*_8_. Assignment of the ligand proton signals with 2D NMR spectroscopy: H-3 (green), H-4 (red), H-5 (gray), and H-6 (blue).

Shoulders on the THF signals (Figure S36) suggest that solvent molecules contribute to the saturation of the coordination spheres by coordinating to the An centers (An: Th, U, or Np). Interestingly, no THF signals can be found in the ^1^H and ^13^C NMR spectra of a vacuum-dried solid of **4** in DCM-*d*_2_. This indicates a possible formation of [Pu­(PyS)_4_] over [Pu­(PyS)_4_(THF)] during crystallization, likely due to steric hindrance at the small Pu^IV^ ion.^24^ Triggered by this finding, attempts were made to synthesize the An complexes as solvent-free compounds [An­(PyS)_4_] (An: Th, U, Np, and Pu) by reacting KPyS with [AnCl_4_(DME)_x_] (An: Th, Np, Pu {*x* = 1}, and U {*x* = 0}) in noncoordinating dichloromethane. However, in the corresponding ^1^H NMR spectra in DCM-*d*_2_ another set of signals was found (δ = 4.90, 6.98, 7.10, 7.45, and 8.40 ppm) next to very broad complex signals and could be assigned via 2D NMR spectroscopy to bis­(2-pyridylthio)­methane (Figure S37). This species likely forms as a direct consequence of DCM activation, driven by the influence of An.^25^ Baker *et al.* have previously reported a similar C–Cl bond activation at DCM, which was mediated by uranyl chloride and KOH.^26^ The bis­(2-pyridylthio)­methane could be reproducibly identified for all An complex syntheses in DCM with NMR and single-crystal X-ray diffraction (Figure S38).^27^ Dissolving KPyS in DCM does not result in the formation of bis­(2-pyridylthio)­methane, which clearly demonstrates that C–Cl bond activation is possible with actinide mediation, even though the exact mechanism remains unclear.

Infrared spectroscopy (IR) shows that the [An(PyS)_4_(THF)] complexes **1**–**3** as bulk material are isostructural, as highly similar vibrational band positions and intensities were found (Figure S41). Minor shifts to higher wavenumbers of characteristic band positions can be observed along the An^IV^ series (e.g., ν­(C=S) for **1**: 1132 cm^–1^, **2**: 1133 cm^–1^, and **3**: 1134 cm^–1^).^28^ For **4**, the bands of a coordinating THF molecule (δ­(C–H): 820 cm^–1^ and 840 cm^–1^)^28,29^ are missing in the IR spectrum of the vacuum-dried solid, although the complex was synthesized in THF. This confirms the formation of a solvent-free solid-state structure [Pu­(PyS)_4_]. Furthermore, similar vibrational band positions and intensities were found for **4** in comparison to the IR spectra of **1**–**3**, indicating similar binding properties for [An­(PyS)_4_(THF)_n_] (An: Th, U, Np {*n* = 1}, and Pu {*n* = 0}).

Crystals suitable for single-crystal X-ray diffraction analysis (SC-XRD) of **1**–**3** were obtained by slow evaporation of THF/*n*-pentane complex solutions. The molecular structures exhibit *C*_1_ symmetry. During crystallization, dynamic equilibration of different possible isomers is thus prevented, and the symmetry of the complexes is reduced. Nevertheless, the high dynamics of the complexes in solution can be illustrated by various isomers that have been identified by SC-XRD (Figure 2), and thus different molecular geometries A, B, and C are accessible for complexes of the type [An­(PyS)_4_(THF)].

**Figure 2:**
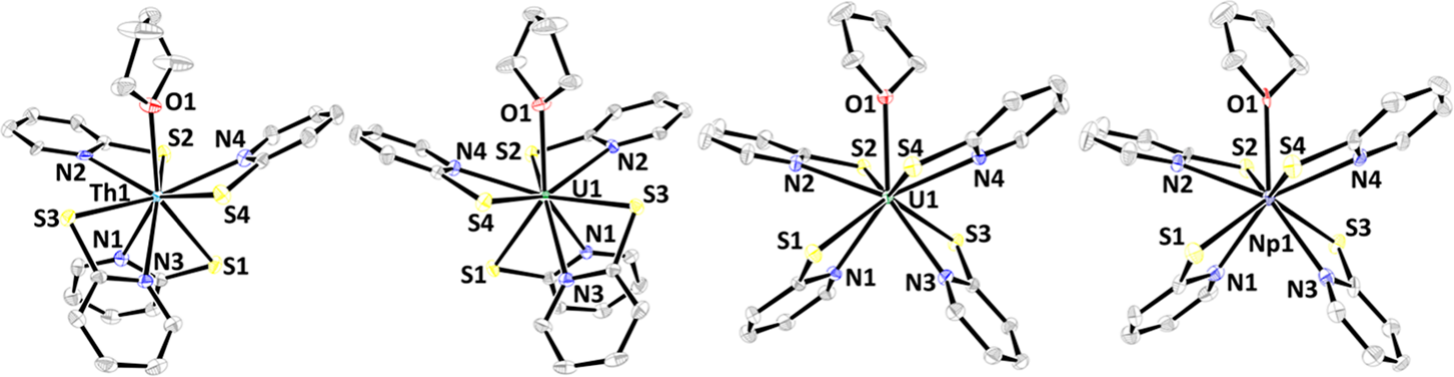
Molecular structures of [Th­(PyS)_4_(THF)] **1** ^**A**^ (left), [U­(PyS)_4_(THF)] **2** ^**B**^ (middle left) and **2** ^**C**^ (middle right), and [Np­(PyS)_4_(THF)] **3** ^**C**^ (right). Ellipsoids are shown at a 50% probability level. Hydrogen atoms are omitted for clarity.

[Th­(PyS)_4_(THF)] (**1** ^**A**^) crystallizes in the chiral orthorhombic space group *P*2_1_2_1_2_1_ and is isostructural to the previously reported molecular structure of [U­(PyS)_4_(THF)] (**2** ^**A**^) by Neu *et al.* (isomer A).^19^ From the crystal batch of [U­(PyS)_4_(THF)], the mirrored enantiomer to **2** ^**A**^ could be analyzed (**2** ^**B**^, isomer B, Figure 2, middle left). In both compounds, the molecular structures exhibit a 9-fold coordination sphere around the actinide, with four PyS ligands coordinating in a chelating motif and one THF molecule. The coordination sphere can be best described as either a distorted tricapped trigonal prism or a distorted capped square antiprism using continuous shape measures.^30^

Furthermore, [U­(PyS)_4_(THF)] (**2** ^**C**^) and [Np(PyS)_4_(THF)] (**3** ^**C**^) were found to crystallize in the triclinic space group *P*1̅, where **3** ^**C**^ is, to the best of our knowledge, the first molecular structure of a Np complex with (*N,S*)-donor ligands.^11^ Both crystal structures of **2** ^**C**^ and **3** ^**C**^ contain isostructural complex structures (isomer C). The most striking difference between enantiomers A/B and isomer C is the orientation of the Lewis-hard N- and O-donor atoms. In the crystal structures of **2** ^**C**^ and **3** ^**C**^, the actinide, nitrogen, and oxygen donor atoms are all located in a plane with a deviation of <0.210(2) Å and <0.238(8) Å, respectively. The softer sulfur atoms are located above (S1, S4) or below (S2, S3) this plane. This planar orientation of five donor atoms around the actinide is missing in enantiomers A and B. The average U–S and U–N bond distances between **2** ^**B**^ and **2** ^**C**^ are the same within the experimental error. Only the U–O distance is significantly shorter in **2** ^**B**^ by about 0.043(6) Å, indicating a stronger effect of crystal packing on the THF ligand over the PyS.

In solution, only one set of NMR resonances was observed for each complex **1**–**4**. This suggests rather small energy barriers between the different possible isomers. To investigate this in more detail, quantum chemical calculations at the DFT level of theory (Figure S40) have shown that the Gibbs free energy for isomer B is almost identical to isomer C for [An­(PyS)_4_(THF)] (|Δ*G*| = 0.20 (Th), 0.27 (Pa), 0.51 (U), 0.82 (Np), and 0.98 kcal/mol (Pu); Figure S40).

To evaluate the coordination of a THF molecule, a comparison with average bond lengths *d*_An–O_ of 9-fold coordinated An^IV^ complexes known from literature (*d*_Th–O_: 2.46(9), *d*_U–O_: 2.40(16) Å)^11^ shows that An–O is slightly elongated for **1** and **2** by ∼0.1 Å (Table 1). Thus, THF can be considered the most labile ligand within the investigated complexes.

**Table 1:** Selected Average Bond Lengths (*d*_An–X_, Å, Standard Deviation as Error in Parentheses) in the Molecular Structures of **1**–**3** in Comparison with Sum of Covalent Radii (∑(*r*_cov._), Å)^31^

	1^A^ (Th)	2^B^ (U)	2^C^ (U)	3^C^ (Np)
*d*_An–N_	2.586(27)	2.537(28)	2.544(24)	2.528(30)
∑(*r*_cov._; An–N)	2.77(7)	2.68(8)		2.61(2)
*d*_An–S_	2.889(5)	2.828(5)	2.827(17)	2.807(20)
∑(*r*_cov._; An–S)	3.11(8)	3.02(9)		2.95(4)
*d*_An–O_	2.561(4)	2.532(1)	2.489(6)	2.489(5)
∑(*r*_cov._; An–O)	2.72(7)	2.63(8)		2.56(3)

Along the An series, the average An–N and An–S bond lengths decrease, with decreasing ionic radii of the An centers (Table 1).^24^ The An–N bonds are always shorter (∼0.3 Å) than the An–S bonds. Interestingly, An–N, An–S, and even An–O bonds are all shorter than the sum of the corresponding covalent radii, indicating a possible covalent bond contribution.^31^

In order to provide deeper insights into the binding behavior of the PyS ligand toward the An, quantum chemical calculations at the DFT level of theory were conducted. Starting from the molecular structure **2** ^**B**^, the geometries of the series of complexes of the type [An­(PyS)_4_(THF)] (An: Th–Pu) were optimized (Figure S40).

The calculated and experimental structural parameters show a high level of agreement regarding actinide ligand bonding (Tables 1 and 2, Figure 3, left), demonstrating that the applied method is effective for prediction of the hitherto unobserved molecular structures of the Pa^IV^ and Pu^IV^ complexes. The *d*_An–N_ and *d*_An–S_ bond lengths decrease linearly (*R* ^2^_An–N_: 0.99, *R* ^2^_An–S_: 0.96) with the covalent radii of the actinide (Figure 3, left).^31^ This again indicates a covalent contribution to actinide PyS bonding. The experimental An–O_THF_ bond lengths show a shortening along the series from Th to Np with some variation between the different isomers, presumably dominated by different packing effects. Calculated An–O bond lengths exhibit a decrease from Th–U and a slight increase from U–Pu. In effect, the An–O bond lengths remain essentially constant from Pa to Pu. The smaller covalent radius of Pu^IV^ over Th^IV^, U^IV^, and Np^IV^ leads to the shortest An–N and An–S bonds in the series, i.e., the PyS ligands are closest to Pu^IV^. Steric crowding between PyS and THF likely makes the coordination of an additional THF to Pu unfavorable. This underpins the assumption that the coordinative bond to THF is rather labile and that the coordination of a solvent molecule for the smaller An^IV^ is increasingly hindered. Similar weak solvent coordination on actinide complexes has recently been investigated to comparable results.^32^

**Table 2:** Results of Quantum Chemical Calculations^a^

An	Th	Pa	U	Np	Pu
*d*_An–N_ (Å)	2.59(3)	2.57(3)	2.54(2)	2.52(3)	2.51(4)
*d*_An–S_ (Å)	2.89(1)	2.85(1)	2.83(1)	2.81(2)	2.80(2)
*d*_An–O_ (Å)	2.56	2.52	2.51	2.52	2.52
|*V*(*r*_b_)|/*G*(*r*_b_)_An–N_	1.210	1.194	1.182	1.177	1.166
|*V*(*r*_b_)|/*G*(*r*_b_)_An–S_	1.365	1.368	1.363	1.343	1.325
|*V*(*r*_b_)|/*G*(*r*_b_)_An–O_	1.085	1.083	1.068	1.056	1.038
DI_An–N_	0.31(1)	0.33(1)	0.34(2)	0.34(2)	0.35(3)
DI_An–S_	0.42(1)	0.46(1)	0.48(2)	0.49(3)	0.51(2)
DI_An–O_	0.26	0.27	0.28	0.26	0.25
natural charge An (*e*)	0.86	0.68	0.57	0.53	0.51
natural charge N (*e*)	–0.51(1)	–0.49(1)	–0.49(1)	–0.48(1)	–0.48(1)
natural charge S (*e*)	–0.16(1)	–0.13(2)	–0.11(2)	–0.11(2)	–0.10(2)
natural charge O (*e*)	–0.50	–0.49	–0.47	–0.48	–0.48
cov_An–N_ (%)	8.1(1)	8.9(1)	9.4(1)	9.8(1)	10.2(1)
cov_An–S_ (%)	24.6(5)	27.5(11)	29.7(12)	31.4(11)	33.5(14)
cov_An–O_ (%)	8.0	8.7	9.1	8.9	8.9

a Average bond lengths (*d*_An–X_, Å, standard deviation as error in brackets) in the geometry optimized structures (isomer B). Ratio |*V*(***r***_b_)|/*G*(***r***_b_) (where *V*(***r***_b_): potential energy density and *G*(***r***_b_): Lagrangian kinetic energy) and delocalization indices (DI) obtained from QTAIM analysis. Natural charges obtained from natural population analysis (NPA). Percentage covalent bond contributions obtained from interacting quantum atoms (IQA) analysis.

**Figure 3:**
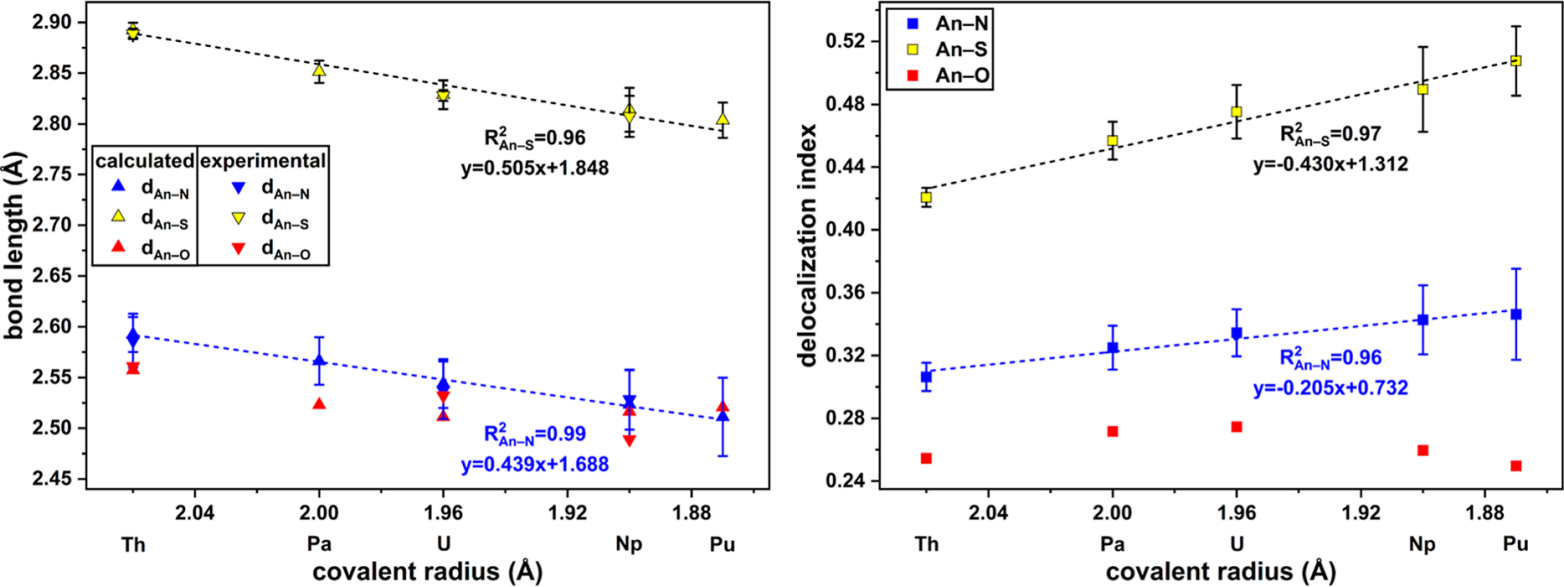
Bond lengths in Å (left) and delocalization indices (right) of the coordinative bonds in [An­(PyS)_4_(THF)] with linear regressions for An–N and An–S (standard deviations as error bars), plotted against the covalent radii of the early An.^31^

To further characterize the actinide ligand bonding in [An­(PyS)_4_(THF)], calculations based on Bader’s quantum theory of atoms in molecules (QTAIM) were conducted.^33^ On the condition that a bond critical point ***r***_b_ exists, the ratio |*V*(***r***_b_)|/*G*(***r***_b_) (where *V*(***r***_b_): potential energy density and *G*(***r***_b_): Lagrangian kinetic energy) can be used to describe local topological properties and evaluate the bonding situation.^34^ Predominantly ionic bonds typically show values |*V*(***r***_b_)|/*G*(***r***_b_) < 1, whereas values |*V*(***r***_b_)|/*G*(***r***_b_) > 2 indicate primarily covalent bond contributions, while values between 1 and 2 indicate bonds with an intermediate character.^35^ The An–S, An–N, and An–O bonds in [An­(PyS)_4_(THF)] show values between 1.04 (Pu–O_THF_) and 1.37 (Th/Pa–S), and thus exhibit ionic and covalent bonding contributions (Table 2), with covalent contributions consistently following the order An–O < An–*N* < An–S. In addition, the |*V*(*r*_b_)|/*G*(*r*_b_) ratios suggest a slight increase in ionic character along the An series.

However, it needs to be critically discussed whether local topological properties are sufficient to characterize bonds between An and donor atoms. The analysis of integral topological indices is considerably more useful, with the delocalization index (DI) being used most frequently.^34,36–38^ It is directly correlated with the number of shared electrons and can be used to evaluate the covalent contributions in coordinative bonds.^34^ A DI value of 0 corresponds to purely ionic interactions, while a DI of 1 indicates a bond equivalent to a C–C single bond. Plotting the DIs against the covalent radii of the actinides in [An­(PyS)_4_(THF)], a linear increase (*R* ^2^_An–N_: 0.96, *R* ^2^_An–S_: 0.97) for the An–N and An–S bonds was observed (Figure 3, right).^31^ This suggests an increasing covalent character in the coordinative bonds along the series of early actinides. The DI of An–S are constantly the highest (e.g., for **2**: DI_U–S_: 0.48, DI_U–N_: 0.34, and DI_U–O_: 0.28) and exhibits the strongest increase along the An series. Thus, it can be concluded that the bond to the soft sulfur donor has the strongest covalent character of the coordinative bonds in the investigated complexes, especially for An with 5f electrons. The THF appears to bind rather labile, which is in agreement with the low corresponding DI_An–O_, which also decreases from U towards Pu.

The natural charges of An^IV^, obtained from natural population analysis (NPA), decrease along the series of early An atoms in [An­(PyS)_4_(THF)] (Table 2 and Figure 4). This points to an improved transfer of electron density from PyS to the An^IV^ from Th to Pu and is also reflected by slightly increasing charges of the donor atoms.

**Figure 4:**
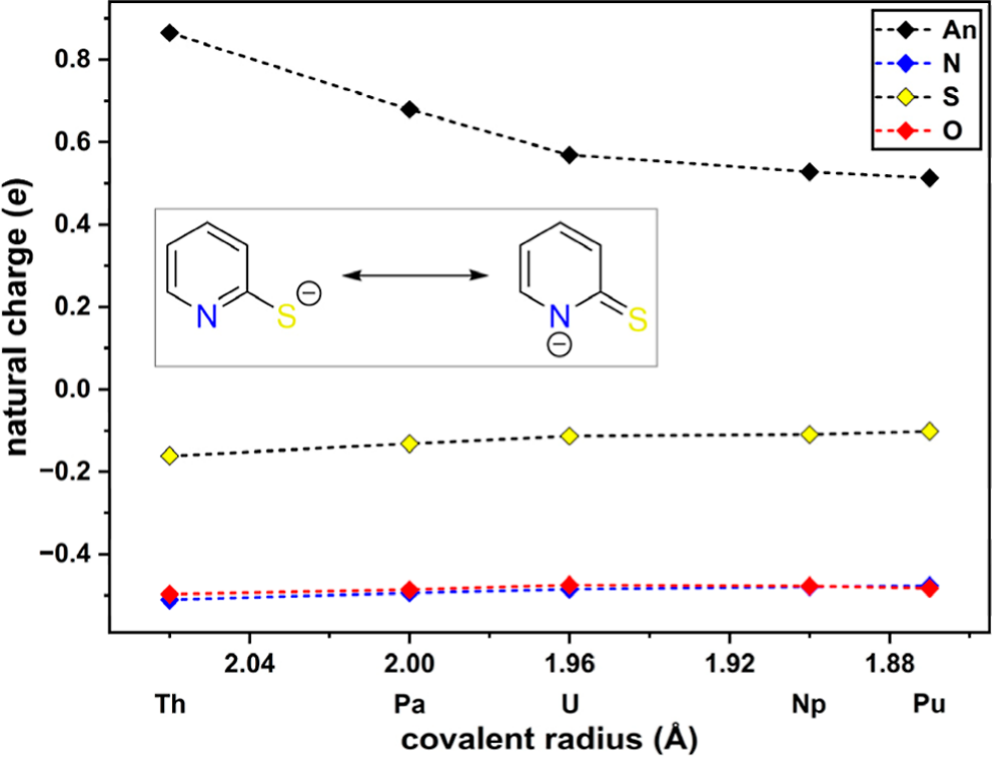
Mesomeric structures of PyS^–^. Natural charges of An^IV^ and donor atoms in [An­(PyS)_4_(THF)], plotted against the covalent radii of the early An.^31^

Interestingly, the negative natural charges of the nitrogen- and oxygen*-*donor atoms exhibit approximately the same values. However, the natural charges of the sulfur donors are lower in absolute value. The PyS ligand could be preferentially presented as its thioketone structure rather than as the thiolate structure in [An­(PyS)_4_(THF)] (Figure 4). This should be reflected in the C–S bond lengths. As reference, the crystal structure of sulfur bound PyS–SiMe_3_ has previously been reported with a C–S single bond of 1.775(2) Å, whereas the nitrogen bound PyS–SiMe_3_ exhibits a calculated C=S double bond of 1.68 Å.^39^ On average, the C–S bond lengths in the [An­(PyS)_4_(THF)] complexes are 1.745(9) Å and are therefore in between the thioketone and thiolate structure, which is supported by an average DI_C–S_ of 1.27. Noncoordinating PyS^–^ exhibit a fairly equal charge distribution between the N and S atom (NC_N_ = −0.52 e, NC_S_ = −0.58 e, *d*_C–S_ = 1.73 Å). Noncoordinating THF shows the same natural charge (NC_O_ = −0.52 e) on O as observed for N in PyS^–^. It appears that the sulfur donor exhibits a higher tendency over the N- and O-donor atoms to shift electron density toward the An centers during coordination due to a more efficient orbital overlap and, in consequence, a more pronounced covalent character of the An–S over the An–N and An–O bonds. This is in good agreement with the results of the QTAIM analysis and the experimental observations.

In order to quantify the covalent and ionic bonding contributions in the coordinative bonds of the [An(PyS)_4_(THF)] complexes, calculations using the interacting quantum atom (IQA) method were carried out. This allows the evaluation of the binding energies and the percent share of bonding contributions by using the orbital invariant partitioning of the total molecule energy sum, consisting of the self-energies of the involved atoms and their respective interaction energies.^40^ The individual energy contributions (Table S19) as well as the percentage covalent bond contributions (Figure 5) confirm the most covalent character in the An–S bond (25–34%), being approximately thrice as much as for An–N or An–O (approximately 9%, respectively) for all actinides of interest. Notably, the covalent character of An–S is linearly increasing along the An series, in agreement with the results of the QTAIM analysis. The covalent bond contributions of the N and O donors, on the other hand, remain essentially constant.

**Figure 5:**
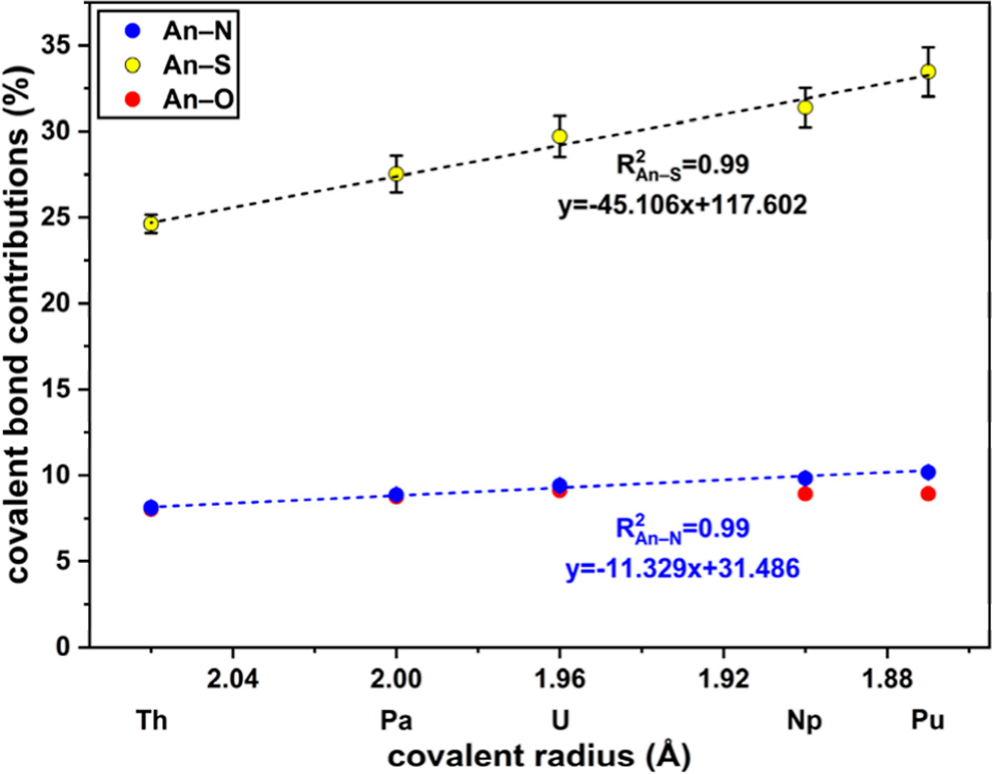
Percentage covalent bond contributions of the coordinative bonds in [An­(PyS)_4_(THF)], plotted against the covalent radii of the early An.^31^

Thus, IQA analysis adds quantitative support to the prior conclusion that the bonds of An^IV^ to the soft sulfur donors show the strongest covalent bond contributions (Th–S: 24.6(5)%_COV_, Pu–S: 33.5(14)%_COV_), with remarkably high covalency of the Pu–S bonds. Even though the total energies of the coordinative bonds are mainly determined by the ionic contributions (Table S19), the (partial) covalent An–S bond energies increase along the early An series and contribute to the stability of the bonds, especially for Pu–S. The An–N and An–O bonds have similar low covalent bond contributions (Th–N: 8.1(1)%_COV_, Pu–N: 10.2(1)%_COV_, Th–O: 8.0%_COV_, Pu–O: 8.9%_COV_) and are therefore more ionic in character. The (partial) ionic bond energies decrease along the An series (Table S19), highlighting the unique role of the soft S donor. While the harder N and O donors can form stronger ionic bonds with An, particularly for Th–U, it is the covalent contributions of sulfur that exert the most significant influence on coordinative bonding. The qualitative analysis of bond covalency explains the notably high selectivity of S-containing ligands in the separation of TRU elements, as reported in the literature.^1–3,5,6,41^

### Complexes of the Type K­[An­(PyS)_5_]

Analogous to [An­(PyS)_4_(THF)_n_], salt metathesis reactions with KPyS were used for the syntheses of K­[An­(PyS)_5_] (Scheme 2). After the addition of 1 eq. of [AnCl_4_(DME)_*x*_] (An: Th {*x* = 2} and U {*x* = 0}) in THF to 5 eq. KPyS, color changes of the reaction mixtures were observed in comparison to the precursors, indicating complex formation, where the solutions appeared more intensely colored than for the 1:4 complexes.

**Scheme 2:**
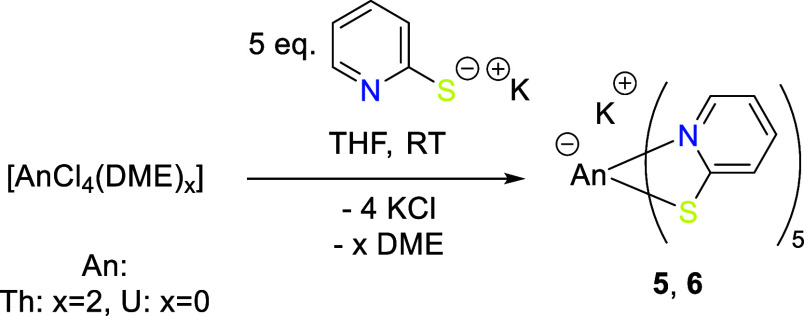
Synthesis of the An Complexes K­[Th­(PyS)_5_] **5** and K­[U­(PyS)_5_] **6** via Salt Metathesis Reactions with KPyS

In analogy to [An­(PyS)_4_(THF)_n_], the ^1^H NMR spectra of isolated K­[An­(PyS)_5_] (An: Th (**5**) and U (**6**)) in THF-*d*_8_ show a single set of four signals with the same integral intensities, indicating dynamic equilibration (Figure 6). Compared to **1**, the ^1^H NMR signals in **5** (δ = 6.46, 6.72, 7.02, and 8.39 ppm in THF-*d*_8_) are in the same order and only shifted by a maximum of 0.30 ppm. This could indicate similar binding properties of the 1:4 and 1:5 complexes.

**Figure 6:**
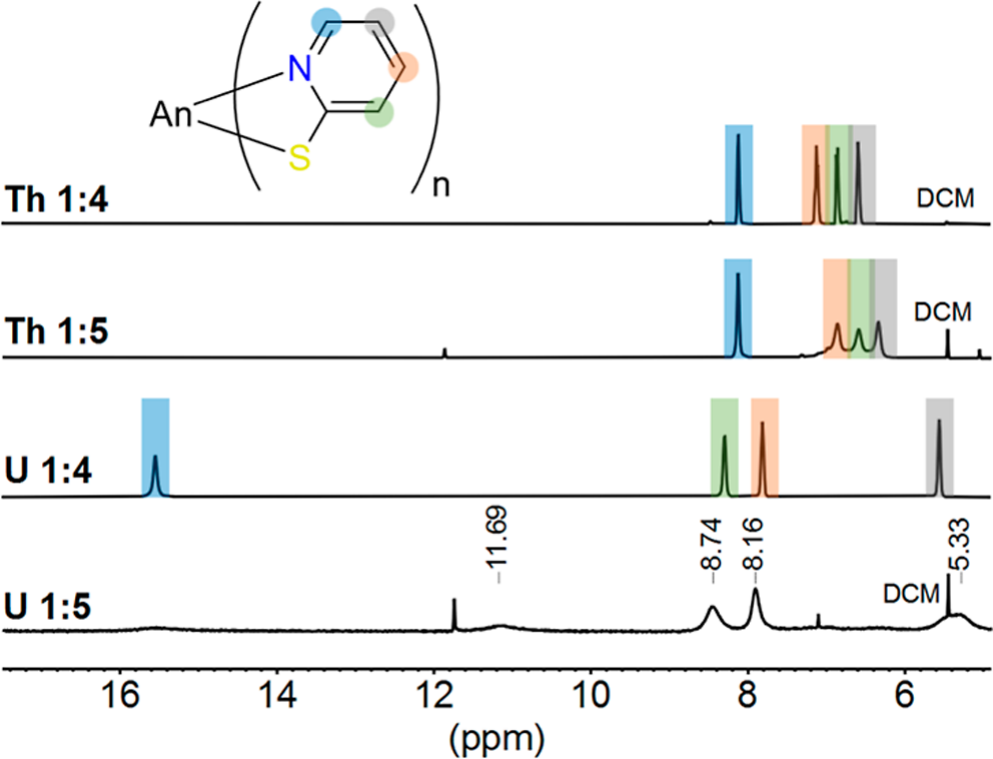
^1^H NMR spectra of [An­(PyS)_4_(THF)] (Th: **1** and U: **2**) and K­[An­(PyS)_5_] (Th: **5** and U: **6**) at room temperature in THF-*d*_8_. Assignment of the ligand proton signals with 2D NMR spectroscopy: H-3 (green), H-4 (red), H-5 (gray), and H-6 (blue).

For compound **6**, broad ^1^H NMR signals are found (δ = 5.33, 8.16, 8.74, and 11.69 ppm in THF-*d*_8_), hindering an assignment with 2D NMR spectroscopy. The influence of paramagnetic shifts is slightly changed compared to that of **2**. Thus, for K­[U­(PyS)_5_], a different orientation of the PyS ligands around the uranium compared to that of [U(PyS)_4_(THF)] is expected and reflected in the spectra. However, the broad ^1^H NMR signals of K­[U­(PyS)_5_] in comparison to [U­(PyS)_4_(THF)] point toward a slower dynamic in solution. Furthermore, small amounts of **2** are found in the ^1^H NMR spectrum, measured in THF-*d*_8_. Due to the strong excess of solvent molecules, a conversion to [U­(PyS)_4_(THF)] may occur, with the hard oxygen donor atom coordinating to U^IV^. This could be confirmed by crystallization of **6** from THF, where single crystals of **2** ^**C**^ were obtained and measured with single-crystal X-ray diffraction (Figure 2).

For the syntheses of a putative K­[Np­(PyS)_5_] complex, reactions with 1 eq. [Np­(PyS)_4_(THF)] and 1 eq. KPyS were attempted. The ^1^H NMR spectrum in THF-*d*_8_ at 298 K shows five broad signals (Figure S30). By lowering the temperature to 218 K, sharp ^1^H (δ = 6.64, 7.27, 7.75, and 13.14 ppm; ratio: 1:2:1:1, Figure S31) and ^13^C­{^1^H} (δ = 112.43, 134.25, 136.78, 139.44, and 180.83 ppm, Figure S32) spectra were measured, showing signals differing from those of [Np­(PyS)_4_(THF)] **3**. The presence of one noncoupling signal in each of the ^1^H,^1^H-COSY (^1^H: 13.14 ppm, Figure S33) and ^1^H,^13^C-HSQC (^13^C: 180.83 ppm, Figure S34) spectra suggests that the protonated ligand may be involved in the complex species formed. The Np^IV^ complexes appear to be particularly sensitive to hydrolysis or protonation. In an attempt to crystallize K­[Np­(PyS)_5_] from THF/*n*-pentane, crystals suitable for single-crystal X-ray diffraction analysis were obtained and exhibit the molecular structure of [Np­(PyS)_4_(PySH)] (Figure S39). K­[Np­(PyS)_5_] appears to be synthetically inaccessible under the chosen conditions.

By diffusion of diethyl ether into a DCM solution of **5**, crystals suitable for SC-XRD were obtained. The corresponding molecular structure shows a 10-fold coordination of Th^IV^ by five PyS^–^ ligands (Figure 7). Furthermore, potassium cations for charge compensation and strongly disordered DCM solvent molecules are part of this solid-state structure.

**Figure 7:**
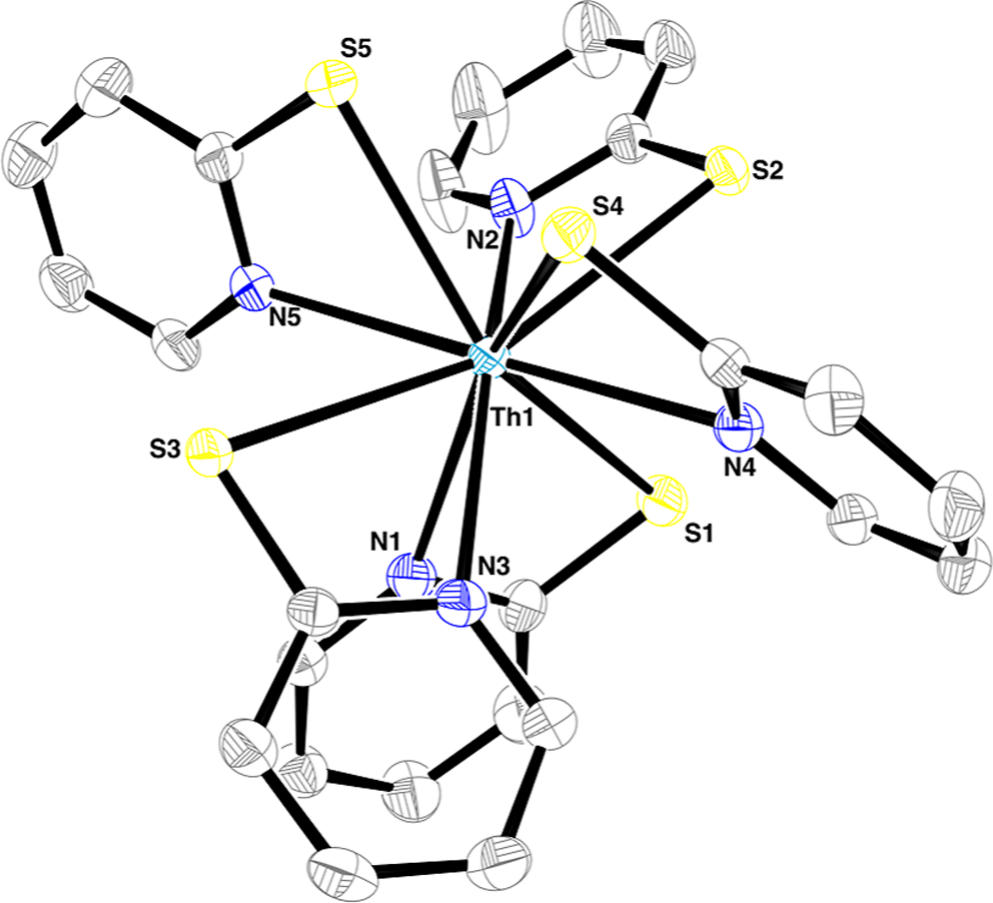
Molecular structure of K­[Th­(PyS)_5_]·2.075 (DCM) **5**. Ellipsoids are shown at a 50% probability level. Hydrogen atoms, potassium cations, and noncoordinating solvent molecules are omitted for clarity.

Compared to [Th­(PyS)_4_(THF)], longer Th–N and Th–S bond lengths can be found in **5** (Th–N: 2.67(2) Å, Th–S: 2.94(3) Å) by about ∼0.05(2) and ∼0.08(4) Å, respectively. This elongation can originate from the higher coordination number at Th in **5** over that in **1**, a change in the crystal packing of the molecular structure, and possible electronic effects.

Using IR spectroscopy, similar band positions and intensities are found for [An­(PyS)_4_(THF)] and K­[An­(PyS)_5_] (An: Th and U) (Figure S43). Nevertheless, differences can be identified in the fingerprint region that allow a distinction between 1:4 and 1:5 An complexes. Characteristics for **1** and **2** are the δ­(C–H) vibrations of the coordinating THF molecule (820 cm^–1^ (m) and 840 cm^–1^ (m)), as well as further vibrational bands at 1095 cm^–1^ (m) and 1473 cm^–1^ (vw).^28,29^ Unique for **5** and **6** are the vibrational bands at 897 cm^–1^ (m), 1053 cm^–1^ (m), 1491 cm^–1^ (w), and in particular at 1615 cm^–1^ (m). Furthermore, the wavenumbers of the bands associated with the (*N,S*)-donor atoms decrease slightly with increasing coordination number (CN) of the An (e.g., ν­(C=S): **1**/**5**: 1586/1584 cm^–1^, **2**/**6**: 1585/1582 cm^–1^; ν­(C=S): **1**/**5**: 1001/1000 cm^–1^, **2**/**6**: 1002/998 cm^–1^).^28,29^ This may be interpreted as a weaker bond strength in **5** and **6**, as with increasing CN, the electron density is shifted from any single donor atom to the An. The associated increase in bond lengths *d*_Th–N_ and *d*_Th–S_ has been observed with SC-XRD for **5** compared to **1**. However, the differences among all four Th^IV^ and U^IV^ complexes are small.

### HERFD-XANES of Complexes of the Type [An(PyS)_4_(THF)_n_] and K­[An­(PyS)_5_]

By X-ray absorption spectroscopy, the chemical state of the metal ions in [An(PyS)_4_(THF)_n_] (An: Th, U, Np {*n* = 1}, and Pu {*n* = 0}) and K­[An­(PyS)_5_] (An: Th and U) can be characterized by the corresponding X-ray absorption near-edge structure (XANES) spectra. Using U as an example, the main absorption edge in the corresponding U M_4_ XANES spectrum occurs via the electronic transition U 3*d*_3/2_ to 5*f*_5/2_.^42^ With conventional methods, broad absorption spectra are thus obtained due to the large core-hole lifetime broadening (approximately 4 eV for An M_4_ edges).^43^ Using the X-ray emission setup^44^ and HERFD (high energy-resolution fluorescence-detection) mode, high-energy resolution An M_4_ XANES spectra can be recorded, where the resolution is substantially improved due to the lower 4*f*_5/2_ core-hole lifetime broadening, in the example of uranium.^42,45^ The analysis of HERFD-XANES spectra enables the determination of important influencing variables for all An with unprecedented sensitivity, such as the oxidation states of the metal centers (detectable Δ*E*: 0.5–1 eV) and the ligand field splitting of the 5*f* shell (detectable Δ*E*: ∼0.1 eV).^42^ Nevertheless, such measurements of molecular actinide complexes remain scarce in the literature. By comparison with An^IV^O_2_ (An = Th–Pu) as reference substances, it is possible to confirm the tetravalent oxidation state of An in [An­(PyS)_4_(THF)_n_] **1**–**4** based on the absorption maxima occurring in the HERFD-XANES spectra (Figure 8). For **1**, the absorption maximum is at the same energy as that of the Th^IV^O_2_ reference. With the presence of 5*f* electrons in **2**–**4**, the absorption maximum is slightly shifted to lower energies ({M_4_} Δ*E*_max,U_: −0.1 eV, Δ*E*_max,Np_: −0.2 eV, and Δ*E*_max,Pu_: −0.2 eV), whereas these differences are in the range of the resolution of the measuring method.

**Figure 8:**
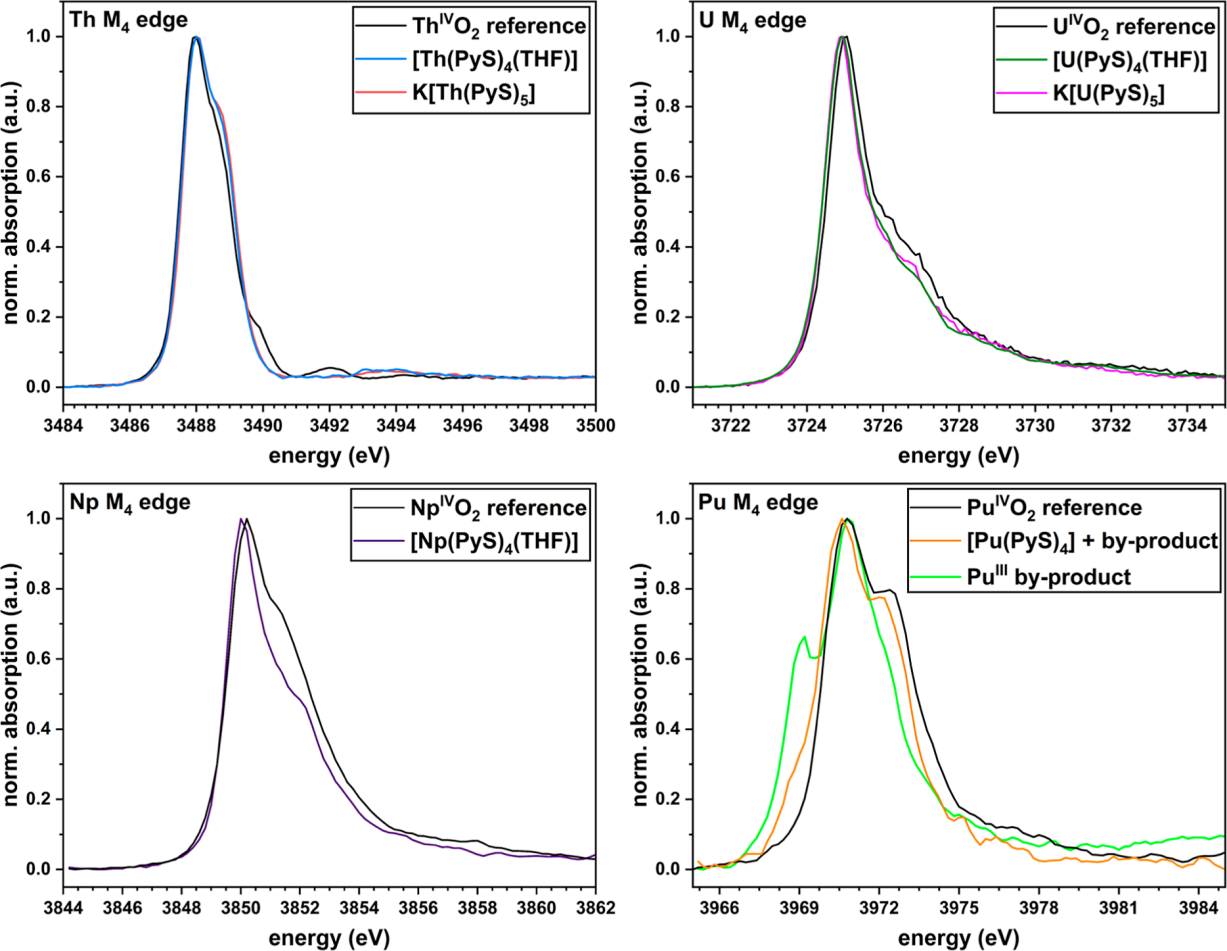
Normalized HERFD-XANES spectra at the M_4_ edge of the An: **1** (blue) and **5** (red) with Th^IV^O_2_ reference (black, *E*_max,Th_: 3488 eV, top left), **2** (green), and **6** (pink) with U^IV^O_2_ reference (black, *E*_max,U_: 3726 eV, top right), **3** (purple) with Np^IV^O_2_ reference (black, *E*_max,Np_: 3850 eV, bottom left), and **4** (orange) with Pu^IV^O_2_ reference (black) and Pu^III^ species (chartreuse) as possible bright green byproduct of the synthesis with PyS–SiMe_3_ (*E*_max,Pu_: 3971 eV, bottom right).

The HERFD-XANES spectrum of **4** exhibits a shoulder at approximately 3969 eV (Figure 8, bottom right), which suggests an impurity of a Pu^III^ species in **4**. The distinction between Pu^III^ and Pu^IV^ species is only possible using secondary maxima (*E*_Pu(III)_: 3969 eV, *E*_Pu(IV)_: 3972 eV), as both main absorption maxima have similar energies (*E*_max,Pu_: 3971 eV). After the reaction of [PuCl_4_(DME)_2_] with 4 eq. PyS–SiMe_3_, only a green insoluble solid could be obtained. The absorption spectrum of this bright green solid shows a high similarity in shape and corresponding energies to the spectrum of PuF_3_ known from literature.^46^ This byproduct is most likely a trivalent Pu complex with PyS ligands, which is supported by the high similarity of the corresponding ATR-IR spectrum with those of **1**–**4** (Figure S42). The reduction of An ions by the PyS ligand is known in the literature,^1^ but for **1**–**3**, i.e., up to Np, the tetravalent oxidation state of the metal centers can be stabilized without byproduct formation. We assume that complex syntheses of [PuCl_4_(DME)_2_] with 2-mercyptopyridyl ligands can involve a redox reaction to a Pu^III^ complex, which is fast with PyS–SiMe_3_, but slow for the salt metathesis reaction with KPyS. We assume that the corresponding oxidation is the formation of 2,2′-dipyridyldisulfide from PyS^–^. However, no experimental proof was observed by IR and NMR spectroscopy. The synthesis of **4** thus represents the limitation of the investigated system, since the formation of a Pu^III^ byproduct cannot be entirely prevented by the choice of reaction method and conditions.

For K­[An­(PyS)_5_] (An: Th (**5**) and U (**6**)), HERFD-XANES measurements were conducted as described before. Similarly, the oxidation states of the metals in **5** and **6** were determined at the An M_4_ edge by comparison with the An^IV^O_2_ references (Figure 8). For both **5** and **6**, the same peak positions as for the respective 1:4 complexes **1** and **2** are found.

Since for [An­(PyS)_4_(THF)] and K­[An­(PyS)_5_] (An: Th and U), both absorption maxima have the same energies, and the shape of the corresponding normalized XAS spectra on the metal side is very similar, the 1:4 and 1:5 complexes exhibit similar electronic properties. In addition, this underlines the advantage of HERFD-XANES measurements to determine the oxidation states of the metal centers precisely and independently from the corresponding ligands, which is of great interest, e.g., with regard to environmental samples.

Also, spin polarization has previously been observed by Yang *et al.* for the PyS^–^ ligand on actinyl moieties with formal reduction at the actinide center.^1^ In the An^IV^ complexes, only very minor spin density was observed at the S atoms (Table 3). This supports that the DFT results used for the bond analysis are suitable for An^IV^ and underlines the oxidation state assignment using HERFD-XANES. Along the actinide series, a slight increase of the average spin density at sulfur can be noted with up to −0.039(7) in [Pu­(PyS)_4_(THF)]. This slightly higher spin density at sulfur in [Pu­(PyS)_4_(THF)] might be the origin of the lower stability of the oxidation state Pu^IV^ in **4** and the observation of Pu^III^ byproduct formation with HERFD-XANES.

**Table 3:** Calculated Spin Densities (a.u.) in [An­(PyS)_4_(THF)] (An: Th–Pu) and [U­(PyS)_5_]^−^ at the Metal Centers and the Donor Atoms of the Ligands, and Spin Contamination ⟨*S* ^2^⟩^a^

	Th	Pa	U	Np	Pu	[U(PyS)_5_]^−^
An spin density	0	0.991	2.036	3.068	4.180	2.042
N spin density	0	–0.001(1)	–0.003(1)	–0.010(6)	–0.011(4)	–0.002(1)
S spin density	0	–0.004(1)	–0.012(3)	–0.017(5)	–0.039(7)	–0.010(2)
O spin density	0	0.001	0.001	0.003	0.003	
⟨*S* ^2^⟩	0	0.75	2.01	3.77	6.07	2.01

a Mean values with standard deviations as errors for N and S.

### Magnetic Measurements of Complexes of the Type [An­(PyS)_4_(THF)] and K­[An­(PyS)_5_]

In order to investigate the magnetic properties of the An complexes with PyS^–^, superconducting quantum interference device (SQUID) magnetometry measurements were performed. Here, the molar magnetic susceptibility χ_mol_ and the effective magnetic moment μ_eff_ were determined in a range of 1.9–300 K (at 3.5 T), and the magnetization *M*_f.u._ of each magnetic center was in a range of ±7 T (at 2 K, see Supporting Information). U^IV^ and Np^IV^, influenced by unpaired 5*f* electrons, were used for the measurements. The Pu complex **4** was not analyzed due to the previously observed occurrence of a trivalent byproduct, which could be neither entirely separated nor accurately quantified, which would have made interpretation of the results impossible. For the An complexes **2** and **3** of the type [An­(PyS)_4_(THF)], the paramagnetic behavior of these compounds can be confirmed by the strongly temperature-dependent behavior of χ_mol_ > 0 vs *T* known from literature (Figure S44 and Table 4).^47,48^ When comparing the two paramagnetic actinides, χ_mol_ for **3** (0.143 emu·mol^–1^·Oe^–1^) is approximately 2.5 times larger than for **2** (0.057 emu·mol^–1^·Oe^–1^) at 1.9 K, as expected for an additional unpaired electron in Np^IV^ (5*f* ^3^) compared to U^IV^ (5*f* ^2^), indicating a stronger magnetization in the external field.^48^

**Table 4:** Results of the SQUID Magnetometry Measurements: Molar Magnetic Susceptibilities χ_mol_ (emu·mol^–1^·Oe^–1^) and Effective Magnetic Moments μ_eff_ (μ_B_) at 1.9 and 300 K^a^

	χ_mol_, 1.9 K	χ_mol_, 300 K	μ_eff_, 1.9 K	μ_eff_, 300 K	*M*_f.u._ (6.5 T)
[U(PyS)_4_(THF)] **2**	0.057	0.003	0.943	2.510	0.51
[Np(PyS)_4_(THF)] **3**	0.143	0.003	1.491	2.623	1.03
K[U(PyS)_5_] **6**	0.011	0.003	0.417	2.575	0.13

a Magnetization of each magnetic center *M*_f.u._ (μ_B_) at 6.5 T, 2 K.

The corresponding μ_eff_ also shows strongly temperature-dependent behavior (Figure 9, left). A 5*f* ^2^ system with a ^3^H_4_ ground state and a 5*f* ^3^ system with a ^4^I_9/2_ ground state would give a magnetic moment of the free ion of 3.578 μ_B_ and 3.618 μ_B_, respectively. As described in the literature, electron–electron interactions *H*_ee_, spin–orbit coupling *H*_SO_ and effects of the ligand field *H*_LF_ play a key role in the description of magnetic behavior. Especially for An^IV^, these influences are described to be approximately equally strong (*H*_ee_ ≈ *H*_SO_ ≈ *H*_LF_).^48–50^ For **2** (Table 4), a μ_eff_-*T*-plot is obtained, and the trend known from literature for tetravalent U complexes with (N,O)-donor ligands could be reproduced.^51^ In contrast to that for U^IV^, a less pronounced temperature dependency of similar U^III^ and U^V^ complexes would be expected. Diamagnetic U^VI^ compounds would require χ_mol_ < 0. Thus, the oxidation state U^IV^ in **2** can be confirmed by using magnetic analysis in addition to HERFD-XANES.

**Figure 9:**
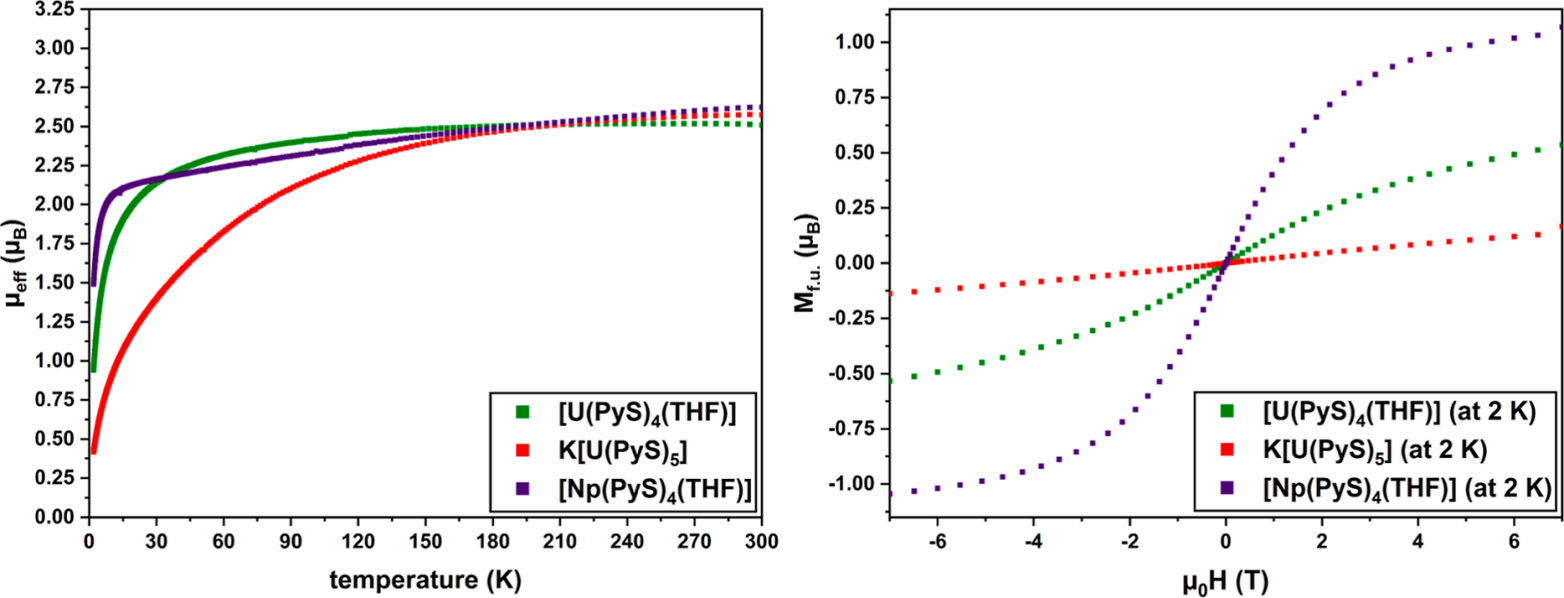
Effective magnetic moments μ_eff_ (μ_B_) plotted against temperature (K) at 3.5 T (left) and magnetization *M*_f.u._ (μ_B_) of each magnetic center plotted against the external magnetic field (T) at 2 K (right) of [U­(PyS)_4_(THF)] **2** (green), [Np­(PyS)_4_(THF)] **3** (purple), and K­[U­(PyS)_5_] **6** (red).

Only limited magnetic data on 9-fold-coordinated U^IV^ complexes is available.^20,52^ In particular, this applies for (*N,S*)-donor ligands, although similar values at 2 K are known for U^IV^ (hydro-)­sulfido complexes (μ_eff_: 1.03 μ_B_, 0.84 μ_B_).^20,52^ For **3**, a strongly temperature-dependent μ_eff_-*T*-plot is obtained as well, but with notable differences to **2**, especially at low temperatures. Here, with decreasing temperature, μ_eff_ is slowly decreasing, dropping sharply at approximately 10 K. At 1.9 K, a higher effective magnetic moment is obtained (1.49 μ_B_) than for **2**, as expected for a ^4^I_9/2_ ground state of a 5*f* ^3^ system.^48^ This leads to a higher molar magnetic susceptibility as described above (Figure S44), but also influences the magnetization *M*_f.u._, which was determined separately at variable magnetic fields (Figure 9, right). For [An(PyS)_4_(THF)], it is strongly dependent on the external magnetic field and tends to saturate nonlinearly for high magnetic fields. A higher magnetization is obtained for **3** (1.03 μ_B_ at 6.5 T) than for **2** (0.51 μ_B_ at 6.5 T) in accordance with high χ_mol_ and μ_eff_ values at 1.9 K for Np^IV^, as described above. The stronger magnetization can be attributed to the additional unpaired electron for 5*f* ^3^ Np^IV^ than for 5*f* ^2^ U^IV^.

Investigating the magnetic properties of K­[U­(PyS)_5_] (**6**) with SQUID magnetometry reveals significant differences to **2**. Here, χ_mol_ is only 0.011 emu·mol^–1^·Oe^–1^ at 1.9 K, which is approximately five times lower than for **2** (Figure S44 and Table 4).^47,48^ While μ_eff_ at 300 K for **6** (2.575 μ_B_) is similar to values for **2** and **3**, a considerably lower effective magnetic moment is obtained at 1.9 K (**6**: 0.417 μ_B_ vs **2**: 0.943 μ_B_, **3**: 1.491 μ_B_). The μ_eff_-*T*-plot (Figure 9, left) also shows significant differences despite the very similar electronic properties of **2** and **6**, as illustrated, e.g., by similar HERFD-XANES spectra (Figure 8, top right). It should be noted that HERFD-XANES was measured at 100 K, where μ_eff_ between **2** and **6** also differs by only about 0.24 μ_B_. An additional PyS ligand does not change the U^IV^ oxidation state of the metal in the entire complex, but an additional shift of electron density from the donor atoms could significantly influence the magnetic behavior. This is reflected in the natural charge of U^IV^, which drops from 0.57 e in **2** to 0.15 e in **6**, and also in the average natural charges of the PyS ligand, though to a lesser degree (**2**: NC_N_ = −0.49(1) e, NC_S_ = −0.11(2) e; **6**: NC_N_ = −0.45(1) e, NC_S_ = −0.10(2) e). Also, the DI_U–N_ = 0.34(2) and DI_U–S_ = 0.48(2) in **2** drops by about 0.05 in both cases in **6** (DI_U–N_ = 0.29(2), DI_U–S_ = 0.43(2)). These seemingly marginal changes in electronics around paramagnetic U^IV^ have a detectable impact on its magnetic behavior. The magnitude of ligand field effects *H*_LF_ on the magnetic parameters for 5f elements is as large as the effects of electron–electron interactions *H*_ee_ and the spin–orbit coupling *H*_SO_, and therefore impacts the μ_eff_-*T*-plot.^48,49^ Furthermore, the *M*_f.u._ of **6** is remarkably less dependent on the external magnetic field than **2** and **3** (Figure 9, right). The magnetization of **6** at 6.5 T and 2 K (0.13 μ_B_) is four times smaller than that for **2**.

## Conclusions

This study advances the understanding of actinide-ligand bonding, addressing key gaps in An coordination chemistry, especially for TRU element complexes with (*N,S*)-donor ligands. Thus, this work contributes to the broad field of actinide coordination chemistry, chemical bond theory, redox behavior, and magnetic properties, offering fundamental insights that may help to optimize ligand design for nuclear fuel partitioning, waste treatment applications, or the decontamination of legacy and accident sites. By synthesizing tetravalent actinide complexes with pyridine-2-thiolate and their comprehensive characterization, supported by quantum chemical calculations, we provide insights into the bonding properties and covalency trends of early An from Th to Pu. The successful synthesis of [An­(PyS)_4_(THF)_n_] (An: Th, U, Np {*n* = 1}, and Pu {*n* = 0}) and homoleptic K­[An­(PyS)_5_] (An: Th and U) complexes highlights the versatility of pyridine-2-thiolate ligands in stabilizing tetravalent actinide species, including the first structurally characterized Np complex with (*N,S*)-donor ligands. Optimized syntheses in THF overcome earlier assumptions about the incompatibility of ether solvents for the complexation of An.^13^ However, challenges remain, as seen in the unsuccessful isolation of a pure Pu^IV^ complex due to redox instability in the presence of a noninnocent ligand. Quantum chemical calculations and experimental trends for [An­(PyS)_4_(THF)_n_] confirm and quantify increasing covalency in An–S interactions with a remarkable peak for Pu–S at 34% covalency. An–N and An–O bonds, on the other hand, remain predominantly ionic. This can contribute to the notably high selectivity of S-containing ligands in the separation of TRU elements.^1–3,5,6,41^ The influence of unpaired 5f electrons was observed by NMR, where paramagnetic shifts are fairly small due to the fast exchange around the actinide centers, which locates the ligands’ protons on average in various positions in the PCS field. However, magnetometric investigation in the solid state demonstrates a pronounced dependency on f-electron count and electronic changes due to increased coordination number around the actinide.

## Experimental Section

### General Comments

*Caution!* The early actinides thorium, uranium, neptunium, and plutonium contain exclusively radioactive isotopes, including long-lived alpha emitters: ^232^Th (*t*_1/2_ = 1.41·10^10^ a), ^235^U (*t*_1/2_ = 7.04·10^8^ a), ^238^U (*t*_1/2_ = 4.47·10^9^ a), ^237^Np (*t*_1/2_ = 2.14·10^6^ a), and ^242^Pu (*t*_1/2_ = 3.75·10^5^ a).^53^ Special safety requirements are necessary for the safe handling of radioactive substances. These include certified laboratories with the appropriate equipment. All experiments were carried out in the controlled laboratory at the Institute of Resource Ecology, Helmholtz-Zentrum Dresden—Rossendorf. The metal precursors [ThCl_4_(DME)_2_], UCl_4_, [NpCl_4_(DME)_2_], and [PuCl_4_(DME)_2_] used for the complex syntheses were prepared following procedures known from the literature.^54–56^ All syntheses and experimental methods were carried out under N_2_ inert gas atmosphere in glove boxes (*MBraun*) or using conventional Schlenk techniques, if not stated otherwise. Synthetic details are given in the Supporting Information. The required chemicals were used as received. Acetonitrile, diethyl ether, *n*-pentane, and tetrahydrofuran were dried with an SPS-5 plant by *MBraun* using a system of two Al_2_O_3_ columns. Dichloromethane was distilled using calcium hydride. Deuterated THF was dried with sodium and benzophenone and then distilled. All solvents used were stored over a 3 Å molecular sieve.

### Single-Crystal X-ray Diffraction

Crystals suitable for SC-XRD were transferred to mineral oil and cut into fragments under a microscope. These were placed on a *MiTeGen MicroMount* sample holder and measured in a 100 K *Oxford Cryostream N*_2_ with a *Bruker D8 Venture* diffractometer equipped with a *Photon II* detector using Mo K_α_ X-rays (λ = 0.71073 Å). Data processing was carried out using the *Bruker Apex 4* software package. For data of the single crystals, data collection, absorption correction, and refinement, see Tables S6 and S7. The crystal structures were solved using the SHELXT software package.^57,58^

### High Energy-Resolution Fluorescence-Detected X-ray Spectroscopy

The solid samples of the An complexes were placed on the adhesive surfaces of the sample holders and were sealed with Kapton foil. To prevent humidity and oxygen from affecting the samples, a Dewar container with liquid nitrogen or an inert gas container with a nitrogen atmosphere was used as a transport container. The X-ray absorption spectra were recorded at the ROBL beamline of the ESRF in Grenoble, France,^59^ under cryo-conditions with a cryostat. The incident energy was obtained from the ⟨111⟩ reflection at a double Si monochromator. The suppression of higher harmonic oscillations was achieved by the total reflection of two Si mirrors. XANES spectra were measured in HERFD mode using an X-ray emission spectrometer^44^ with the sample, analyzer crystal, and photon detector (Ketek detector) arranged in a vertical *Rowland* geometry.

### NMR Spectroscopy

Solid samples were dissolved in 500–700 μL deuterated solvents (THF-*d*_8_, DCM-*d*_2_) from *Deutero GmbH*, and the 10–120 mM solutions were tightly sealed in *Young* NMR tubes. NMR spectra were recorded using a *Varian Inova 400* spectrometer with a *Varian AutoX ID* probe head at 30 °C (303 K) and an *Agilent VNMRS 400 DD2* spectrometer equipped with an *Agilent ONE* probe head at 25 °C (298 K), respectively, if not stated otherwise. The measurements were performed at resonance frequencies of 399.9 MHz (*Varian*) or 401.8 MHz (*Agilent*) for ^1^H and 100.6 MHz (*Varian*) or 101 MHz (*Agilent*) for ^13^C. Furthermore, 2D correlated ^1^H,^1^H–COSY, ^1^H,^13^C-HMBC, and ^1^H,^13^C-HSQC spectra were recorded using standard pulse sequences.

### Quantum Chemical Calculations

For complexes of the type [An­(PyS)_4_(THF)], the software ORCA 5.0.3 ^60^ with density functional theory (functional: PBE0 [open-shell], basis sets: SARC-ZORA-TZVPP [for An], ZORA-def2-TZVPP [for all other atoms])^61,62^ was used to determine an optimized geometry (including all electrons). The scalar-relativistic ZORA^63,64^ Hamilton operator, a D3BJ dispersion correction,^65,66^ and the conductor-like screening model (COSMO, ε = 8.9, *rsolv* = 2.94) were used to take solvent effects into account. Based on numerically calculated IR spectra, it was confirmed that the geometry optimizations found do not represent transition states. An NBO/NLMO analysis was carried out on this basis,^67^ and a QTAIM analysis was performed with MULTIWFN^68^ on the same basis. IQA analysis was performed with AIMALL, version 19.10.12.^69^

### Superconducting Quantum Interference Device Magnetometry Measurements

Temperature-dependent measurements (1.9 K–300 K) were carried out on an *MPMS3* SQUID magnetometer from *Quantum design.* Raw data were recorded as SQUID voltage vs sample position. Kel-F sample holders were attached to quartz glass holders with *Ge-Varnish*. Analog measurements (35000 Oe, 30 mm sample movement) were carried out for the blank (empty sample holder) and the actinide complexes. The *SquidLab* program (version: 2.9.1) was used for background correction and data adjustment using the *Levenberg–Marquardt* method.^70^ A Pd standard was used to determine the calibration factor. The data obtained (DC moment vs temperature) was analyzed, as described in the Supporting Information.

### Infrared Spectroscopy

ATR-FT-IR spectra were recorded in a range of 4000–650 cm^–1^ with a *Cary 630-FTIR* spectrometer from *Agilent technologies* at a resolution of 1 cm^–1^. Solid samples were pressed onto the measuring crystal with a screw stamp. Dissolved samples were placed in solution on the measuring crystal, and the solvent was evaporated in the glovebox atmosphere.

## Supplementary Material



## Notes

Safety statement, further experimental and physical methods, NMR spectra and VT-NMR data, molecular structures of bis­(2-pyridylthio)­methane and [Np­(PyS)_4_(PySH)], crystallographic information, quantum chemical calculation details, IR spectra, SQUID: χ_mol_ *vs T* plot and experimental details, ASAP-APCI mass spectra, and elemental analysis results (PDF)

## Notes

Dedicated to Prof. Dr. Thomas Fanghänel on the occasion of his 70th birthday.
