# Supplementary material for: Bonding Trends in Pyridine-2-thiolato Complexes of Tetravalent Actinides
Source: Inorg Chem. 2025 Oct 23;64(44):21919–31. doi: 10.1021/acs.inorgchem.5c03369 (PMC12606716; doi:10.1021/acs.inorgchem.5c03369)
Supplement: Supplementary file 1 [file ic5c03369_si_001.pdf]

Supporting Information

Bonding trends in pyridine-2-thiolato complexes of tetravalent actinides

*Johannes Balas,[a] Christian Urbank,[a,b] Peter Kaden,[a] Michael Patzschke,[a] Juliane März,[a] Kristina Kvashnina,[a,c] Moritz Schmidt,[a,d] Thorsten Stumpf,[a] Robert Gericke[a]**

[a]Helmholtz-Zentrum Dresden – Rossendorf, Institute of Resource Ecology, Bautzner Landstraße 400, 01328 Dresden, Germany.

* e-mail: r.gericke@hzdr.de

[b]Institute of Inorganic and Applied Chemistry, University of Hamburg, Martin-Luther-King-Platz 6, 20146 Hamburg, Germany.

[c]The Rossendorf Beamline at ESRF – The European Synchrotron, CS40220, 38043 Grenoble Cedex 9, France.

[d]Brandenburg University of Technology Cottbus-Senftenberg, Institute of Materials Chemistry, 01968 Senftenberg, Germany.

Contents

1. Experimental Section 3
2. NMR characterization of [Th($PyS)_4$(THF)] 10
3. NMR characterization of [U($PyS)_4$(THF)] 13
4. NMR characterization of [Np($PyS)_4$(THF)] 16
5. NMR characterization of [Pu($PyS)_4$] / [Pu($PyS)_4$(THF)] 19
6. NMR characterization of K[Th($PyS)_5$] 22
7. NMR characterization of K[U($PyS)_5$] 25
8. NMR characterization of the K[Np($PyS)_5$] reaction mixture 26
9. NMR characterization of [An($PyS)_4$($THF)_n$] with possible solvent molecule coordination 29
10. NMR characterization of solvent-free complexes [An($PyS)_4$] in DCM-d_2 30
11. Single-crystal X-ray diffraction 31
12. Quantum chemical calculations 35
13. Infrared spectroscopy 59
14. Superconducting quantum interference device (SQUID) magnetization measurements 61
15. ASAP-APCI mass spectrometry 62

1. Experimental Section

Caution! The early actinides thorium, uranium, neptunium and plutonium contain exclusively radioactive isotopes, including long-lived alpha emitters: ^{232}Th ($t_{1/2} = 1.41 \cdot 10^{10}$ a), ^{235}U ($t_{1/2} = 7.04 \cdot 10^{8}$ a), ^{238}U ($t_{1/2} = 4.47 \cdot 10^{9}$ a), ^{237}Np ($t_{1/2} = 2.14 \cdot 10^{6}$ a) and ^{242}Pu ($t_{1/2} = 3.75 \cdot 10^{5}$ a).[1] Special safety requirements are necessary for the safe handling of radioactive substances. These include certified laboratories with appropriate equipment. All experiments were carried out in the controlled laboratory at the Institute of Resource Ecology, Helmholtz-Zentrum Dresden – Rossendorf.

APCI-APCI mass spectrometry. The mass spectra of the solid samples were recorded with an *Expression L* compact mass spectrometer (CMS) from *Advion interchim* in a measuring range of 10 – 2000 *m/*z. Small amounts of the solid samples were picked up with a heated glass capillary, injected (gas temperature (°C)/corona needle discharge (µA): KPyS: 350/5, **1**: 350/5, **2**: 400/5, **3**: 350/5, **4**: 400/5, **5**: 400/5, **6**: 350/5) through an ASAP-APCI ionization source (source temperature (°C)/source voltage (V): KPyS: 200/160, **1**: 200/160, **2**: 250/120, **3**: 200/120, **4**: 250/180, **5**: 250/120, **6**: 200/120) and the charged fragments were detected in positive and negative measurement mode.

Elemental analysis. The percentages of C, H, N and S in the solid samples were determined using a *Vario Micro Cube* from *Elementar*. The data was recorded in a helium gas flow for sample masses between 1.9 mg and 2.0 mg. Sulfanilamide ($C_6H_8N_2O_2S$) was used as a standard and correction factors for the deviations from the calculated proportions were determined. Samples with Np and Pu could not be measured for radiation protection reasons.

The **NMR signals** are assigned using the following numbers:

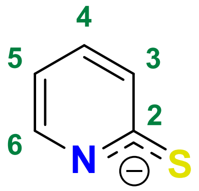

Potassium pyridine-2-thiolate (KPyS). In a cold mixture of acetone and liquid nitrogen at −30 °C, 20 mL DCM was added to 2.99 g (0.027 mol) 2-mercaptopyridine and 1.00 g (0.025 mol) potassium hydride. The yellow reaction mixture was stirred until room temperature was reached and for a further 18 h, forming a colorless precipitate. Then an additional 30 mL of DCM was added and the reaction mixture was stirred for another 15 hours. A colorless solid was filtered off from a light-yellow solution, washed six times with 5 mL DCM and dried for 3 h at $6.2 \cdot 10^{-2}$ mbar. Yield: 3.36 g (84%). ^{1}H NMR: (THF-d_8, 399.9 MHz, 303 K) δ = 6.21 (1H), 6.75 (1H), 7.12 (1H), 7.68 ppm (1H), ATR-IR: ν (cm^{-1}) 1572 (s), 1535 (s), 1480 (vw), 1438 (s), 1400 (s), 1362 (m), 1277 (m), 1266 (m), 1217 (w), 1171 (m), 1144 (w), 1124 (s), 1077 (m), 1040 (m), 985 (w), 973 (s), 956 (m), 875 (w), 872 (w), 808 (w), 760 (s), 747 (vs), 737 (s), 725 (s), 682 (w), APCI(−)-MS: *m/z* 110: PyS^{-}.

2-(trimethylsilylmercapto)pyridine (PyS–SiMe$_3$). To a light-yellow solution of 999.8 mg (8.996 mmol) 2-mercaptopyridine in 15 ml THF, 2.1 mL (15.0 mmol) triethylamine was added at 0 °C and stirred for 2 min. While cold, 1.2 mL (9.2 mmol) of trimethylsilyl chloride was added dropwise, forming a colorless precipitate. The pale-yellow reaction mixture was stirred until room temperature was reached and for a further 48 h. A colorless solid was filtered off from the light-yellow solution and washed three times with 2 mL THF. The solvent was then removed from the filtrate under vacuum. The remaining yellow liquid was distilled in vacuo (bp: 32 °C,

$1.6\cdot10^{-1}$ mbar). Yield: 1086.2 mg (66%). ^{1}H NMR: (THF-d_8, 401.8 MHz, 298 K) δ = 0.44 (9H, $Si(CH_3)_3$), 6.99 (1H, H-5), 7.26 (1H, H-3), 7.46 (1H, H-4) 8.29 ppm (1H, H-6).

[Th(PyS)$_4$(THF)] 1 (A) A colorless solution of 29.9 mg (0.054 mmol, 1 eq) [$ThCl_4$(DME)$_2$] in 2 mL THF was added dropwise to 32.3 mg (0.216 mmol, 4 eq) colorless KPyS. A color change to a light-yellow suspension occurred immediately. The reaction mixture was stirred for 24 h at ambient temperature. Slightly yellow colored potassium chloride was then centrifuged off. The volume of the remaining light yellow, clear solution was concentrated to approx. 800 µL under a stream of glove box atmosphere. A colorless solid was then precipitated with 2 mL *n*-pentane, which was centrifuged off and dried for 2 h at $1\cdot10^{-2}$ mbar. Yield: 38.3 mg (95%). Crystals suitable for SC-XRD (colorless blocks) were obtained by slow evaporation of a THF solution layered with cyclohexane in the glove box atmosphere after 2 d. (B) A light-yellow solution of 43.7 mg (0.238 mmol, 4.4 eq) PyS–$SiMe_3$ in 0.874 mL THF was added to 29.8 mg (0.054 mmol, 1 eq) colorless [$ThCl_4$(DME)$_2$]. The pale-yellow reaction mixture was stirred for 1 h at ambient temperature. After addition of 100 µL *n*-pentane and centrifugation, colorless crystals were obtained by evaporation of the solution in the glove box atmosphere. Yield: 28.2 mg (70%). ^{1}H NMR: (THF-d_8, 399.9 MHz, 303 K) δ = 6.72 (4H, H-5), 7.02 (4H, H-3), 7.30 (4H, H-4), 8.37 ppm (4H, H-6), ^{13}C NMR: (THF-d_8, 100.6 MHz, 303 K) δ = 117.33 (C-5), 131.30 (C-3), 137.91 (C-4), 146.17 (C-6), 174.76 ppm (C-2), EA: (%) found C 37.47, H 2.84, N 7.36, S 17.83, calculated for [Th(PyS)$_4$]·0.5 THF C 37.28, H 2.84, N 7.91, S 18.10, HERFD-XANES: (E_{max} M_4) 3488 eV, ATR-IR: ν (cm^{-1}) 1586 (vs), 1543 (m), 1506 (vw), 1473 (vw), 1441 (s), 1411 (vs), 1368 (m), 1262 (s), 1233 (m), 1180 (m), 1153 (sh), 1132 (vs), 1096 (m), 1085 (m), 1040 (s), 1020 (sh), 1001 (s), 987 (m), 921 (w), 875 (w), 840 (m), 820 (m), 753 (vs), 728 (vs), APCI(+)-MS: *m/z* 673: ^{232}Th(PyS)$_4$ + H^+.

[U(PyS)$_4$(THF)] 2 (A) A dark green solution of 50.0 mg (0.132 mmol, 1 eq) UCl_4 in 2 mL THF was added dropwise to 78.7 mg (0.527 mmol, 4 eq) colorless KPyS. A color change to a brown suspension occurred immediately. The reaction mixture was stirred for 22 h at room temperature. Slightly yellow-brown colored potassium chloride was then centrifuged off. The volume of the remaining brown-yellow, clear solution was concentrated to approx. 800 µL under a stream of glove box atmosphere. An ochre-yellow solid was then precipitated with 2 mL *n*-pentane, which was centrifuged off and dried for 2.2 h at $1 \cdot 10^{-2}$ mbar. Yield: 58.1 mg (59%). Orthorhombic crystals suitable for SC-XRD (yellow blocks) were obtained by slow evaporation of a THF solution in the glove box atmosphere after 2 d. Triclinic crystals (yellow blocks) were obtained by slow evaporation of a THF solution of **6** in the glove box atmosphere after 2 d. (B) A light-yellow solution of 42.5 mg (0.232 mmol, 4.4 eq) PyS–$SiMe_3$ in 0.850 mL THF was added to 19.9 mg (0.053 mmol, 1 eq) dark green UCl_4. The brown-yellow reaction mixture was stirred for 1 h at room temperature. After addition of 100 µL *n*-pentane and centrifugation, yellow crystals were obtained by evaporation of the solution in the glove box atmosphere. Yield: 20.6 mg (53%). ^{1}H NMR: (THF-d$_8$, 399.9 MHz, 303 K) $\delta = 5.67$ (4H, H-5), 8.09 (4H, H-4), 8.62 (4H, H-3), 16.47 ppm (4H, H-6), ^{13}C NMR: (THF-d$_8$, 100.6 MHz, 303 K) $\delta = 111.56$ (C-3), 134.13 (C-4), 142.55 (C-5), 143.53 (C-6), C-2 not observed, EA: (%) found C 34.78, H 2.32, N 7.45, S 18.04, calculated for [U(PyS)$_4$]·0.5 THF C 36.97, H 2.82, N 7.84, S 17.94, HERFD-XANES: (E_{max} M_4) 3725 eV, (E_{max} L_3) 17169 eV, ATR-IR: ν (cm^{-1}) 1585 (s), 1545 (m), 1504 (w), 1473 (vw), 1441 (s), 1411 (vs), 1370 (m), 1261 (s), 1235 (m), 1222 (sh), 1181 (w), 1153 (sh), 1133 (vs), 1095 (m), 1086 (m), 1040 (s), 1019 (sh), 1002 (s), 983 (sh), 919 (w), 873 (w), 838 (m), 823 (m), 752 (vs), 729 (vs), APCI(+)-MS: *m/z* 679: ^{nat}U(PyS)$_4$ + H$^+$.

[Np(PyS)$_4$(THF)] 3 A light-yellow solution of 28.9 mg (0.157 mmol, 4.4 eq) PyS–SiMe$_3$ in 0.577 mL THF was added to 20.0 mg (0.036 mmol, 1 eq) pink [NpCl$_4$(DME)$_2$]. The dark red reaction mixture was stirred for 1 h at room temperature. After addition of 100 µL *n*-pentane and centrifugation, dark red crystals suitable for SC-XRD were obtained by evaporation of the solution in the glove box atmosphere after 2 d. Yield: 18.1 mg (67%). ^{1}H NMR: (THF-d$_8$, 401.8 MHz, 298 K) δ = 6.94 (4H, H-4), 7.00 (4H, H-5), 9.57 (4H, H-3), 13.65 ppm (4H, H-6), ^{13}C NMR: (THF-d$_8$, 101 MHz, 298 K) δ = 97.13 (C-3), 136.25 (C-4), 152.36 (C-5), 154.85 (C-6), C2 not observed, HERFD-XANES: (E_{max} M$_4$) 3850 eV, ATR-IR: ν (cm^{-1}) 1584 (s), 1545 (m), 1504 (vw), 1473 (vw), 1440 (s), 1412 (vs), 1369 (w), 1262 (s), 1235 (w), 1222 (sh), 1183 (w), 1151 (sh), 1134 (vs), 1097 (m), 1085 (m), 1040 (s), 1019 (sh), 1002 (s), 988 (sh), 914 (w), 871 (w), 839 (m), 824 (w), 752 (s), 730 (s), APCI(+)-MS: *m/z* 567: ^{237}Np(PyS)$_3^+$, *m/z* 678: ^{237}Np(PyS)$_4$ + H$^+$, *m/z* 1244: (^{237}Np(PyS)$_3$)$_2$(PyS)$^+$.

[Pu(PyS)$_4$] 4 A red-orange solution of 12.5 mg (0.022 mmol, 1 eq) [PuCl$_4$(DME)$_2$] in 900 µL THF was added to 13.2 mg (0.089 mmol, 4 eq) colorless KPyS. A color change via gray-brown to a green-gray suspension occurred immediately. The reaction mixture was stirred for 24 h at room temperature. Slightly green colored potassium chloride was then centrifuged off. The solvent of the remaining orange, clear solution was evaporated in the atmosphere of the underpressure glove box. An orange-brown solid was obtained and dried for 2 h at $1\cdot10^{-2}$ mbar. Yield: 5.2 mg (34%). ^{1}H NMR: (DCM-d$_2$, 399.9 MHz, 223 K) δ = 7.08 (4H, H-5), 7.49 (4H, H-3), 7.56 (4H, H-4), 8.37 ppm (4H, H-6), ^{1}H NMR: (THF-d$_8$, 401.8 MHz, 298 K) δ = 7.05 (4H, H-5), 7.54 (8H, H-3 & H-4), 8.33 ppm (4H, H-6), ^{13}C NMR: (DCM-d$_2$, 100.6 MHz, 303 K) δ = 119.48 (C-3), 121.08 (C-5), 137.26 (C-4), 149.49 (C-6), 158.68 (C-2), HERFD-XANES: (E_{max} M$_4$) 3971 eV, ATR-IR: ν (cm^{-1}) 1582 (s), 1548 (m), 1480 (sh), 1440 (s), 1414 (vs), 1362 (w), 1265 (s), 1234 (w), 1169 (w),

1148 (sh), 1131 (vs), 1083 (m), 1043 (s), 1004 (s), 991 (sh), 965 (w), 875 (m), 850 (m), 824 (w), 752 (s), 726 (s), APCI(+)-MS: *m/z* 682: $^{242}Pu(PyS)_4 + H^+$.

K[Th(PyS)$_5$] 5 A colorless solution of 29.9 mg (0.054 mmol, 1 eq) [$ThCl_4(DME)_2$] in 1 mL THF was added dropwise to 40.5 mg (0.271 mmol, 5 eq) colorless KPyS. A color change to a light-yellow suspension occurred immediately. The reaction mixture was stirred for 20 h at room temperature. Brownish colored potassium chloride was then centrifuged off. The solvent of the remaining yellow, clear solution was evaporated under a stream of glove box atmosphere. A yellow solid was obtained and dried for 2 h at $1\cdot10^{-2}$ mbar. Yield: 28.2 mg (63%). Crystals suitable for SC-XRD of K[Th(PyS)$_5$]·2.075 (DCM) (colorless needles) were obtained by vapor diffusion of a DCM solution with diethyl ether at ambient temperature in the glove box after 3 d. ^1H NMR: (THF-d$_8$, 399.9 MHz, 303 K) δ = 6.46 (4H, H-5), 6.73 (4H, H-3), 7.02 (4H, H-4), 8.39 ppm (4H, H-6), ^{13}C NMR: (THF-d$_8$, 100.6 MHz, 303 K) δ = 115.39 (C-5), 120.37 (C-4), 135.68 (C-3), 146.88 (C-6), 176.72 ppm (C-2), EA: (%) found C 39.85, H 3.39, N 7.61, S 17.51, calculated for K[Th(PyS)$_5$]·1.4 THF C 39.83, H 3.41, N 7.59, S 17.37, HERFD-XANES: (E_{max} M_4) 3488 eV, ATR-IR: ν (cm^{-1}) 1615 (w), 1584 (s), 1568 (m), 1543 (s), 1491 (w), 1441 (s), 1409 (vs), 1364 (m), 1261 (s), 1231 (m), 1182 (m), 1131 (vs), 1085 (m), 1053 (m), 1040 (m), 1000 (m), 986 (m), 914 (sh), 897 (m), 865 (sh), 748 (vs), 728 (vs), 700 (sh), APCI(+)-MS: *m/z* 562 $^{232}Th(PyS)_3{}^+$, *m/z* 673: $^{232}Th(PyS)_4 + H^+$, *m/z* 796: $K^{232}Th(PyS)_4(C_3H_2NS) + H^+$.

K[U(PyS)$_5$] 6 A dark green solution of 49.9 mg (0.132 mmol, 1 eq) UCl_4 in 1 mL THF was added dropwise to 98.6 mg (0.661 mmol, 5 eq) colorless KPyS. A color change to an ochre-yellow suspension occurred immediately. The reaction mixture was stirred for 23 h at room temperature. Brownish colored potassium chloride was then centrifuged off and washed with 1 mL THF. The solvent of the remaining dark yellow, clear solution was evaporated under a stream of glove box

atmosphere. An ochre-yellow solid was obtained and dried for 2 h at $1 \cdot 10^{-2}$ mbar. Yield: 91.7 mg (84%). In an attempt to grow crystals suitable for SC-XRD, triclinic crystals (yellow blocks) of **2** were obtained by slow evaporation of a THF solution of **6** in the glove box atmosphere after 2 d. ^{1}H NMR: (THF-d_8, 399.9 MHz, 303 K, broad signals) $\delta = 5.33$ (4H), 8.16 (4H), 8.74 (4H), 11.69 ppm (4H), EA: (%) found C 39.46, H 2.95, N 7.98, S 18.57, calculated for $K[U(PyS)_5] \cdot 0.7$ THF C 38.01, H 2.94, N 7.97, S 18.25, HERFD-XANES: (E_{max} L_3) 17169 eV, ATR-IR: ν (cm^{-1}) 1615 (w), 1582 (s), 1575 (s), 1543 (s), 1497 (sh), 1439 (s), 1409 (vs), 1366 (m), 1261 (s), 1232 (m), 1180 (m), 1129 (vs), 1053 (m), 1084 (m), 1039 (m), 998 (m), 984 (m), 914 (sh), 892 (m), 865 (w), 748 (vs), 727 (vs), 700 (sh), APCI(−)-MS: *m/z* 788: $^{nat}U(PyS)_5^{-}$.

2. NMR characterization of [Th(PyS)$_4$(THF)]

^{1}H NMR: (THF-d$_8$, 399.9 MHz, 303 K) δ = 6.72 (4H, H-5), 7.02 (4H, H-3), 7.30 (4H, H-4), 8.37 ppm (4H, H-6), ^{13}C NMR: (THF-d$_8$, 100.6 MHz, 303 K) δ = 117.33 (C-5), 131.30 (C-3), 137.91 (C-4), 146.17 (C-6), 174.76 ppm (C-2).

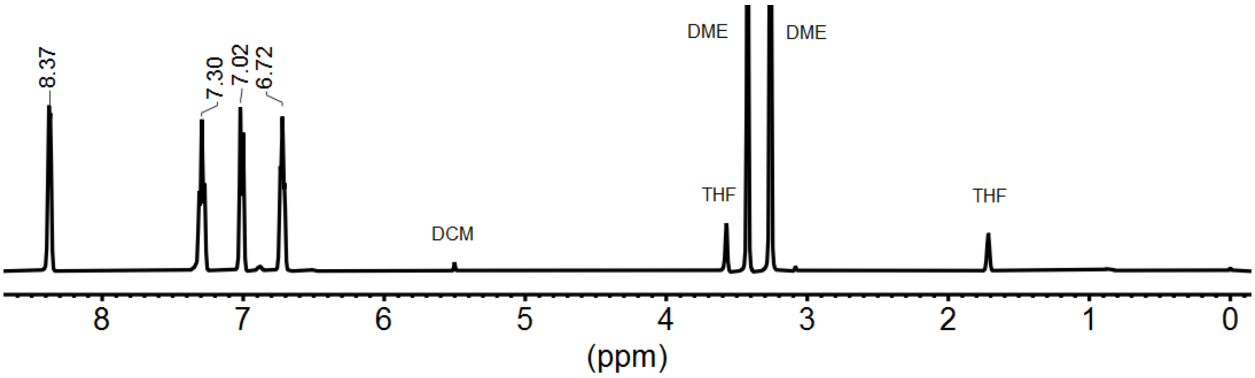

Figure S1. ^{1}H NMR spectrum of the reaction mixture of [Th(PyS)$_4$(THF)] synthesized with KPyS after centrifugation in THF-d$_8$ at 303 K.

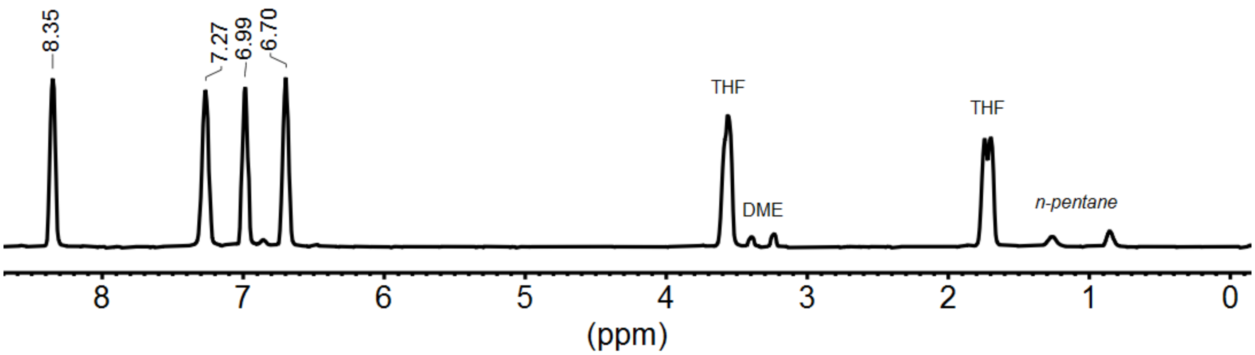

Figure S2. ^{1}H NMR spectrum of [Th(PyS)$_4$(THF)] synthesized with PyS–SiMe$_3$ in THF-d$_8$ at 298 K.

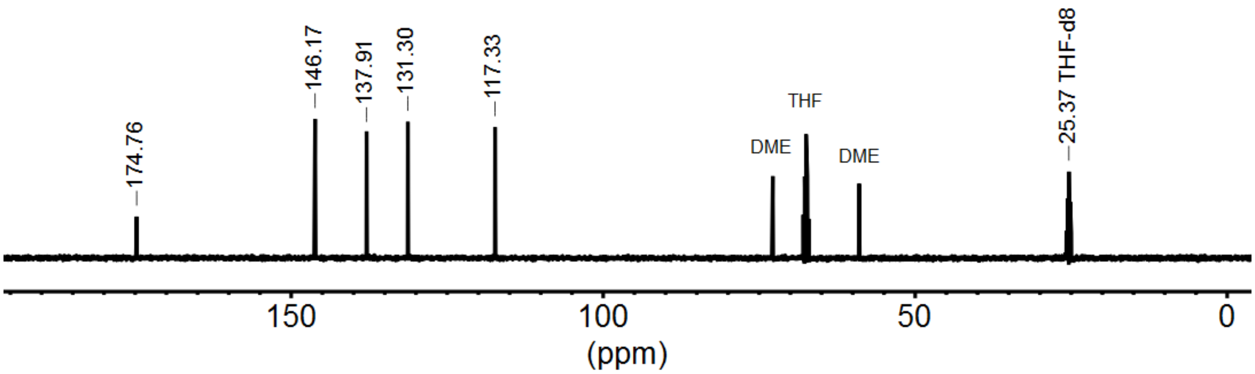

Figure S3. ^{13}C NMR spectrum of the reaction mixture of [Th(PyS)$_4$(THF)] synthesized with KPyS after centrifugation in THF-d$_8$ at 303 K.

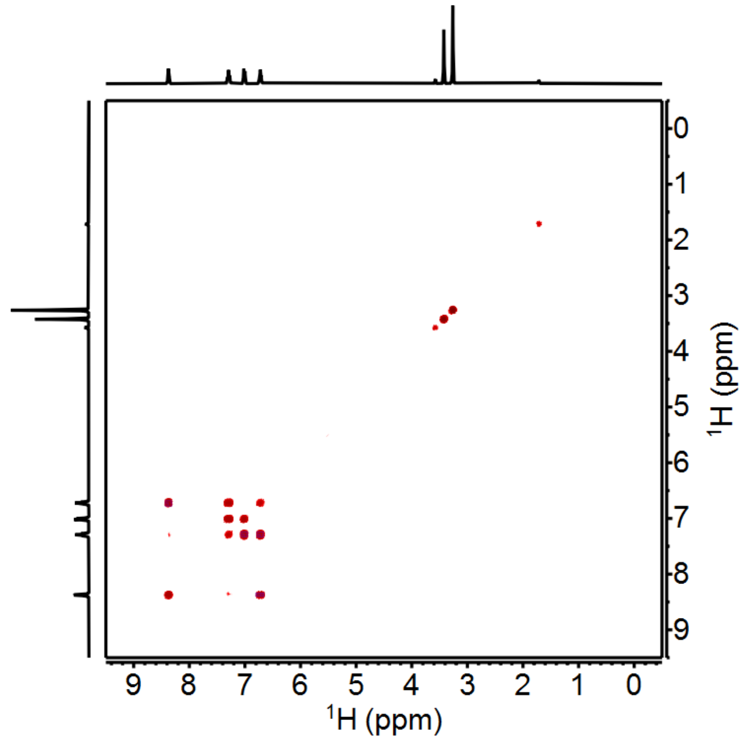

Figure S4. ^{1}H-^{1}H-COSY spectrum of [Th(PyS)$_4$(THF)] in THF-d$_8$ at 303 K.

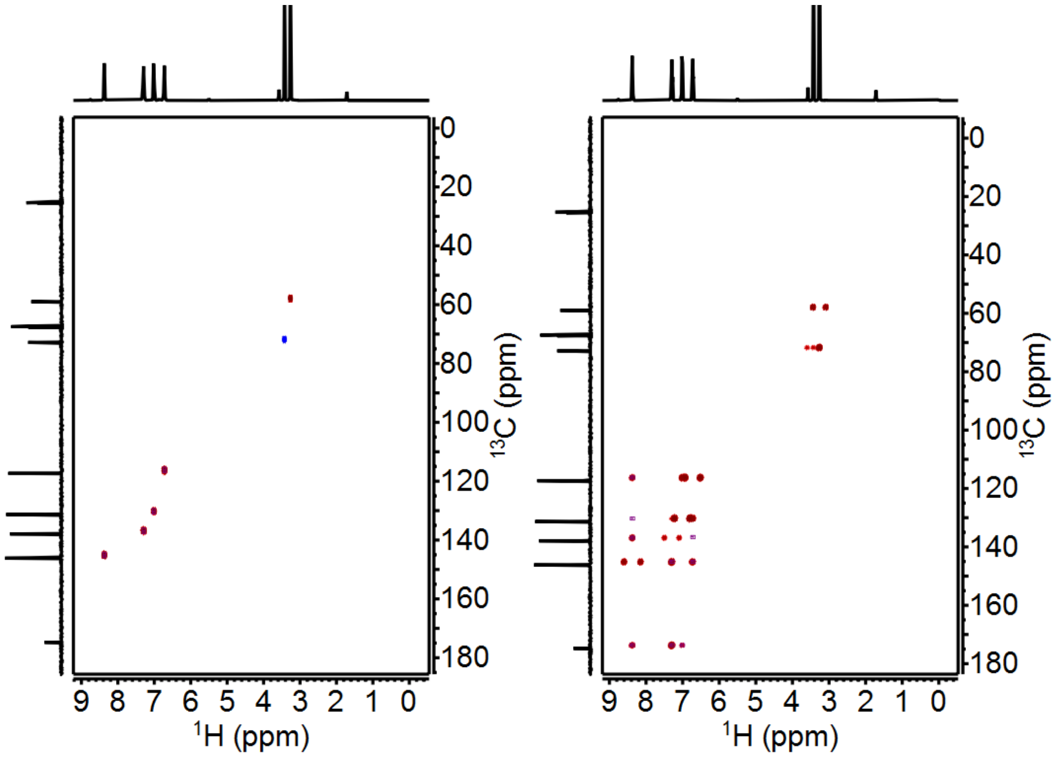

Figure S5. ^{1}H-^{13}H-HSQC spectrum (left) and ^{1}H-^{13}H-HMBC spectrum (right) of [Th(PyS)$_4$(THF)] in THF-d$_8$ at 303 K.

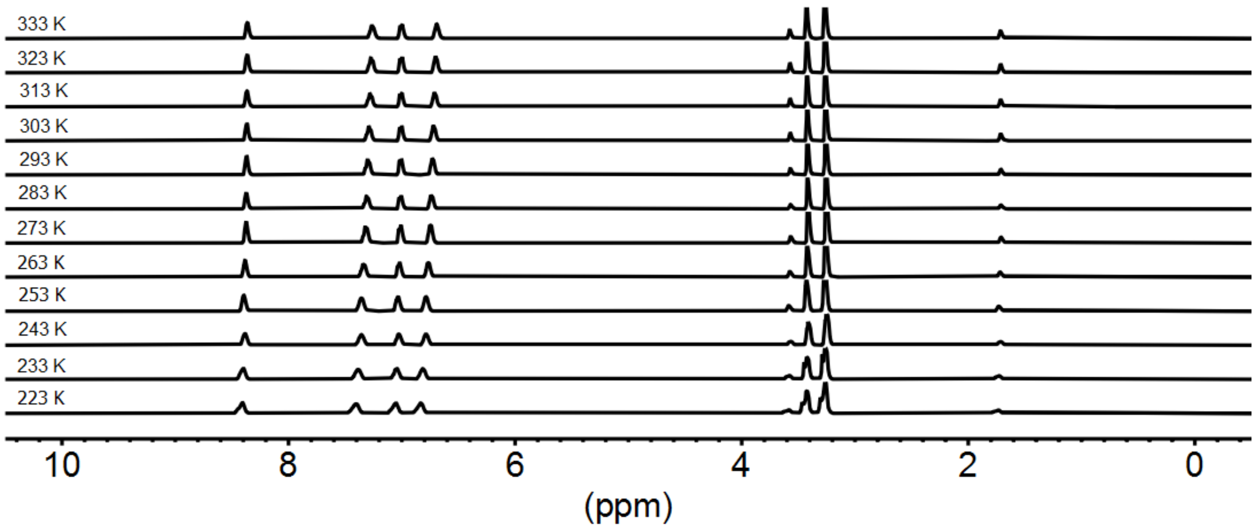

Figure S6. ^{1}H NMR temperature series of the reaction mixture of [Th(PyS)$_4$(THF)] synthesized with KPyS after centrifugation in THF-d$_8$ from 223 K to 333 K.

Table S1. NMR chemical shifts of [Th(PyS)$_4$(THF)] in THF-d$_8$ referenced on TMS at various temperatures.

Temperature (K)	H-3 (ppm)	H-4 (ppm)	H-5 (ppm)	H-6 (ppm)
**333**	7.00	7.26	6.69	8.36
**323**	7.00	7.27	6.70	8.36
**313**	7.01	7.28	6.71	8.36
**303**	7.02	7.30	6.72	8.37
**293**	7.02	7.30	6.73	8.37
**283**	7.02	7.31	6.74	8.37
**273**	7.02	7.32	6.75	8.37
**263**	7.02	7.32	6.77	8.38
**253**	7.03	7.36	6.78	8.39
**243**	7.03	7.36	6.78	8.39
**233**	7.04	7.39	6.81	8.40
**232**	7.05	7.41	6.84	8.41

3. NMR characterization of [U(PyS)$_4$(THF)]

^{1}H NMR: (THF-d$_8$, 399.9 MHz, 303 K) δ = 5.67 (4H, H-5), 8.09 (4H, H-4), 8.62 (4H, H-3), 16.47 ppm (4H, H-6), ^{13}C NMR: (THF-d$_8$, 100.6 MHz, 303 K) δ = 111.56 (C-3), 134.13 (C-4), 142.55 (C-5), 143.53 (C-6), C2 not observed.

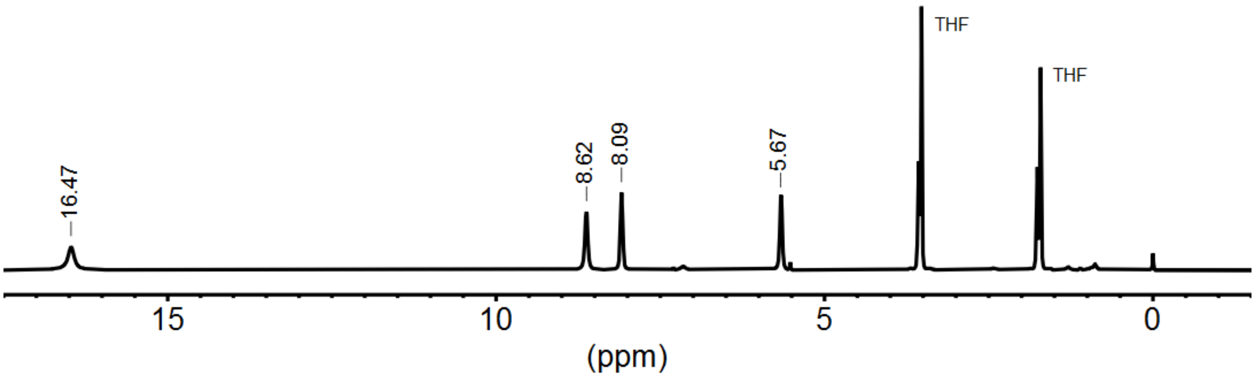

Figure S7. ^{1}H NMR spectrum of [U(PyS)$_4$(THF)] synthesized with KPyS in THF-d$_8$ at 303 K.

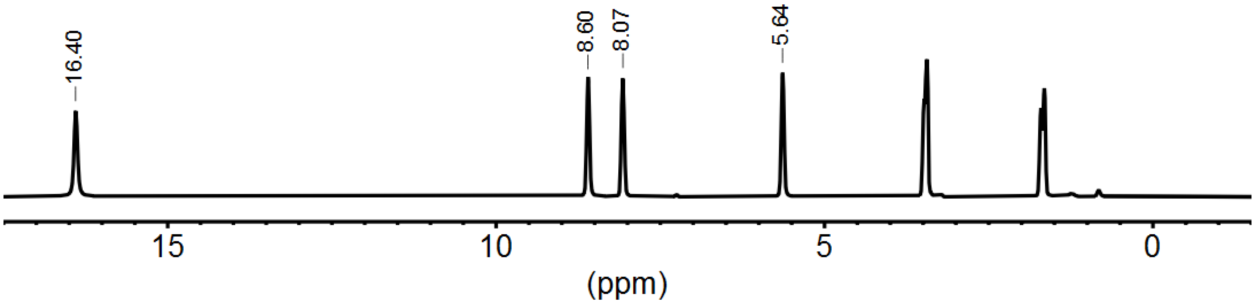

Figure S8. ^{1}H NMR spectrum of [U(PyS)$_4$(THF)] synthesized with PyS–SiMe$_3$ in THF-d$_8$ at 298 K.

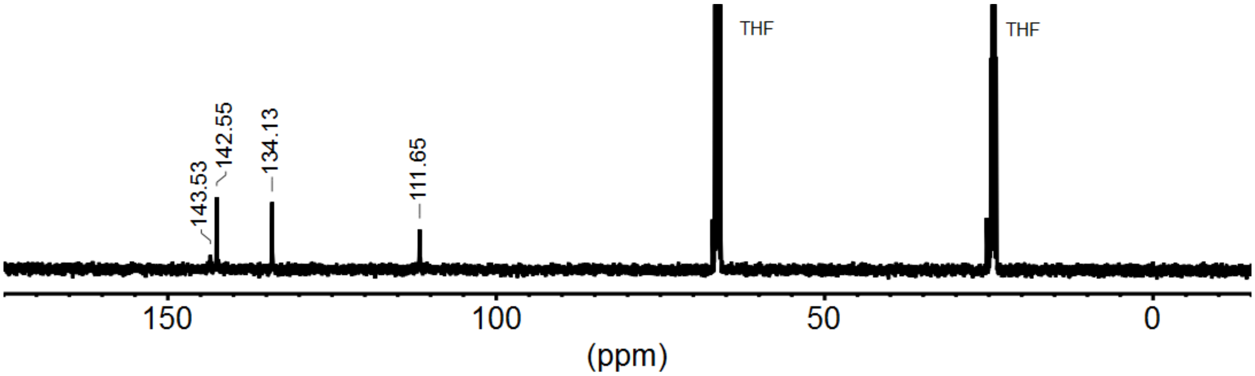

Figure S9. ^{13}C NMR spectrum of [U(PyS)$_4$(THF)] synthesized with KPyS in THF-d$_8$ at 303 K.

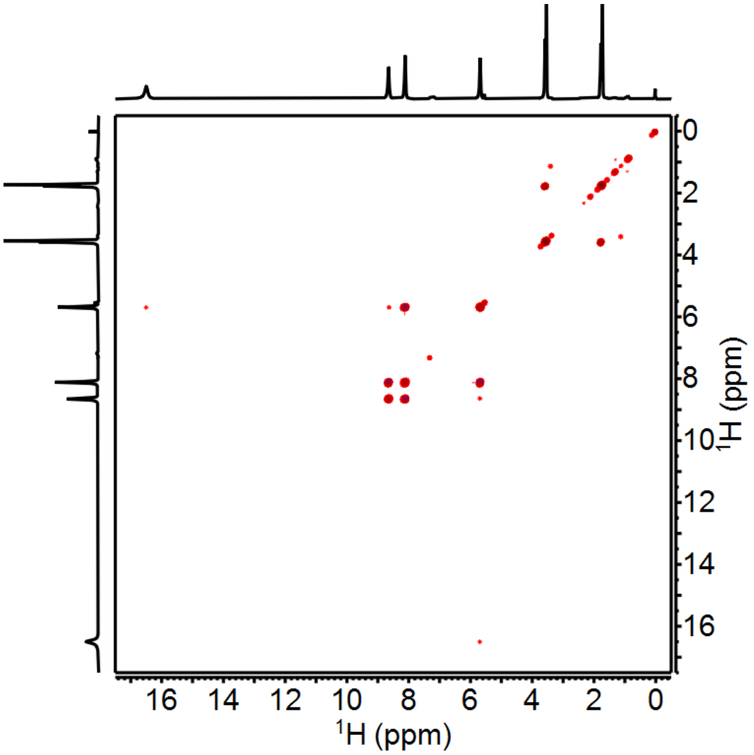

Figure S10. ^1H-^1H-COSY spectrum of [U(PyS)$_4$(THF)] in THF-d$_8$ at 303 K.

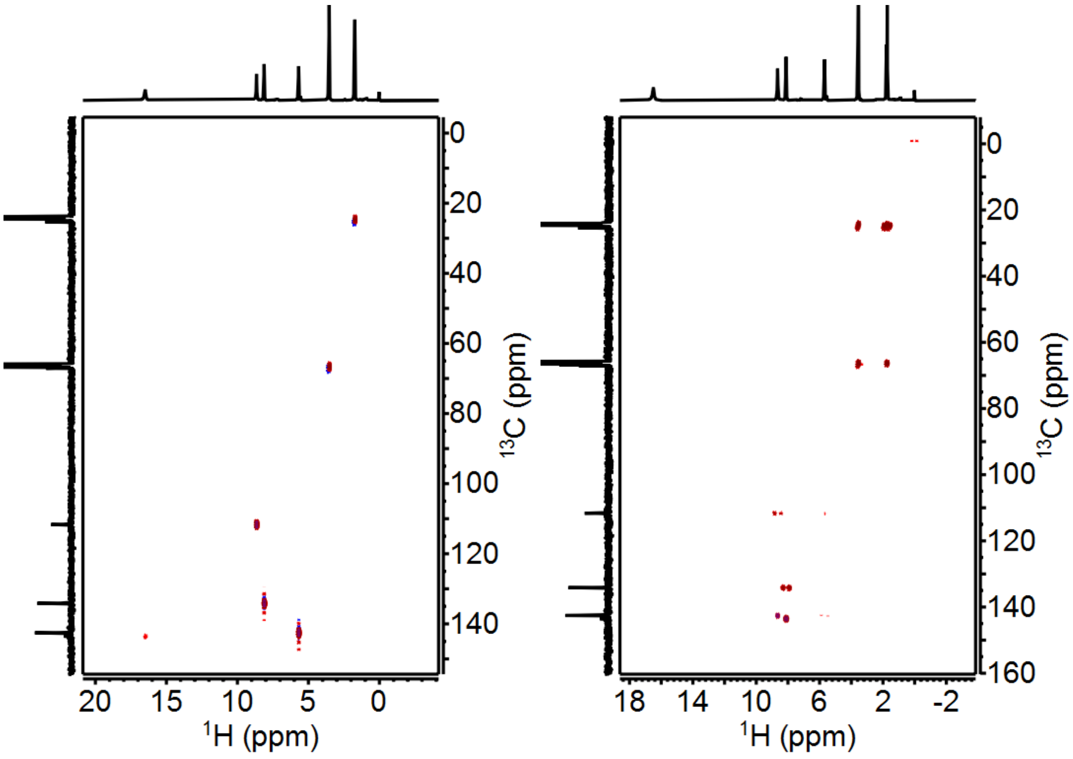

Figure S11. ^1H-^{13}H-HSQC spectrum (left) and ^1H-^{13}H-HMBC spectrum (right) of [U(PyS)$_4$(THF)] in THF-d$_8$ at 303 K.

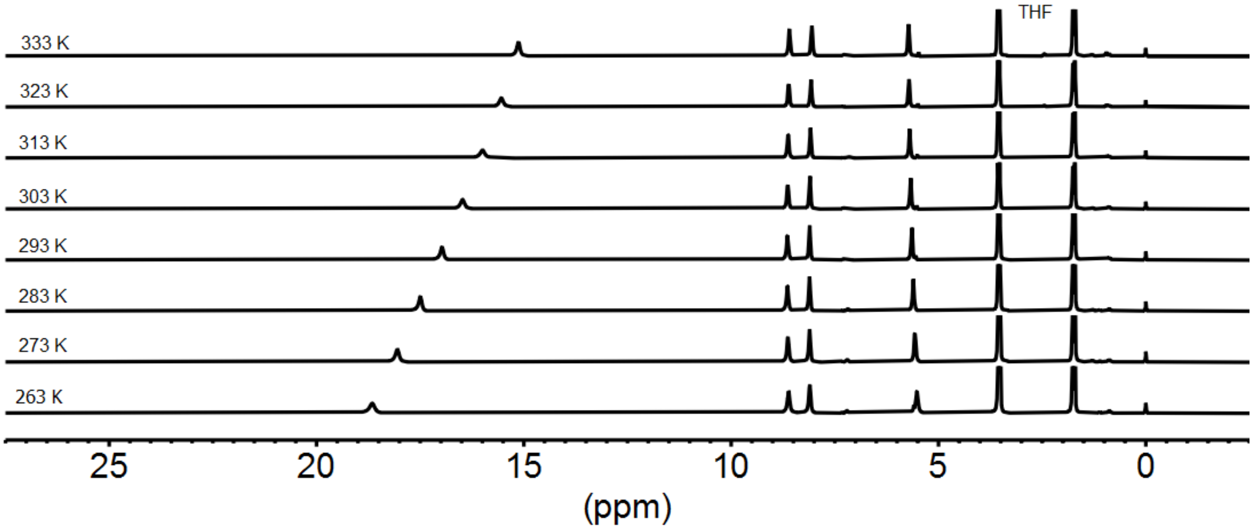

Figure S12. ^{1}H NMR temperature series of [U(PyS)$_4$(THF)] synthesized with KPyS in THF-d$_8$ from 263 K to 333 K. Complex precipitates below 263 K.

Table S2. NMR chemical shifts of [U(PyS)$_4$(THF)] in THF-d$_8$ referenced on TMS at various temperatures.

Temperature (K)	H-3 (ppm)	H-4 (ppm)	H-5 (ppm)	H-6 (ppm)
**333**	8.59	8.05	5.72	15.13
**323**	8.61	8.07	5.71	15.54
**313**	8.62	8.09	5.69	15.98
**303**	8.62	8.09	5.67	16.47
**293**	8.64	8.10	5.64	16.95
**283**	8.64	8.10	5.60	17.48
**273**	8.63	8.10	5.56	18.04
**263**	8.61	8.10	5.52	18.63

4. NMR characterization of [Np(PyS)$_4$(THF)]

^{1}H NMR: (THF-d$_8$, 401.8 MHz, 298 K) δ = 6.94 (4H, H-4), 7.00 (4H, H-5), 9.57 (4H, H-3), 13.65 ppm (4H, H-6), ^{13}C NMR: (THF-d$_8$, 101 MHz, 298 K) δ = 97.13 (C-3), 136.25 (C-4), 152.36 (C-5), 154.85 (C-6), C2 not observed.

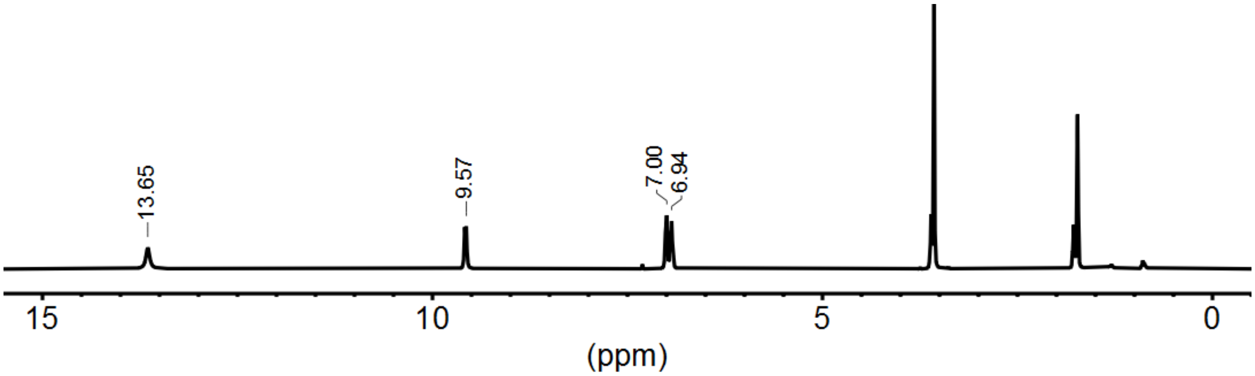

Figure S13. ^{1}H NMR spectrum of [Np(PyS)$_4$(THF)] in THF-d$_8$ at 298 K.

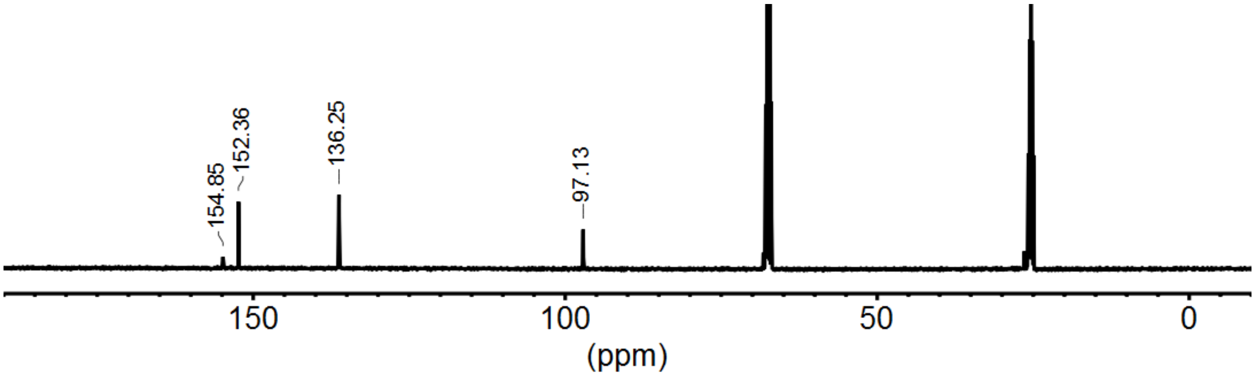

Figure S14. ^{13}C NMR spectrum of [Np(PyS)$_4$(THF)] in THF-d$_8$ at 298 K.

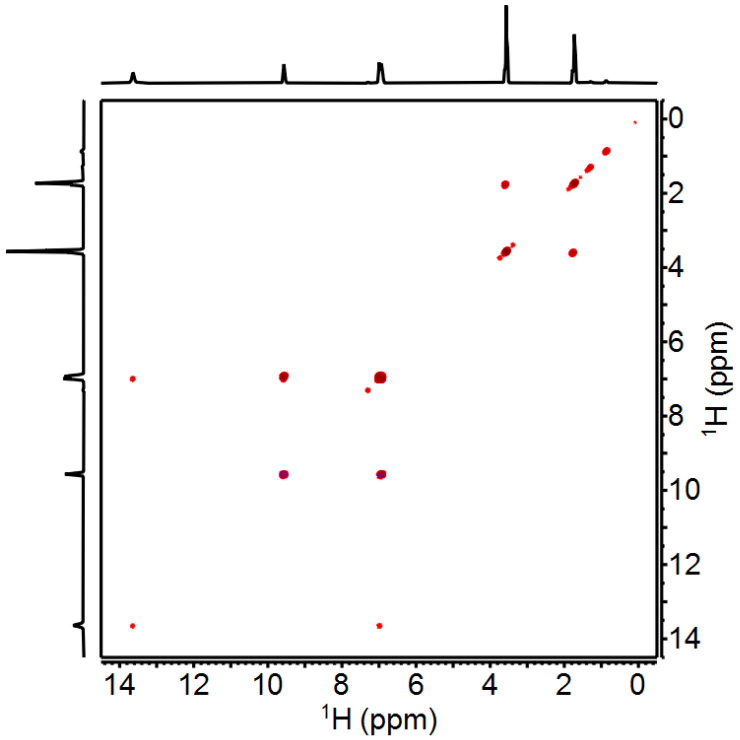

Figure S15. ^{1}H-^{1}H-COSY spectrum of [Np(PyS)$_4$(THF)] in THF-d$_8$ at 298 K.

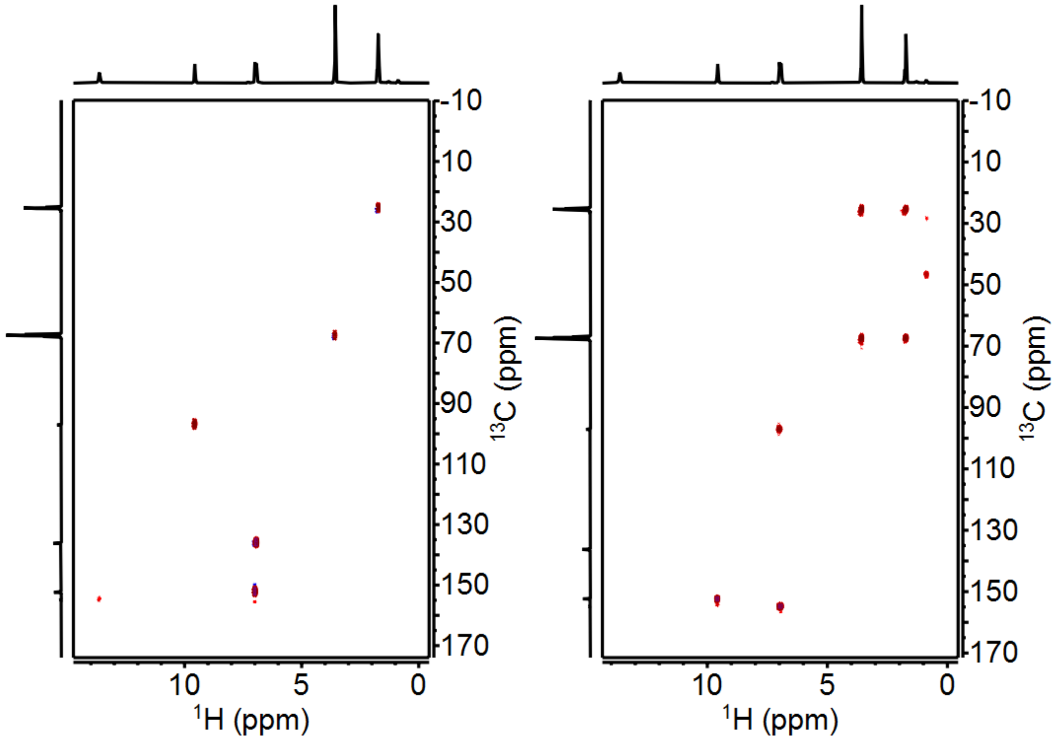

Figure S16. ^{1}H-^{13}H-HSQC spectrum (left) and ^{1}H-^{13}H-HMBC spectrum (right) of [Np(PyS)$_4$(THF)] in THF-d$_8$ at 298 K.

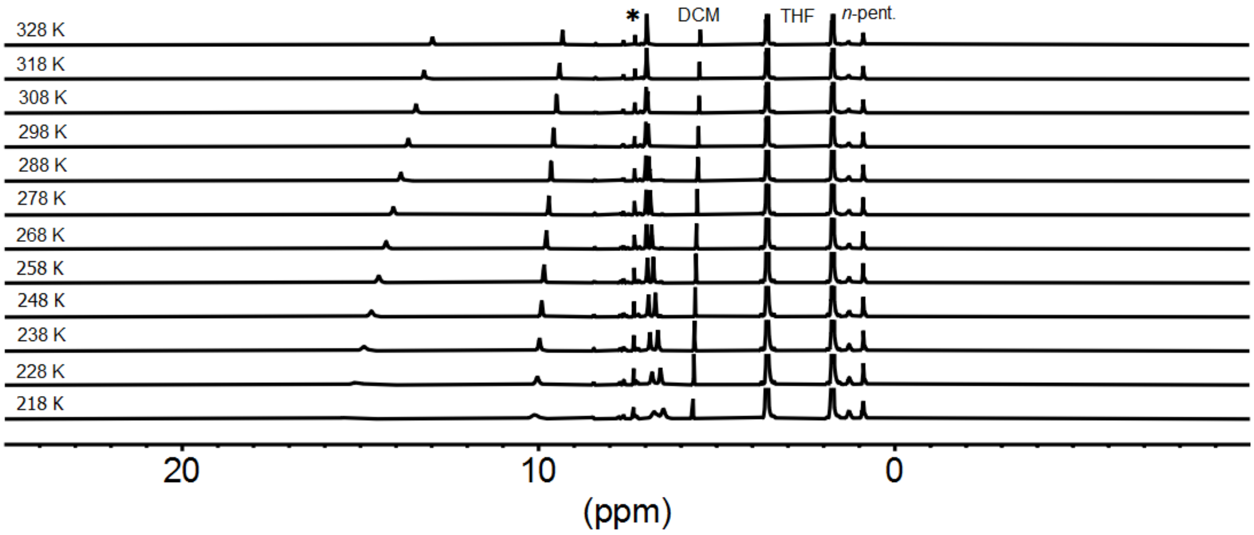

Figure S17. ^{1}H NMR temperature series of [Np(PyS)$_4$(THF)] in THF-d$_8$ from 218 K to 328 K. ∗ marks the signals of free ligand.

Table S3. NMR chemical shifts of [Np(PyS)$_4$(THF)] in THF-d$_8$ referenced on TMS at various temperatures.

Temperature (K)	H-3 (ppm)	H-4 (ppm)	H-5 (ppm)	H-6 (ppm)
**328**	9.32	6.97	6.97	12.98
**318**	9.41	6.95	6.97	13.20
**308**	9.49	6.94	6.98	13.44
**298**	9.57	6.94	7.00	13.65
**288**	9.65	6.90	6.99	13.86
**278**	9.72	6.87	6.99	14.08
**268**	9.78	6.83	6.98	14.28
**258**	9.85	6.78	6.95	14.48
**248**	9.91	6.72	6.92	14.69
**238**	9.97	6.66	6.88	14.90
**228**	10.03	6.57	6.82	15.11
**218**	10.10	6.51	6.75	15.32

5. NMR characterization of $[Pu(PyS)_4]$ / $[Pu(PyS)_4(THF)]$

^{1}H NMR: (DCM-d_2, 399.9 MHz, 223 K) δ = 7.08 (4H, H-5), 7.49 (4H, H-3), 7.56 (4H, H-4), 8.37 ppm (4H, H-6), ^{1}H NMR: (THF-d_8, 401.8 MHz, 298 K) δ = 7.05 (4H, H-5), 7.54 (8H, H-3 & H-4), 8.33 ppm (4H, H-6), ^{13}C NMR: (DCM-d_2, 100.6 MHz, 303 K) δ = 119.48 (C-3), 121.08 (C-5), 137.26 (C-4), 149.49 (C-6), 158.68 (C-2).

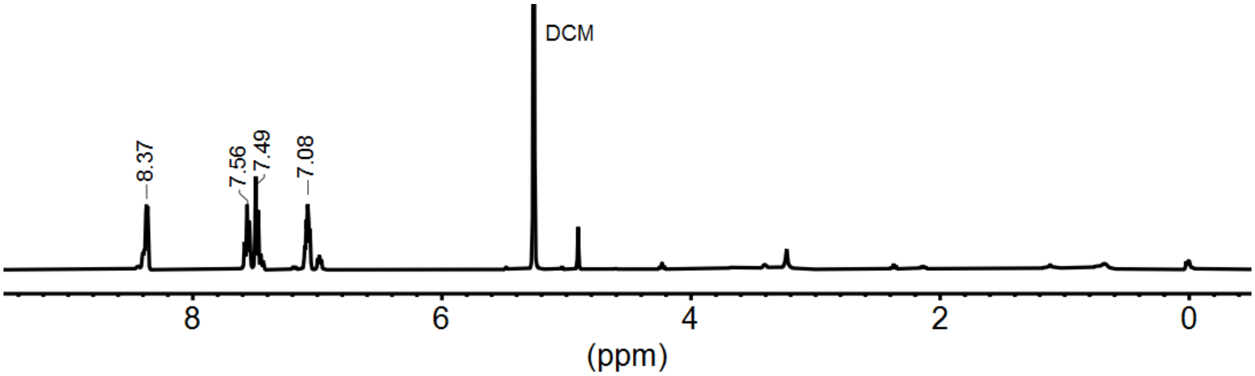

Figure S18. ^{1}H NMR spectrum of $[Pu(PyS)_4]$ in DCM-d_2 at 223 K. Second set of signals (4.90, 6.98, 7.10, 7.45 and 8.40 ppm) was assigned to bis(2pyridylthio)methane formed as an activation product of DCM, mediated by the An.[2]

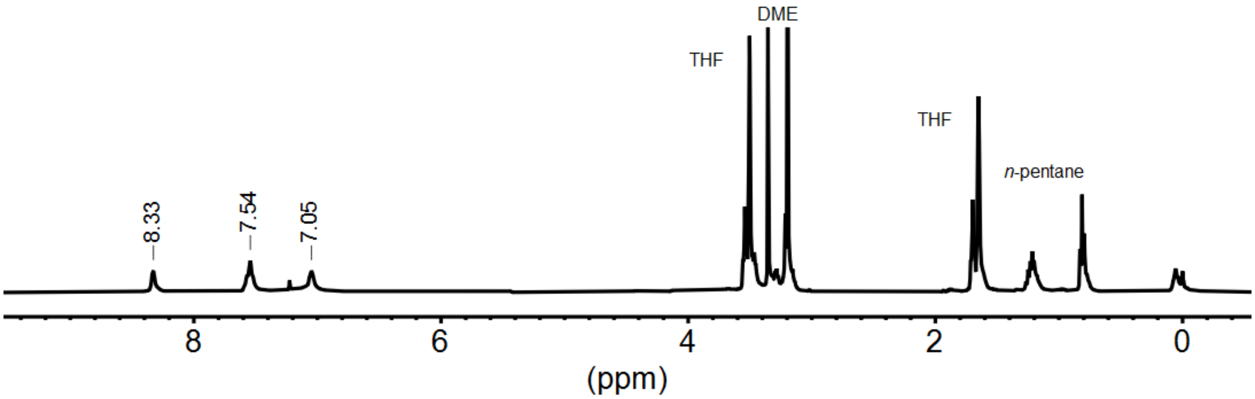

Figure S19. ^{1}H NMR spectrum of $[Pu(PyS)_4]$ in THF-d_8 at 298 K. Shoulders on the THF signals indicate a possible $[Pu(PyS)_4(THF)]$ species in THF solution.

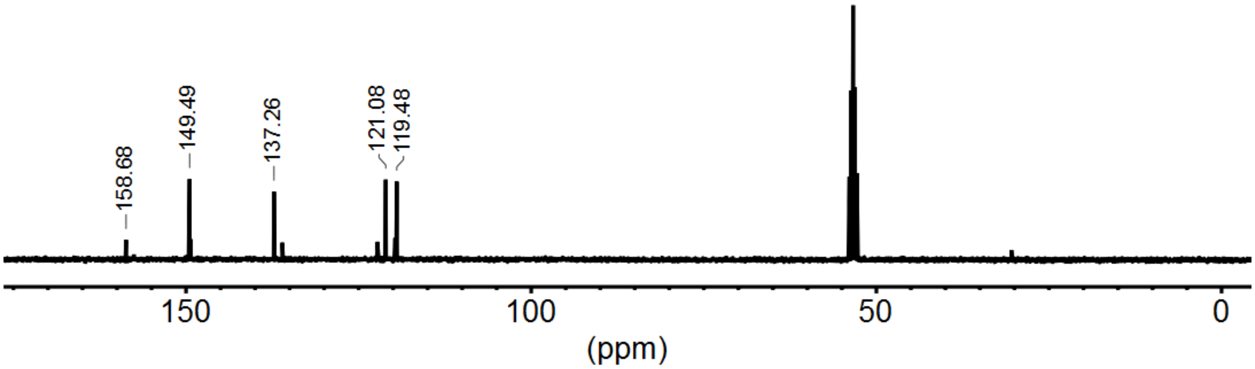

Figure S20. ^{13}C NMR spectrum of $[Pu(PyS)_4]$ in DCM-d_2 at 303 K.

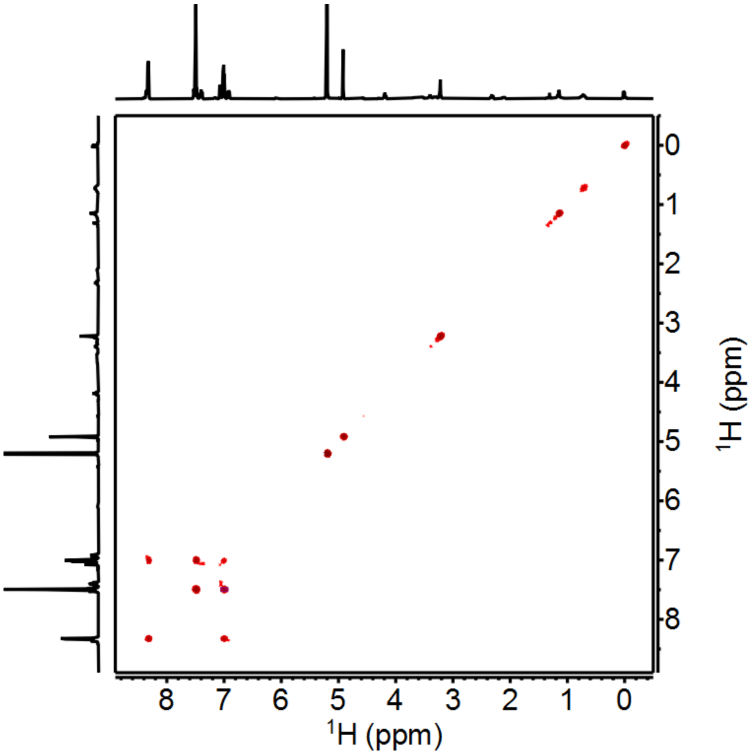

Figure S21. ^{1}H-^{1}H-COSY spectrum of [Pu(PyS)$_4$] in DCM-d$_2$ at 303 K.

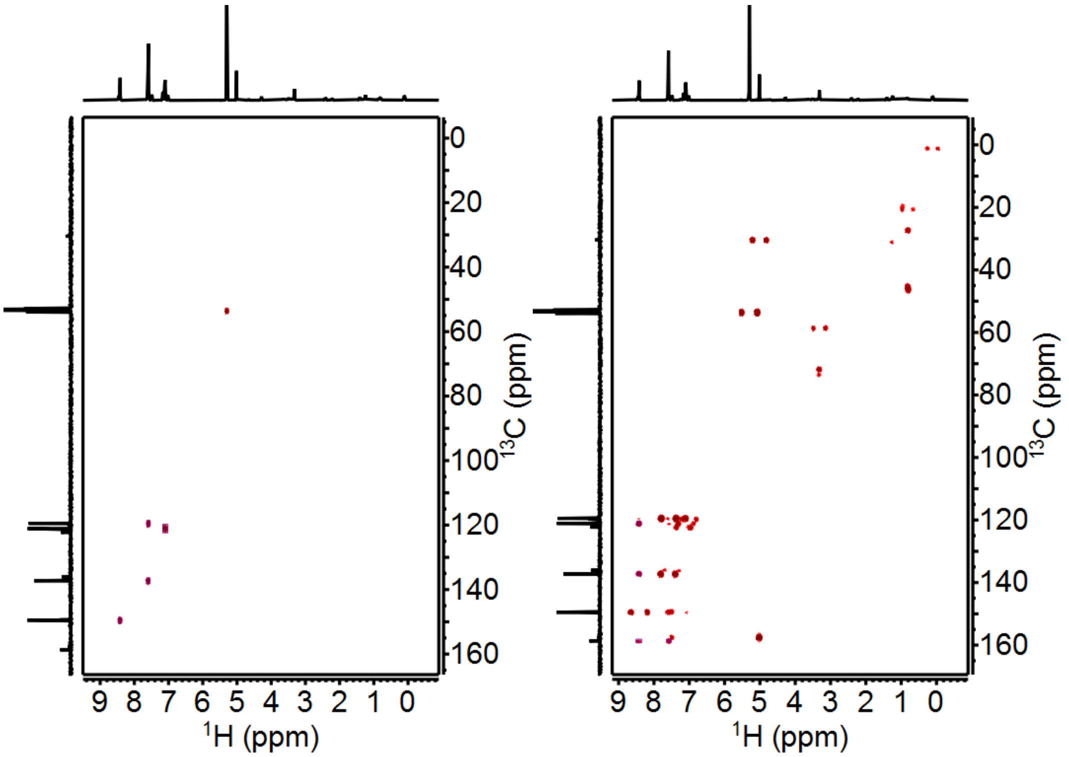

Figure S22. ^{1}H-^{13}H-HSQC spectrum (left) and ^{1}H-^{13}H-HMBC spectrum (right) of [Pu(PyS)$_4$] in DCM-d$_2$ at 303 K.

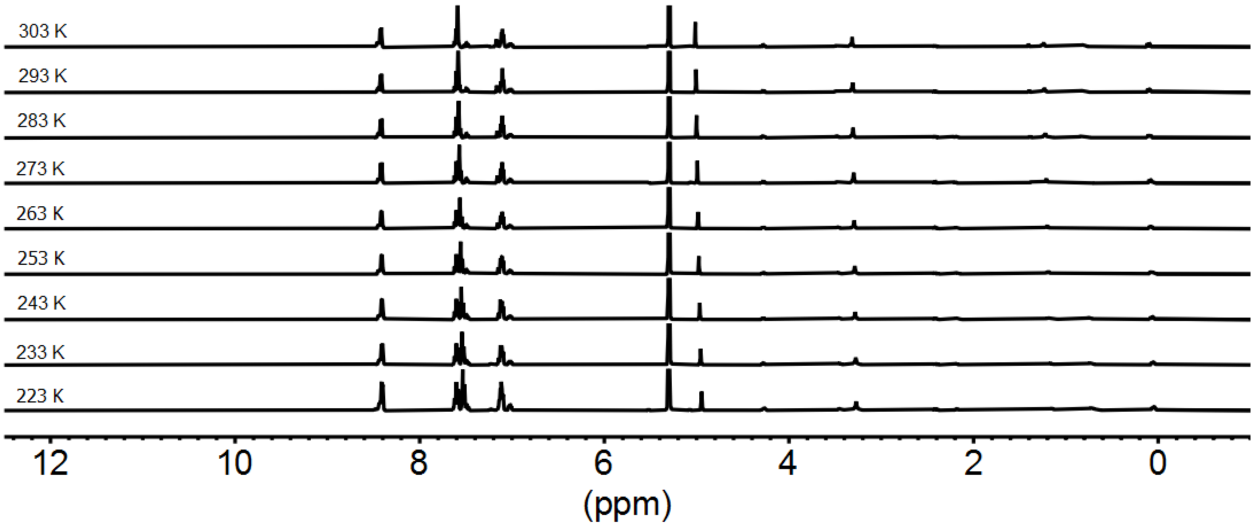

Figure S23. ^{1}H NMR temperature series of [Pu(PyS)$_4$] in DCM-d_2 from 223 K to 303 K.

Table S4. NMR chemical shifts of [Pu(PyS)$_4$] in DCM-d_2 referenced on TMS at various temperatures.

Temperature (K)	H-3 (ppm)	H-4 (ppm)	H-5 (ppm)	H-6 (ppm)
**303**	7.47	7.47	6.99	8.30
**293**	7.47	7.47	6.99	8.31
**283**	7.47	7.50	7.00	8.31
**273**	7.46	7.50	7.01	8.32
**263**	7.46	7.51	7.02	8.32
**253**	7.46	7.52	7.02	8.33
**243**	7.47	7.53	7.03	8.33
**233**	7.48	7.56	7.07	8.36
**232**	7.49	7.56	7.08	8.37

6. NMR characterization of K[Th(PyS)$_5$]

^{1}H NMR: (THF-d_8, 399.9 MHz, 303 K) δ = 6.46 (4H, H-5), 6.73 (4H, H-3), 7.02 (4H, H-4), 8.39 ppm (4H, H-6), ^{13}C NMR: (THF-d_8, 100.6 MHz, 303 K) δ = 115.39 (C-5), 120.37 (C-4), 135.68 (C-3), 146.88 (C-6), 176.72 ppm (C-2).

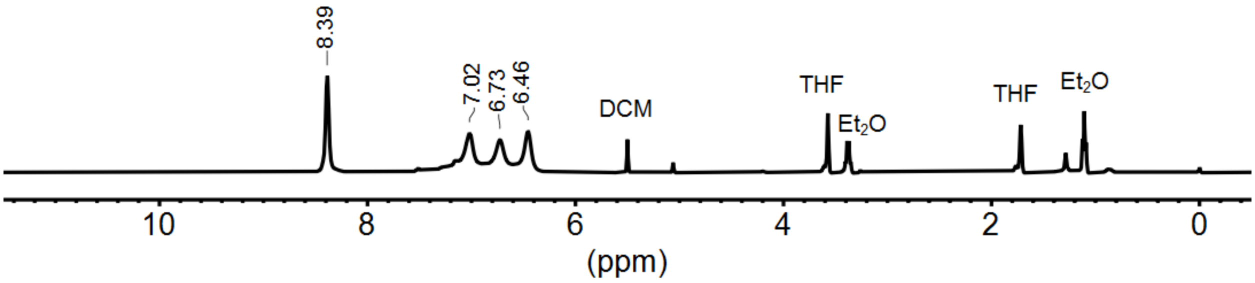

Figure S24. ^{1}H NMR spectrum of K[Th(PyS)$_5$] synthesized with KPyS after centrifugation in THF-d_8 at 303 K.

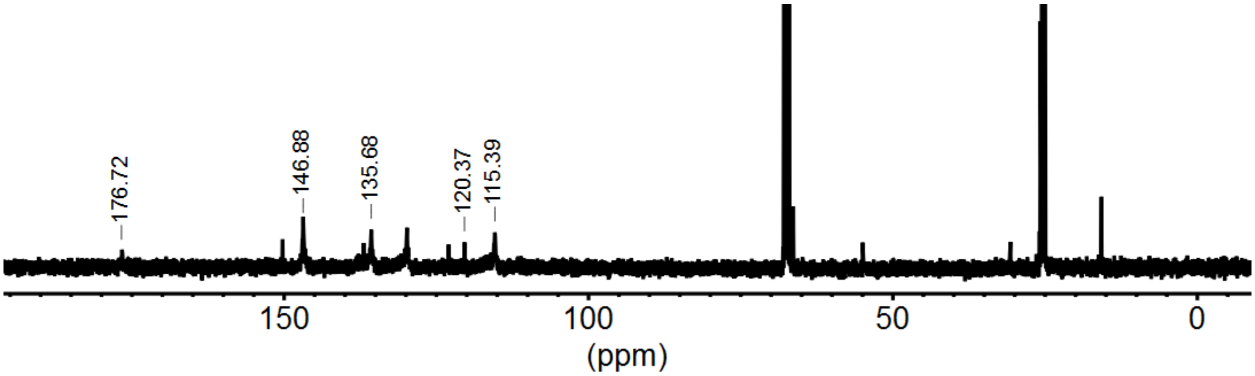

Figure S25. ^{13}C NMR spectrum of K[Th(PyS)$_5$] synthesized with KPyS after centrifugation in THF-d_8 at 303 K.

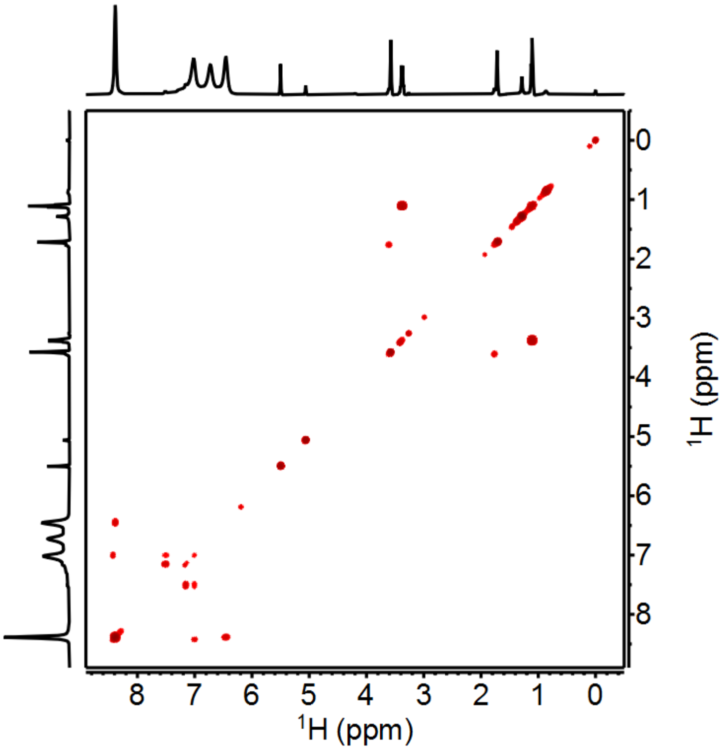

Figure S26. ^{1}H-^{1}H-COSY spectrum of K[Th(PyS)$_5$] in THF-d$_8$ at 303 K.

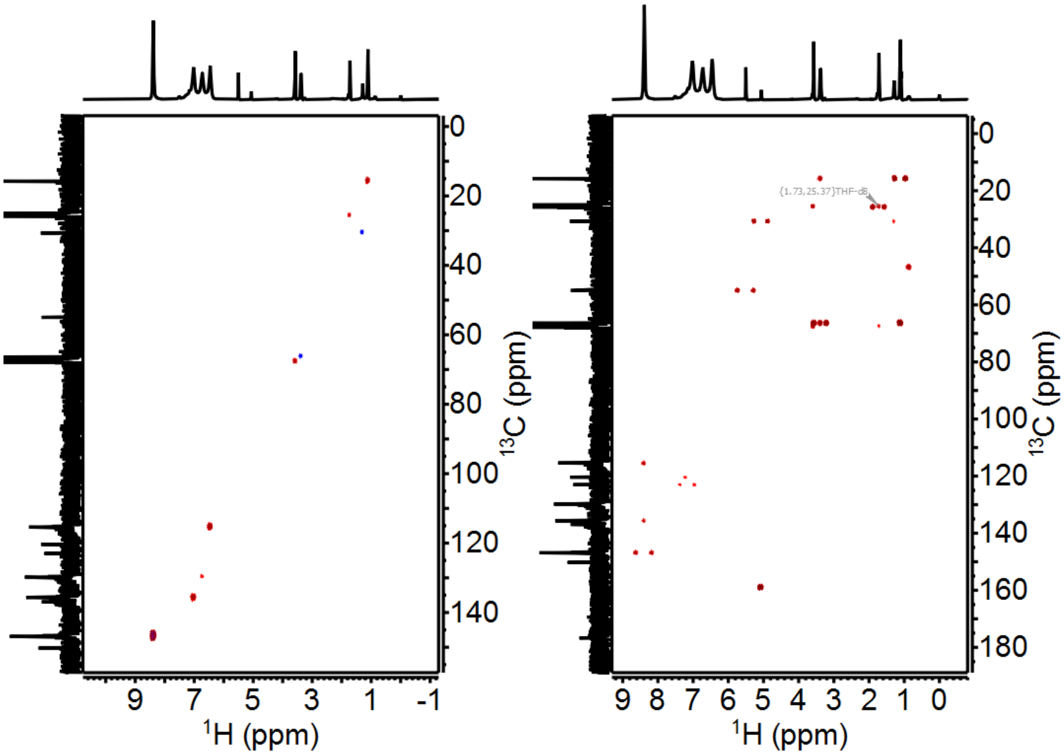

Figure S27. ^{1}H-^{13}H-HSQC spectrum (left) and ^{1}H-^{13}H-HMBC spectrum (right) of K[Th(PyS)$_5$] in THF-d$_8$ at 303 K.

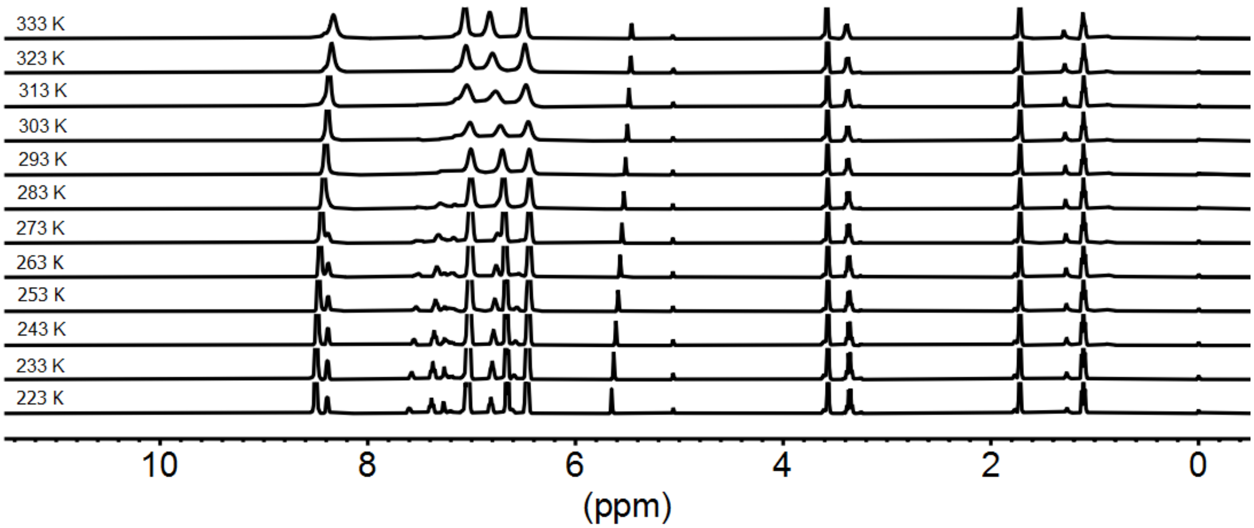

Figure S28. ^{1}H NMR temperature series of K[Th(PyS)$_5$] synthesized with KPyS after centrifugation in THF-d$_8$ from 223 K to 333 K.

Table S5. NMR chemical shifts of K[Th(PyS)$_5$] in THF-d$_8$ referenced on TMS at various temperatures.

Temperature (K)	H-3 (ppm)	H-4 (ppm)	H-5 (ppm)	H-6 (ppm)
**333**	6.83	7.06	6.50	8.33
**323**	6.80	7.05	6.49	8.35
**313**	6.76	7.04	6.48	8.37
**303**	6.73	7.02	6.46	8.39
**293**	6.71	7.01	6.44	8.40
**283**	6.70	7.01	6.44	8.42
**273**	6.68	7.01	6.44	8.45
**263**	6.68	7.01	6.45	8.46
**253**	6.67	7.02	6.45	8.47
**243**	6.67	7.02	6.45	8.49
**233**	6.66	7.03	6.46	8.50
**232**	6.66	7.04	6.47	8.50

7. NMR characterization of K[U(PyS)$_5$]

^{1}H NMR: (THF-d$_8$, 399.9 MHz, 303 K, broad signals) δ = 5.33 (4H), 8.16 (4H), 8.74 (4H), 11.69 ppm (4H).

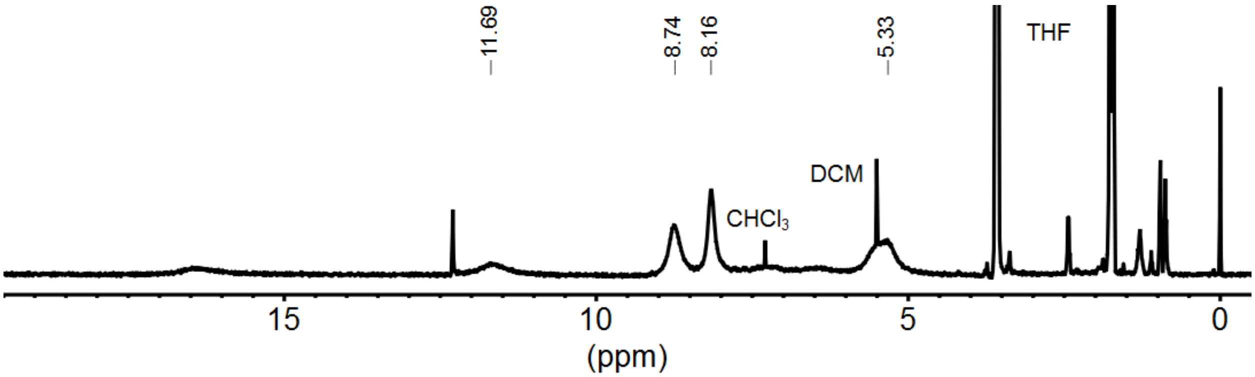

Figure S29. ^{1}H NMR spectrum of K[U(PyS)$_5$] synthesized with KPyS after centrifugation in THF-d$_8$ at 303 K.

For K[U(PyS)$_5$] broad ^{1}H NMR signals are found (δ = 5.33, 8.16, 8.74, 11.69 ppm in THF-d$_8$), hindering an assignment with 2D NMR spectroscopy. Furthermore, small amounts of [U(PyS)$_4$(THF)] (δ = 5.67, 8.09, 8.62, 16.47 ppm) are found in the ^{1}H NMR spectrum, measured in THF-d$_8$. Due to the strong excess of solvent molecules, a conversion may occur, with the hard oxygen donor atom coordinating to U^{IV}. This could be confirmed by an attempt to crystallize K[U(PyS)$_5$] from THF, where triclinic single crystals of [U(PyS)$_4$(THF)] were obtained and measured with SC-XRD (**Figure 2**).

8. NMR characterization of the K[Np(PyS)$_5$] reaction mixture

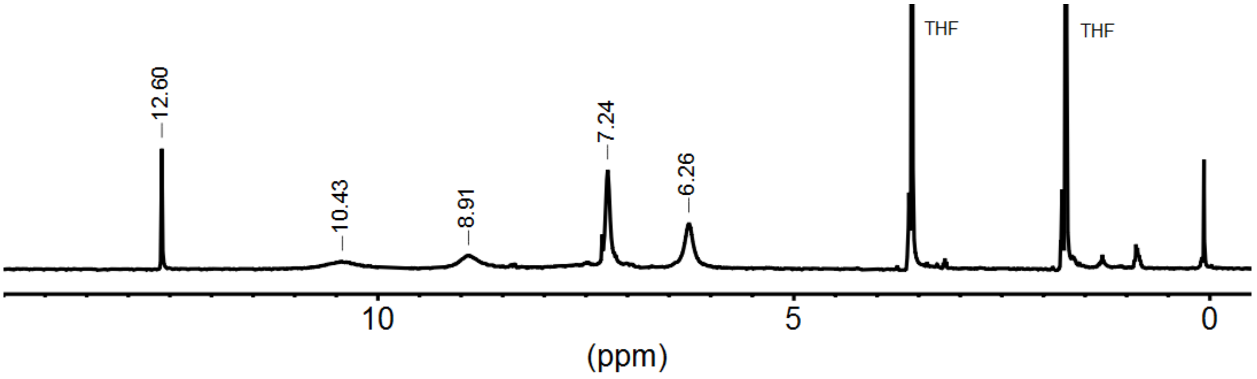

Figure S30. ^{1}H NMR spectrum of the K[Np(PyS)$_5$] reaction mixture in THF-d_8 at 298 K.

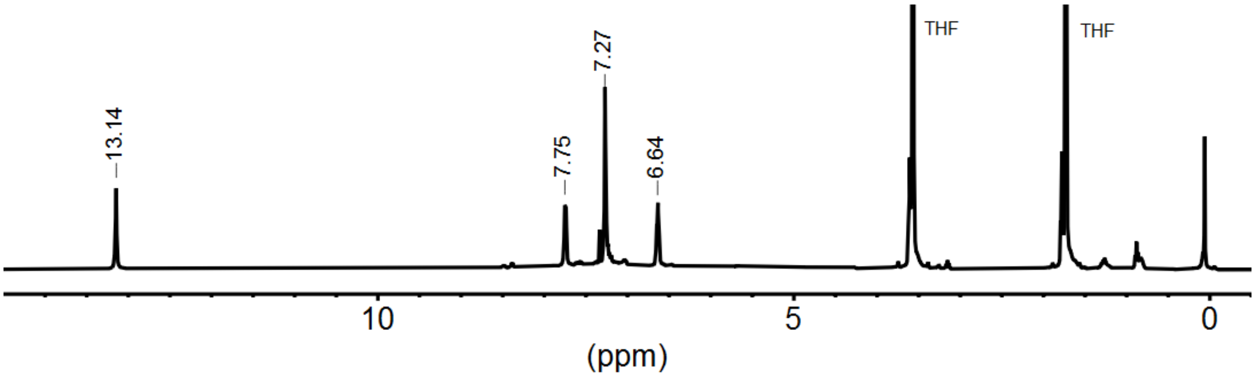

Figure S31. ^{1}H NMR spectrum of the K[Np(PyS)$_5$] reaction mixture in THF-d_8 at 218 K.

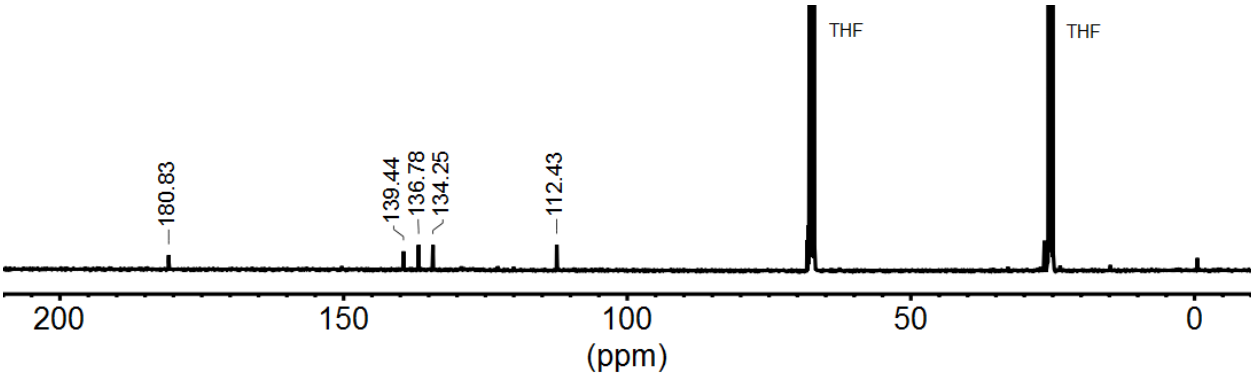

Figure S32. ^{13}C NMR spectrum of the K[Np(PyS)$_5$] reaction mixture in THF-d_8 at 218 K.

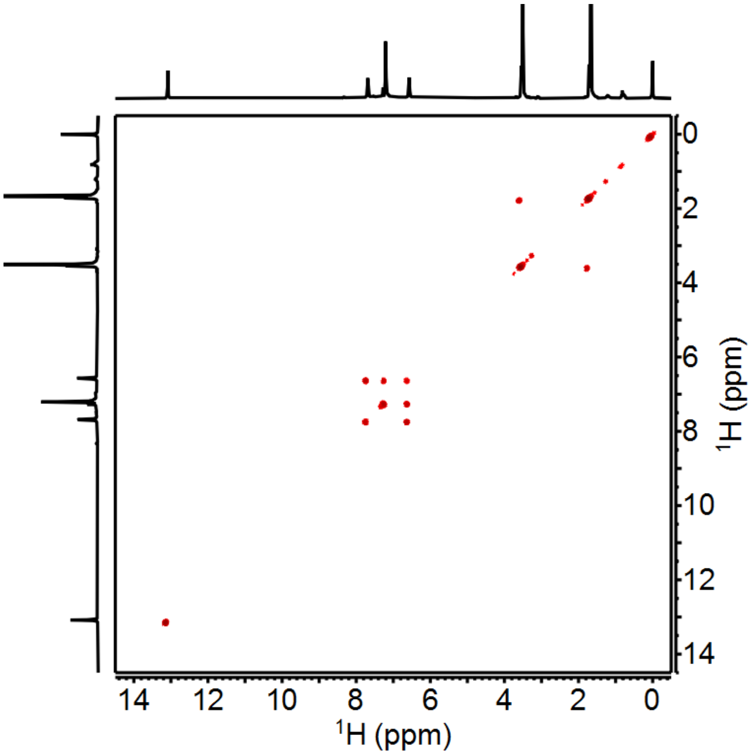

Figure S33. ^{1}H-^{1}H-COSY spectrum of the K[Np(PyS)$_5$] reaction mixture in THF-d$_8$ at 218 K.

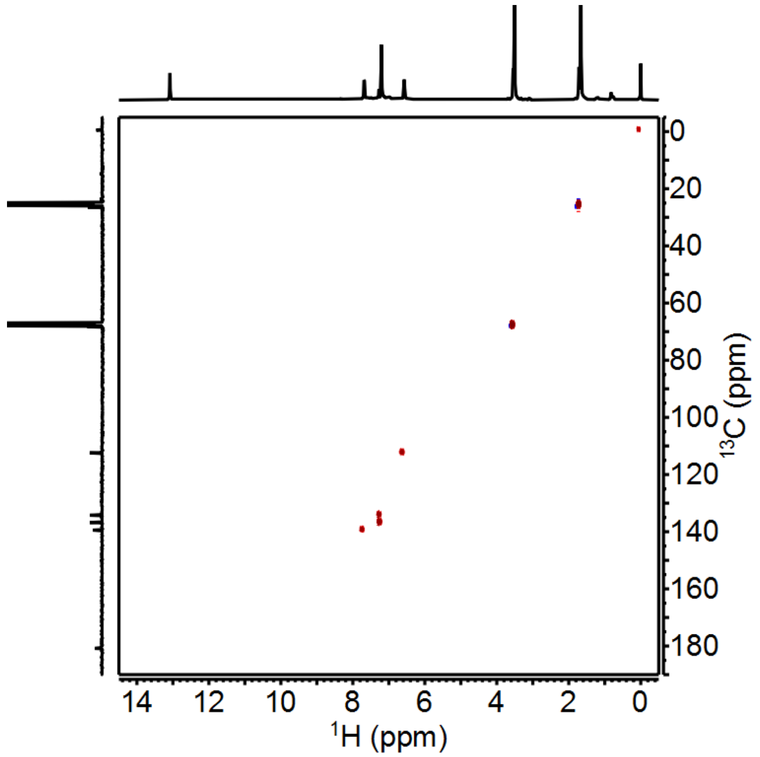

Figure S34. ^{1}H-^{13}H-HSQC spectrum of the K[Np(PyS)$_5$] reaction mixture in THF-d$_8$ at 218 K.

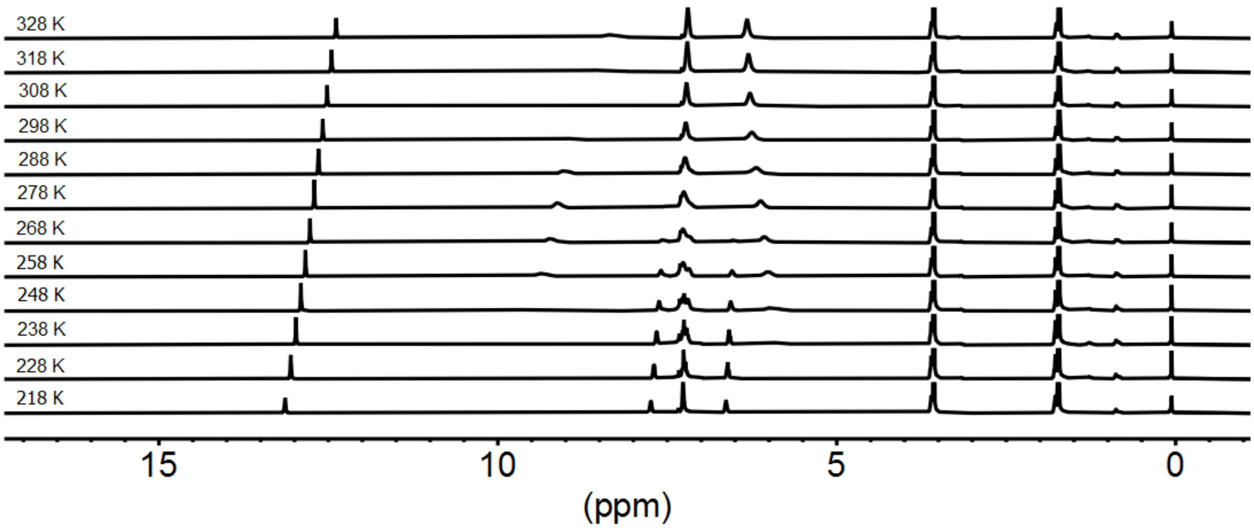

Figure S35. ^{1}H NMR temperature series of the K[Np(PyS)$_5$] reaction mixture in THF-d$_8$ from 218 K to 328 K.

9. NMR characterization of $[An(PyS)_4(THF)_n]$ with possible solvent molecule coordination

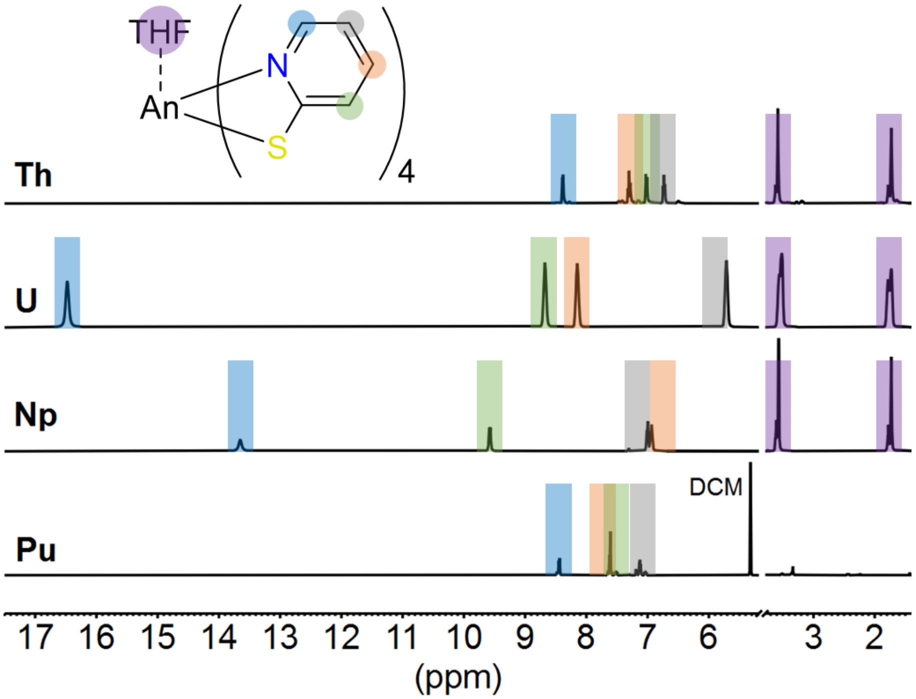

Figure S36. ^{1}H NMR spectra at room temperature in THF-d_8 (**1** – **3**) and DCM-d_2 (**4**).

Assignment of the ligand proton signals with 2D NMR spectroscopy: H-3 (green), H-4 (red), H-5 (grey) and H-6 (blue) Shoulders on the THF signals (purple)[3] of **1** – **3** suggest that solvent molecules contribute to the saturation of the coordination spheres by coordinating to the An centers. No THF signals can be found in the ^{1}H spectrum of a vacuum-dried solid of **4** in DCM-d_2. This indicates a possible formation of $[Pu(PyS)_4]$ over $[Pu(PyS)_4(THF)]$, due to steric hindrance at the small Pu^{IV} ion.[4]

10. NMR characterization of solvent-free complexes [An(PyS)$_4$] in DCM-d$_2$

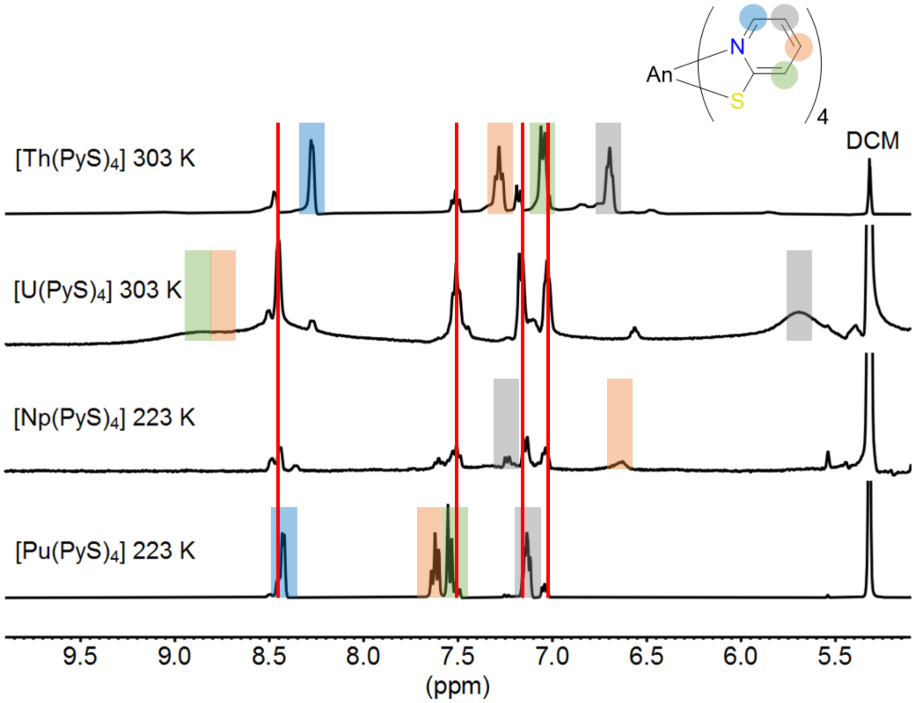

Figure S37. Section of the ^{1}H NMR spectra of the An complexes in DCM-d$_2$ (An: Th, U {303 K}, Np & Pu {223 K}), in an attempt to synthesize them as solvent-free compounds [An(PyS)$_4$].

Broad signals occur for U and Np. Assignment of the ligand proton signals, as far as possible, in comparison to the ^{1}H NMR spectra of the An complexes in THF-d$_8$. In all ^{1}H NMR spectra of the An compounds in DCM-d$_2$ another set of signals was found (4.90, 6.98, 7.10, 7.45 and 8.40 ppm; marked in red) and could be assigned via 2D NMR spectroscopy to bis(2pyridylthio)methane. This species was presumably formed as an activation product of DCM, mediated by the An.[2,5] The by-product could be reproducibly identified with single-crystal X-ray diffraction (**Figure S38**) suggesting an actinide induced C–Cl bond activation.

11. Single-crystal X-ray diffraction

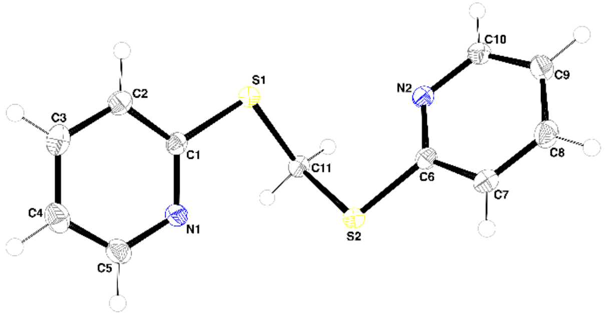

Figure S38. Molecular structure of bis(2-pyridylthio)methane, formed via An mediated activation of dichloromethane. Ellipsoids are shown at 50% probability level.

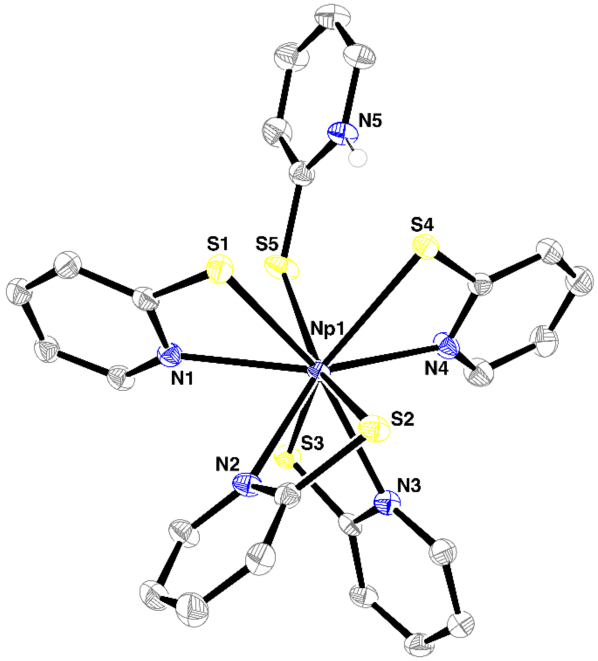

Figure S39. Molecular structure of [Np(PyS)$_4$(PySH)], which was identified in an attempt to crystallize K[Np(PyS)$_5$] from a THF/*n*-pentane solution. Ellipsoids are shown at 50% probability level. Hydrogen atoms are omitted for clarity.

Table S6. Parameters of SC-XRD data collection and structure refinement of [Th(PyS)$_4$(THF)] (**1**, isomer A), [U(PyS)$_4$(THF)] (**2**, isomer B and C) and [Np(PyS)$_4$(THF)] (**3**, isomer C). Experiments were carried out at 100 K with Mo K_α radiation using a Bruker D8 Venture. H-atom parameters were constrained.

	1^A	2^B	2^C	3^C
**Crystal data**				
**Chemical formula**	$C_{24}H_{24}N_4OS_4Th$	$C_{24}H_{24}N_4OS_4U$	$C_{24}H_{24}N_4OS_4U$	$C_{24}H_{24}N_4NpOS_4$
M_r	744.75	750.74	750.74	749.71
**Crystal system, space group**	Orthorhombic, $P2_12_12_1$	Orthorhombic, $P2_12_12_1$	Triclinic, $P\bar{1}$	Triclinic, $P\bar{1}$
***a*, *b*, *c* (Å)**	9.4766 (3), 15.3672 (5), 18.5738 (6)	9.4514 (3), 15.3122 (6), 18.4180 (6)	8.8605 (3), 8.9114 (3), 17.8430 (6)	8.8448 (7), 8.9088 (7), 17.8317 (15)
***α, β, γ* (°)**	90, 90, 90	90, 90, 90	84.0444 (12), 82.1941 (12), 68.8688 (13)	83.722 (3), 82.262 (3), 68.875 (2)
***V* (Å^3)**	2704.88 (15)	2665.48 (16)	1299.67 (8)	1295.90 (18)
Z	4	4	2	2
**μ (mm^{-1})**	5.85	6.43	6.59	4.36
**Crystal size (mm)**	0.12 × 0.09 × 0.07	0.15 × 0.11 × 0.09	0.17 × 0.09 × 0.08	0.09 × 0.07 × 0.03
**Data collection**				
**Absorption correction**	Part of the refinement model (ΔF) Parkin S,Moezzi B & Hope H, (1995) J. Appl. Cryst. 28, 53-56 Cubic fit to sin(Θ)/λ - 24 parameters	Multi-scan *SADABS*	Multi-scan *SADABS* 2016/2: Krause, L., Herbst-Irmer, R., Sheldrick G.M. & Stalke D., J. Appl. Cryst. 48 (2015) 3-10	Part of the refinement model (ΔF) Parkin S,Moezzi B & Hope H, (1995) J. Appl. Cryst. 28, 53-56 Cubic fit to sin(Θ)/λ - 24 parameters
T_{min}, T_{max}	0.527, 0.636	0.656, 0.746	0.621, 0.747	0.592, 0.743
**No. of measured, independent and observed [I > 2σ(I)] reflections**	6212, 6212, 5886	46344, 5993, 5901	187399, 11407, 10348	4562, 4562, 4321
R_{int}	0.035	0.028	0.055	0.059
**(sin(Θ)λ$_{max}$ (Å^{-1})**	0.650	0.649	0.807	0.595

Refinement				
**R[F>2σ(F)], wR(F^2), *S***	0.026, 0.045, 1.06	0.013, 0.029, 1.06	0.025, 0.054, 1.12	0.039, 0.106, 1.15
**No. of reflections**	6212	5993	11407	4562
**No. of parameters**	308	308	307	308
**No. of restraints**	6	0	0	0
	w = 1/[σ2(Fo2) + 3.7516*P*] where $P = (F_o^2 + 2F_c^2)/3$	$w = 1/[\sigma^2(F_o^2)]$ where $P = (F_o^2 + 2F_c^2)/3$	$w = 1/[\sigma^2(F_o^2) + (0.0146P)^2 + 2.9378P]$ where $P = (F_o^2 + 2F_c^2)/3$	$w = 1/[\sigma^2(F_o^2) + (0.0002P)^2 + 31.9367P]$ where $P = (F_o^2 + 2F_c^2)/3$
**$\Delta\rho_{max}$, $\Delta\rho_{min}$ (e Å^{-3})**	0.73, −1.15	0.35, −0.37	1.97, −1.71	2.46, −2.85
**Absolute structure**	Refined as an inversion twin.	Refined as an inversion twin.	–	–
**Absolute structure parameter**	0.022 (7)	0.030 (4)	–	–

Table S7. Parameters of SC-XRD data collection and structure refinement of K[Th(PyS)$_5$]·2.075 DCM (**5**), bis(2-pyridylthio)methane (bptm) and [Np(PyS)$_4$(PySH)]. Experiments were carried out at 100 K with Mo K_α radiation using a Bruker D8 Venture.

	**5**	**bptm**	**[Np(PyS)$_4$(PySH)]**
**Crystal data**			
**Chemical formula**	$C_{25}H_{20}KN_5S_5Th\cdot2.075(CH_2Cl_2)$	$C_{11}H_{10}N_2S_2$	$C_{25}H_{21}N_5NpS_5$
***M*$_r$**	998.01	234.33	788.77
**Crystal system, space group**	Monoclinic, $P2_1/n$	Monoclinic, $P2_1/c$	Monoclinic, $C2/c$
***a*, *b*, *c* (Å)**	19.375 (3), 9.3834 (16), 20.482 (3)	17.7551 (7), 4.1885 (2), 15.0715 (6)	33.6080 (8), 9.6303 (2), 16.7305 (4)
***α*, *β*, *γ* (°)**	91.222 (5)	107.262 (1)	98.1555 (9)
***V* (Å^3)**	3722.8 (11)	1070.34 (8)	5360.1 (2)
Z	4	4	8
**μ (mm^{-1})**	4.72	0.46	4.29
**Crystal size (mm)**	0.26 × 0.10 × 0.06	0.59 × 0.06 × 0.06	0.12 × 0.07 × 0.04

**Data collection**			
**Absorption correction**	Multi-scan *SADABS*	Multi-scan *SADABS*	Part of the refinement model (ΔF) Parkin S,Moezzi B & Hope H, (1995) J. Appl. Cryst. 28, 53-56 Cubic fit to sin(Θ)/λ - 24 parameters
T_{min}, T_{max}	0.564, 0.746	0.664, 0.746	0.576, 0.672
**No. of measured, independent and observed [I > 2σ(I)] reflections**	363432, 8992, 8052	49401, 3127, 2531	7811, 7811, 6117
R_{int}	0.060	0.048	0.063
**(sin(Θ)λ$_{max}$ (Å^{-1})**	0.661	0.705	0.703
**Refinement**			
**R[F>2σ(F)], wR(F^2), *S***	0.036, 0.089, 1.11	0.035, 0.089, 1.08	0.031, 0.053, 1.15
**No. of reflections**	8992	3127	7811
**No. of parameters**	434	136	329
**No. of restraints**	48	0	0
**H-atom treatment**	H-atom parameters constrained	H-atom parameters constrained	H atoms treated by a mixture of independent and constrained refinement
	$w = 1/[\sigma^2(F_o^2) + (0.0336P)^2 + 22.5669P]$ where $P = (F_o^2 + 2F_c^2)/3$	$w = 1/[\sigma^2(F_o^2) + (0.038P)^2 + 0.5635P]$ where $P = (F_o^2 + 2F_c^2)/3$	$w = 1/[\sigma^2(F_o^2) + 40.3832P]$ where $P = (F_o^2 + 2F_c^2)/3$
**Δρ$_{max}$, Δρ$_{min}$ (e Å^{-3})**	1.79, −1.25	0.33, −0.33	1.09, −1.02

12. Quantum chemical calculations

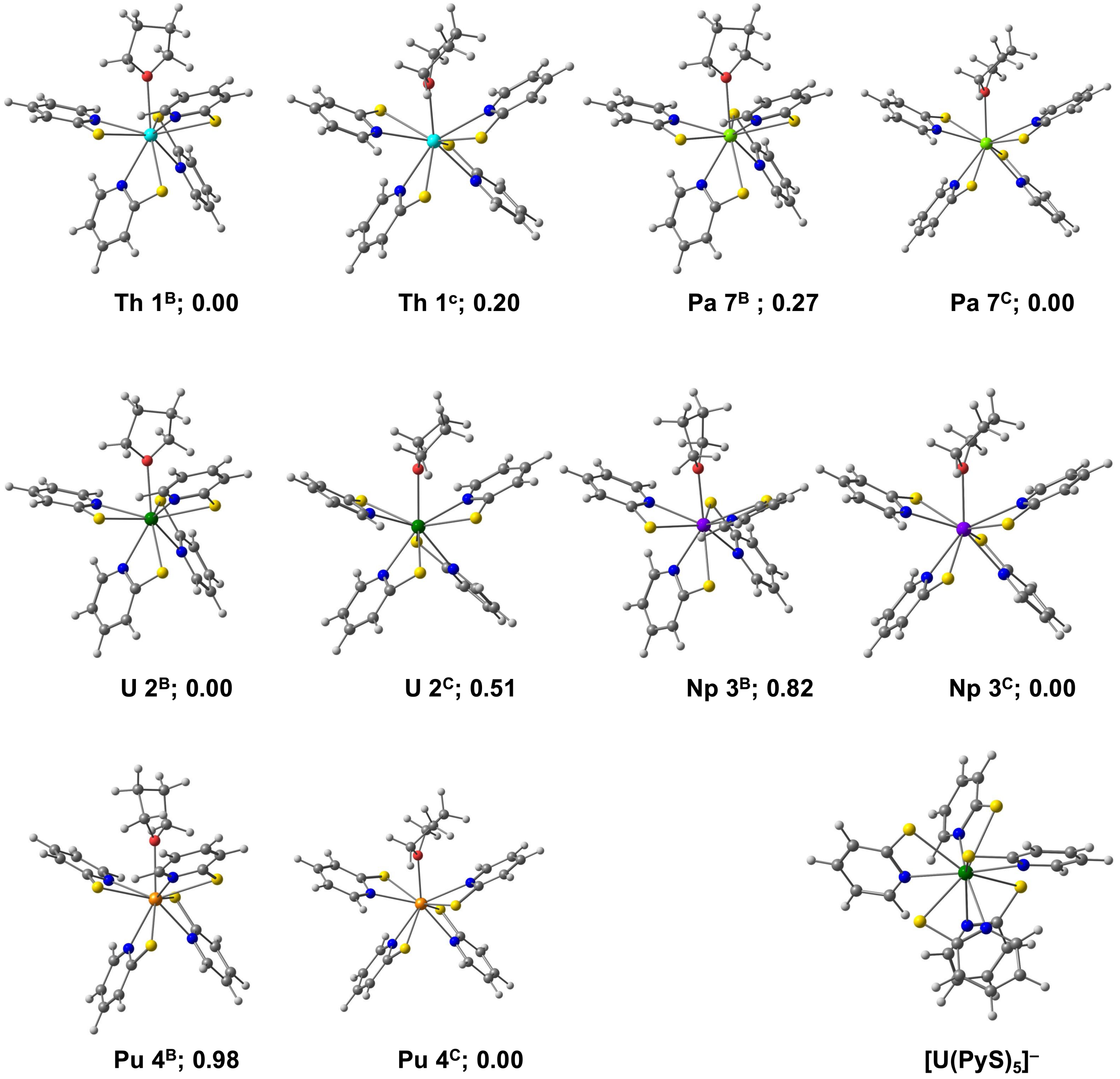

Figure S40. Calculated complex structures of $[An(PyS)_4(THF)]$ (An = Th – Pu) and $[U(PyS)_5]^-$ after geometry optimization. Based on the molecular structures found with SC-XRD for $[An(PyS)_4(THF)]$, an orthorhombic structure was used for isomer B, respectively a triclinic initial structure for isomer C. The Gibbs free energy difference between the two isomers B & C is given in kcal/mol.

Table S8. Atomic coordinates for optimized structure of **Th 1^B**.

PBE0:

final single point energy: -30553.809770770964 a.u.

final Gibbs free energy: -30553.42868537 a.u.

Th	-0.05182904459613	-0.02299357110977	0.05166911940026
N	-1.45145381295894	0.19750238197073	-2.08539463535754
N	-2.46526842745688	0.62461749676854	0.80590540366782
N	1.05448573214872	1.66550030154309	-1.58791373690656
N	1.19151770953626	-2.10989934001892	0.96344994251438
S	0.38386450218730	-1.66126093706269	-2.27788470506790
S	-2.19600188543937	-1.94480800166942	0.39728228772108
S	0.16182606979433	2.81595081337321	0.58712470336808
S	2.80431185354594	-0.10243836371355	0.49548838148434
O	-0.04355702033053	0.09730724488412	2.60593306797587
C	-0.91963684461107	-0.71199965486329	-2.92228775966494
C	-1.41384526052306	-0.84510545868490	-4.22419591738165
C	-2.44607558005958	-0.02972355981933	-4.63550419874370
C	-2.98049623400974	0.91125775722766	-3.75678208253059
C	-2.44791236860599	0.98753627603777	-2.48934982477583
C	-3.12652374012942	-0.54681752973746	0.83094449172880
C	-4.47779657055564	-0.59287306191470	1.19268151495706
C	-5.12058701535562	0.57799261045069	1.53067660152545
C	-4.42088512209818	1.78318151427615	1.50771084930041
C	-3.09441840252423	1.75540788271397	1.13792802486190
C	0.92631072978898	2.85276135935359	-0.97552986582298
C	1.39210246483728	4.02308372128383	-1.58247356731329
C	1.98996306891064	3.93897813076269	-2.82169340284690
C	2.12089112277739	2.69903801657789	-3.44299776486255
C	1.63827704995516	1.58877491998429	-2.78543780706051
C	2.46246544411651	-1.69189669992179	1.10581538466104
C	3.41852895612159	-2.51799757801141	1.70639224527095
C	3.04478032406543	-3.77367367649673	2.13400645574868
C	1.72880384790780	-4.19902301011075	1.96510399416467
C	0.83746413624814	-3.32890034407276	1.37671385153060
C	-0.80478987913871	-0.77310797874248	3.47951311203822
C	-0.29100440697255	-0.50702172334539	4.88385315315101
C	1.10106840006932	0.05741298868823	4.63364807538433
C	0.86974250353088	0.89003886581178	3.39816789625561
H	2.58798624893881	2.59733173553185	-4.41291481077561
H	-2.81888174804035	1.69899924139795	-1.76108901492680
H	1.70835969107724	0.59415267405418	-3.21014916004845
H	-4.89473103549568	2.71947795888482	1.76862423205218

H	-0.97708642876072	-1.58244363835952	-4.88435732932629
H	4.43266768511385	-2.15899616141472	1.81996685050885
H	-2.49425718477705	2.65671180011563	1.09144565473767
H	3.77488045988792	-4.42669214309787	2.59786515515703
H	-3.78849733807341	1.56710734114428	-4.05015681392523
H	1.40355517675874	-5.17992874167161	2.28311439940919
H	-4.99165400353842	-1.54479285423956	1.20202671250357
H	-0.20164842860611	-3.59732688455074	1.22683591725809
H	1.27694614793180	4.96930918371313	-1.07095386252906
H	-0.62311411605560	-1.79816427105332	3.15764496464029
H	-1.86252553119371	-0.54387179386497	3.35503286163851
H	-2.83849252683623	-0.12080081673783	-5.64162123559839
H	-0.91368934499369	0.23695336731959	5.38418131910352
H	-0.28824708707618	-1.41389310512544	5.48719950037105
H	2.35749497333325	4.83556778339372	-3.30717633478467
H	1.81338738287370	-0.74228639220331	4.41765487922379
H	1.48055112766188	0.65057258314123	5.46488837968743
H	-6.16673962496765	0.55859808145475	1.81340654376720
H	0.38721580851333	1.84244999481026	3.63309155168311
H	1.75579619314833	1.06609016194464	2.79285157679727

Table S9. Atomic coordinates for optimized structure of **Th 1^{C}**.

PBE0:

final single point energy: -30553.809974036893 a.u.

final Gibbs free energy: -30553.42836602 a.u.

Th	-0.04722148748935	-0.02011043887996	0.07113451500490
N	-1.07037418167241	-1.29075443517869	-1.91039408899646
N	-1.31457381049190	-2.10633833516873	1.01465180807116
N	0.93176534921896	1.14708048550602	-1.99510317666062
N	1.12044249224977	2.12352571253584	0.95076507046227
S	1.26266152209362	-2.23580510223516	-1.21726128313127
S	-2.82110053691629	0.03210534819795	0.93978766311970
S	-1.39782860773623	2.10375086643091	-1.32359118247978
S	2.77423894976127	0.10258703227540	0.78608486582014
O	0.06190361523498	-0.04352830197854	2.61288464004171
C	-0.20244774457652	-2.28898734002552	-2.15017375762830
C	-0.50119532624114	-3.27457792325968	-3.09620368338410
H	0.20661624442819	-4.07270224828480	-3.27602729598530
C	-1.69615694971975	-3.20102454751070	-3.78011860885168
H	-1.94274623963170	-3.95540767584815	-4.51830237612887
C	-2.58357427890786	-2.15815687945153	-3.52060189860245
H	-3.52721114190801	-2.07444073135905	-4.04190473335406
C	-2.22735920082220	-1.22463043069808	-2.57209540108661
H	-2.86602846059188	-0.38824219356693	-2.31586146637683
C	-2.52020840643773	-1.62227425029750	1.36604679383991
C	-3.44882734043901	-2.44011792176295	2.02031250977738
H	-4.41176265255440	-2.03206823131396	2.29726994529958
C	-3.11488487835758	-3.74891174295497	2.29283218192153
H	-3.82406214366242	-4.39398802137678	2.79845954312826
C	-1.86558392626983	-4.23784857459789	1.91515815162940
H	-1.57389227983357	-5.26068714882508	2.10989717302227
C	-0.99961826620054	-3.37681805528213	1.27698422040481
H	-0.01561020775042	-3.69225499104690	0.94946259364002
C	0.04493136132151	2.11365553264606	-2.28944439959098
C	0.30874567999004	3.02906244114328	-3.31351121381299
H	-0.41307477883065	3.80388288312464	-3.53490482604166
C	1.48737508808844	2.91657394418048	-4.01967580546701
H	1.70684600673982	3.61579803052644	-4.81828812699589
C	2.39331012041602	1.90486571519624	-3.70540493300373
H	3.32453181847813	1.79137527429998	-4.24314079631014
C	2.07284935456541	1.04316046615887	-2.67953417909336
H	2.72899587941772	0.23713561441116	-2.37546698807325
C	2.37012860328335	1.72340442640580	1.25176687064745
C	3.25572605233700	2.59774538637963	1.89358783086920
H	4.25629407020640	2.26027139063720	2.12905807965696

C	2.83224477527766	3.87100027170287	2.20639233514247
H	3.50807653471467	4.55903097327411	2.70103829172521
C	1.53577072943059	4.26998339821186	1.88469948079748
H	1.17424871601923	5.26289247156689	2.11423752042963
C	0.71586144528833	3.35851767270190	1.25707743795570
H	-0.30112569092220	3.60262391624534	0.97287690025887
C	-0.56349374639088	0.85742920661350	3.53524521327620
H	-1.27462146667819	1.46040008062446	2.97555289207577
H	0.21308995718353	1.49958228011133	3.96165386019997
C	-1.19951707108709	-0.04786158626922	4.58051039057861
H	-1.15030803739100	0.40293906299700	5.57084982598028
H	-2.24691462024573	-0.22502917744639	4.33829020598270
C	-0.39236514419863	-1.35824606406742	4.48541949190374
H	0.07593221027555	-1.63271900837779	5.42975388046857
H	-1.03411334159426	-2.18227398362031	4.17402806462328
C	0.65277961822367	-1.06870916041689	3.41975573713805
H	1.58046145326394	-0.67532508279133	3.84436549187490
H	0.89113576504158	-1.91135542721218	2.77436487528719

Table S10. Atomic coordinates for optimized structure of **Pa 7^B^**.

PBE0:

final single point energy: -31385.950023918838 a.u.

final Gibbs free energy: -31385.56940836 a.u.

Pa	-0.07282266299038	-0.02671313600671	0.03084396747258
N	-1.46991915159443	0.23306611753173	-2.05693189988835
N	-2.45855783233068	0.58157068318362	0.80576322796567
N	1.05428498078153	1.61021908505114	-1.61321192046183
N	1.15613265523445	-2.10496811205905	0.93762285748175
S	0.33698042601495	-1.63936844231882	-2.26475307488575
S	-2.16060779342030	-1.96472076081486	0.33197985582503
S	0.08375856829096	2.78651171885682	0.50510136018244
S	2.74096380025383	-0.08809635319247	0.46968284870355
O	-0.05854405011124	0.10286551299499	2.55040661774525
C	-0.94068519051869	-0.66090284089024	-2.91284319535960
C	-1.41607246534439	-0.74813235261108	-4.22515563708728
C	-2.43264325503745	0.09230126759792	-4.62578273856938
C	-2.96601480541806	1.01478084669320	-3.72682214661522
C	-2.44896208748466	1.04964429181976	-2.45096583424962
C	-3.10452186783834	-0.59845307221164	0.82529175974556
C	-4.44330103501983	-0.67170548236083	1.22692219306454
C	-5.09013892004160	0.48272444708301	1.61062134429395
C	-4.40648769045658	1.69734401839863	1.59306735171496
C	-3.09174449635293	1.69605629254434	1.18233240383713
C	0.91007277239166	2.80744572081308	-1.02648113399870
C	1.40416293977024	3.96637254304279	-1.62997672352131
C	2.04723038436237	3.86108891152886	-2.84588363331525
C	2.19550426698994	2.61150664864657	-3.44128552477984
C	1.68444271989898	1.51215495076034	-2.78441312874553
C	2.41425017430575	-1.66509989874471	1.11824221188345
C	3.36021191427155	-2.45652788059397	1.77678729763905
C	2.99374484217668	-3.71195626579629	2.21365542114105
C	1.69419124409865	-4.16468984598115	1.99990457965630
C	0.80894840062153	-3.32126365302279	1.36314305656967
C	-0.79299072442519	-0.78474331819363	3.42999298982557
C	-0.26138240275202	-0.52137475366564	4.82934392845548
C	1.10247816187461	0.10317671013511	4.56557465591069
C	0.82067942177430	0.93487713633629	3.34026277655432
H	2.69814985541269	2.49212577074639	-4.39126443213375
H	-2.81750211593636	1.74723401829562	-1.70838391582937
H	1.76760752148618	0.51070044320208	-3.18987764554000

H	-4.88397238600122	2.62137446499185	1.88876163138553
H	-0.97964191743870	-1.47188717757214	-4.90048232865127
H	4.36176635366801	-2.07472977340047	1.92340218749955
H	-2.50729057387059	2.60741974530453	1.13680585476152
H	3.71663221017058	-4.34222201837764	2.71843994253063
H	-3.76154889690010	1.68938944297483	-4.01171965695220
H	1.37453521178342	-5.14611836891763	2.32220065686017
H	-4.94389891170892	-1.63072416308574	1.23101155618671
H	-0.21946851909684	-3.61125370469478	1.18370273197504
H	1.27478868790378	4.92044683022480	-1.13669882514071
H	-0.60225662191467	-1.80351013192512	3.09524397750463
H	-1.85587203551511	-0.56927867826110	3.32556701396718
H	-2.81372606572751	0.03513113936626	-5.63870725336231
H	-0.90371376685236	0.18758653636954	5.35477623322645
H	-0.21032728688786	-1.43751219230292	5.41627739706632
H	2.43633434456928	4.74857470736620	-3.33121247039541
H	1.84487967428523	-0.66349425287233	4.33225684843914
H	1.46765662474703	0.70462077163054	5.39726280885126
H	-6.12666569922293	0.44343415046419	1.92496632203459
H	0.29355276054311	1.86038177515053	3.58722176110380
H	1.69093910752869	1.15824682776825	2.72838871542199

Table S11. Atomic coordinates for optimized structure of **Pa 7**^{C}.

PBE0:

final single point energy: -31385.951164215450 a.u.

final Gibbs free energy: -31385.56983159 a.u.

Pa	-0.04835875857419	-0.02016891436882	0.06627791358185
N	-1.09579366378720	-1.25989452132141	-1.88484605981967
N	-1.27992324675595	-2.09856609518388	0.99454241079248
N	0.95756804413796	1.11711359334462	-1.96894558331733
N	1.08003791514975	2.10893046218606	0.94102743347298
S	1.23766174738247	-2.20052299796884	-1.21706691924410
S	-2.78752308940313	0.02829350974892	0.90972193370664
S	-1.37041162413596	2.07371845278180	-1.31725588416474
S	2.73464381547673	0.09947603311159	0.76969276965099
O	0.05664647530144	-0.04340345487318	2.58198871464928
C	-0.23151080401492	-2.25760258085433	-2.14082201024031
C	-0.53960635404596	-3.23784415051873	-3.08922914321813
H	0.16593108227274	-4.03560626094977	-3.27950917887772
C	-1.73985899263554	-3.15756511610830	-3.76327767389268
H	-1.99417303694265	-3.90612608943528	-4.50474326414355
C	-2.62235343653414	-2.11390085475775	-3.48956075228345
H	-3.56986653387080	-2.02436925949005	-4.00287971777583
C	-2.25773556773285	-1.18750094096914	-2.53719837632562
H	-2.89337430796887	-0.35264167548110	-2.26987410669533
C	-2.48683609659757	-1.62112519470917	1.35113676389260
C	-3.40649017578569	-2.43918326274438	2.01645325470138
H	-4.36948181957421	-2.03431605792975	2.29799396160566
C	-3.06440353711288	-3.74591024020852	2.29079168606493
H	-3.76697869654521	-4.39335198201161	2.80252870505533
C	-1.81517610011179	-4.22910679984345	1.90630502474230
H	-1.51706640284570	-5.24992276342963	2.10210393684852
C	-0.95646950191060	-3.36596303421545	1.26028092992678
H	0.02727430221336	-3.67763342406697	0.92865567230181
C	0.07468147765506	2.08309261306802	-2.27806290937330
C	0.34601336772683	2.99073077796864	-3.30680663615572
H	-0.37338252601168	3.76497556808901	-3.53799297341023
C	1.52862475837942	2.87009247836287	-4.00520992681241
H	1.75490028973381	3.56224297220064	-4.80806415429605
C	2.42954448382416	1.85801043572807	-3.67760195144422
H	3.36354131977009	1.73772638457064	-4.20908137777254
C	2.10179103857571	1.00497255663876	-2.64669795440670
H	2.75448726655748	0.20030563095353	-2.33279402390993

C	2.33069275547374	1.71531363841729	1.24792110111129
C	3.20691335494250	2.58909732391294	1.90245160195302
H	4.20752624138656	2.25502528504637	2.14268707025406
C	2.77439952816307	3.85855459027995	2.21932420721915
H	3.44280540522430	4.54790345461082	2.72210591912530
C	1.47787620149193	4.25163204525478	1.89053958920348
H	1.10911527474080	5.24121283538912	2.12301751682716
C	0.66640675333452	3.33966994525160	1.25243487482555
H	-0.34996683655605	3.58027639802118	0.96375964075884
C	-0.55932509055194	0.86270600792604	3.50513026403042
H	-1.27334765967380	1.46508913629538	2.94888232126953
H	0.22173647011825	1.50463884823445	3.92392695607515
C	-1.18920123050001	-0.03751896159190	4.55856987687910
H	-1.13528929122274	0.41853116821148	5.54629203451391
H	-2.23777478978618	-0.21669722280256	4.32284539406990
C	-0.38141367596515	-1.34774636236042	4.46565135608567
H	0.09465550920943	-1.61555977430800	5.40804847154857
H	-1.02441778605484	-2.17461349788569	4.16455691858891
C	0.65489529409320	-1.06286980357086	3.39026625699549
H	1.58449898413778	-0.66454755296468	3.80626906428080
H	0.89180292373508	-1.90836842568102	2.74858516197069

Table S12. Atomic coordinates for optimized structure of **U 2^{B}**.

PBE0:

final single point energy: -32236.912020576667 a.u.

final Gibbs free energy: -32236.53104414 a.u.

U	-0.06081610193224	-0.00806772271675	0.04463100980283
N	-1.43922127532963	0.19350627474982	-2.03472111960150
N	-2.42997690315718	0.63083999354928	0.79016527499369
N	1.02299192342272	1.63523971724707	-1.58266696792968
N	1.15518742571022	-2.05479029397724	0.96921408823952
S	0.39281151579957	-1.65024434526405	-2.18795027464691
S	-2.15065156679484	-1.91550965752576	0.34020946970953
S	0.17410127373197	2.76389317262340	0.60912628187313
S	2.73459915834581	-0.05418446268997	0.43912988069947
O	-0.06736541381452	0.11402929390177	2.55278964396296
C	-0.90042190183100	-0.71612004631967	-2.86637008653139
C	-1.37823574671274	-0.85023244418088	-4.17345310785049
C	-2.40758890275673	-0.03553111587093	-4.59420403381685
C	-2.94953407437985	0.90634848648674	-3.72114676753772
C	-2.42926817929537	0.98589640642852	-2.44847869030023
C	-3.09539477594591	-0.53766026977544	0.79600824776109
C	-4.44676240569942	-0.58865187815863	1.15425309646487
C	-5.08642425167086	0.57785852406294	1.51421949290447
C	-4.38278652123655	1.78062740102547	1.51279790428870
C	-3.05593593215822	1.75676673860388	1.14119804321010
C	0.91239675859104	2.81998818296540	-0.96379061497728
C	1.37948428137701	3.99045782815343	-1.56831313264896
C	1.95988625573775	3.90904106446758	-2.81642426472840
C	2.07388494375310	2.67145458717618	-3.44484429361263
C	1.59218093420455	1.56055758577523	-2.78636649970096
C	2.42109859768052	-1.62475335297480	1.10765529121097
C	3.38068220867023	-2.41768792608119	1.74396347623694
C	3.01495594938326	-3.66308018443002	2.20910106221432
C	1.70477557240452	-4.10472559635701	2.04249315095783
C	0.80913357087271	-3.26161304664202	1.41959355796712
C	-0.85734881207949	-0.73993306466617	3.41609954330090
C	-0.26025906768760	-0.60272589490210	4.80871689642697
C	1.13410366547226	-0.05385161913050	4.53446806892131
C	0.87376916353206	0.85445732372424	3.35990353817874
H	2.52834913579277	2.57062348546798	-4.42085651832642
H	-2.80515244205671	1.69999875884528	-1.72577101878161
H	1.65370178576256	0.56789003267280	-3.21654589944801
H	-4.85353414726702	2.71348842984395	1.79115024748580

H	-0.93410750835117	-1.58749212016704	-4.82877851635472
H	4.39008686996605	-2.04444158843177	1.85408732110914
H	-2.45418394009763	2.65748567691798	1.11037114655850
H	3.74668103275380	-4.29437469788575	2.69968752130221
H	-3.75490396578016	1.56166156729736	-4.02285689364462
H	1.38560488764227	-5.07824379725323	2.38837880762103
H	-4.96249298383556	-1.53962236791362	1.14637647145255
H	-0.22621054439766	-3.54483348861300	1.27069259692193
H	1.27910558286299	4.93413007669374	-1.04897993585009
H	-0.78354667533656	-1.75297380672043	3.02445024896107
H	-1.89630018446426	-0.41529713418609	3.36143278766973
H	-2.79151846917043	-0.12771853930994	-5.60346721089147
H	-0.83430266285639	0.11098981684314	5.40214144840907
H	-0.25164823703164	-1.55614604793949	5.33539941559590
H	2.32750398640919	4.80542587404347	-3.30218021011467
H	1.81868164160502	-0.85304441940610	4.24095932324375
H	1.56022024837188	0.47939990259872	5.38355453838111
H	-6.13233957205805	0.55651860396021	1.79768526368789
H	0.40508222748575	1.79359121415171	3.66743086892057
H	1.74077136484390	1.06231180621224	2.73860625564884

Table S13. Atomic coordinates for optimized structure of **U 2**^{C}.

PBE0:

final single point energy: -32236.912119535449 a.u.

final Gibbs free energy: -32236.53022580 a.u.

U	0.05404734824069	0.04671539606658	0.08306285354319
N	1.09997250348240	1.24446222216630	-1.86490195243574
N	1.22296379999947	2.12805055736497	0.99924368038230
N	-0.92348843268839	-1.10440994527858	-1.92248898147676
N	-1.10780827081450	-2.04911614134709	0.93381853695907
S	-1.23788182818995	2.19088712241660	-1.24459900403795
S	2.80377423443829	0.06562594746085	0.86936758004053
S	1.39356393954431	-2.05781265740848	-1.25033474798486
S	-2.69415454729534	0.00862861594196	0.79131459691762
O	0.00086420419335	0.01698134060587	2.57838777444228
C	0.23659028223846	2.23395887004046	-2.15444752653678
C	0.55295417656579	3.18927629626927	-3.12605719683630
H	-0.15137945102767	3.98116889054166	-3.34337564800769
C	1.75894397794041	3.09264356065286	-3.78684833564150
H	2.01966336961199	3.82220721685198	-4.54491512834122
C	2.63959108961523	2.05685436527934	-3.47862388938002
H	3.59158379152576	1.95449760033490	-3.98113944487501
C	2.26750331305654	1.15569563106369	-2.50528975245924
H	2.90122798960349	0.32939706618428	-2.20970750629782
C	2.45714904214912	1.69880231523422	1.32154248534870
C	3.36130082418703	2.55321874415360	1.96367195504553
H	4.34788188962586	2.18781931368215	2.21600296588688
C	2.97225542595582	3.84232793612856	2.25456807190890
H	3.66104578497498	4.51693216689642	2.74974605648392
C	1.69251190567985	4.27423208189307	1.90861944891774
H	1.35739686504330	5.28048784186538	2.11925837741320
C	0.85346693007456	3.37990859285783	1.28077328700203
H	-0.15002942470529	3.65268085828660	0.97602044806325
C	-0.03801903966533	-2.06987236190914	-2.22522950942416
C	-0.30157863884073	-2.97738362924850	-3.25657900902764
H	0.42020955245925	-3.75069128820839	-3.48335012576717
C	-1.47961881619306	-2.85767174928837	-3.96200489307054
H	-1.70016788933595	-3.54968561183984	-4.76658585936449
C	-2.38412917428932	-1.84664562685839	-3.63944087564130
H	-3.31504388532427	-1.72763573496186	-4.17653659846656
C	-2.06390136038566	-0.99273358449114	-2.60726878973655
H	-2.71809153676276	-0.18758591857286	-2.29842771834782

C	-2.34693658691241	-1.62662028231874	1.24523260785609
C	-3.25138214534377	-2.48461879730993	1.88067042041167
H	-4.24243139630502	-2.12558208608965	2.12439838080387
C	-2.85676633172742	-3.77201238276394	2.17468474983919
H	-3.54639377393621	-4.45100019690488	2.66268546524268
C	-1.57124206918530	-4.19583213728025	1.84229258049354
H	-1.23204895701673	-5.19978252623385	2.05757667863917
C	-0.73070078648986	-3.29615084808733	1.22380742265526
H	0.27906755970960	-3.56071931514755	0.93337194456197
C	0.65316962474604	-0.91733983238885	3.44737244899987
H	1.35239275937531	-1.49461691403642	2.84724061746027
H	-0.10991503524635	-1.57925763228612	3.86838117299501
C	1.31070415623006	-0.05035363092885	4.51065964692551
H	2.34545508994147	0.15638437038672	4.23903452146931
H	1.30502669148372	-0.54656177584400	5.48031812414316
C	0.47890790358551	1.24803272834843	4.50304135205380
H	0.02489908156820	1.46138206485194	5.46992776485054
H	1.09935253323277	2.10000526392798	4.22559683538558
C	-0.58337778431169	0.99712080593296	3.44399095395829
H	-1.49776560334240	0.57287072572647	3.86821957710355
H	-0.84385911474329	1.86512041161900	2.84362820195361

Table S14. Atomic coordinates for optimized structure of **Np 3^B^**.

PBE0:

final single point energy: -33107.046901580929 a.u.

final Gibbs free energy: -33106.66556610 a.u.

Np	-0.09185054692019	-0.03732028726643	0.03255764968124
N	-1.49147733322562	0.31893239109737	-1.98871585961421
N	-2.37721832493523	0.72028697368206	0.92344453370232
N	1.15974029101567	1.38889033778493	-1.66237805267522
N	1.27555768883541	-2.00690567993623	0.73302574292477
S	0.10979892511334	-1.71791481279536	-2.17250408058082
S	-2.18899182227735	-1.84671754045383	0.53457661990591
S	0.03723612268000	2.77517856807861	0.22259215574319
S	2.60050155673189	0.22955591822555	0.89348696863069
O	-0.15855722684735	-0.16194438873997	2.54498599249695
C	-1.08610071839730	-0.64582999937339	-2.83179578776984
C	-1.61542036860999	-0.72996123186759	-4.12142907779224
C	-2.55998784881174	0.19585611484755	-4.51337830924653
C	-2.96770386766681	1.19095666117018	-3.62728509773937
C	-2.40236146472963	1.21418230196588	-2.37080431618697
C	-3.03181216788132	-0.44108738930761	1.09533076120039
C	-4.30375327015603	-0.47020116519278	1.67622655948016
C	-4.88685462396695	0.71537994875667	2.06876295891301
C	-4.20239282479745	1.91317938943097	1.88030022498531
C	-2.95036768750323	1.86329532924894	1.30491507906008
C	1.00790565134106	2.64078983582274	-1.20778179379205
C	1.59809052604707	3.72271398209975	-1.86674355652031
C	2.34757029199970	3.48224452996531	-2.99862498253345
C	2.50187114596556	2.17642899600233	-3.45791625401735
C	1.88981796767078	1.16079761569832	-2.75481387442095
C	2.44937773628793	-1.48100652838562	1.12627336227717
C	3.44110120043981	-2.30259158372907	1.67441031394952
C	3.19904552056884	-3.65410297775487	1.79226278263431
C	1.98023298132664	-4.18283952974033	1.36913995870655
C	1.04681495288373	-3.31631257133721	0.84523091503326
C	-0.22130461634578	-1.30717692890411	3.41760166559567
C	-0.61800629636904	-0.74026885587076	4.75882894713289
C	0.13873380579665	0.58087006649983	4.76505129789845
C	-0.00482191502676	1.04348238806973	3.32884659186898
H	3.08522442988249	1.95177852685772	-4.34035982925056
H	-2.67011572724739	1.96511147267443	-1.63750827606970

H	1.96878089355625	0.12430093371084	-3.05961794851697
H	-4.62919660336192	2.86330483132671	2.17089493809868
H	-1.27651760841087	-1.51246955543255	-4.78709332083862
H	4.37778022150809	-1.86414249357799	1.99185285722148
H	-2.37693041589128	2.76504317759349	1.12643689936082
H	3.95768779579966	-4.30397145454258	2.21280443176990
H	-3.70484830050755	1.93086555419419	-3.90719628953500
H	1.76294144984890	-5.23922316712274	1.44473172954568
H	-4.80607224986574	-1.41984853310141	1.80399771437032
H	0.07920404565377	-3.66182458154523	0.50127701145265
H	1.45831220362500	4.72323808789762	-1.47960923137279
H	0.76915542442997	-1.76961370904209	3.45686292414618
H	-0.93386846185047	-2.00609694467356	2.98487851680925
H	-2.98186941500666	0.14861121019944	-5.51049009968194
H	-1.69634337835862	-0.56814610001238	4.79359598728486
H	-0.34223206226414	-1.40019196349400	5.58071798435653
H	2.81439886294852	4.30669533901695	-3.52484513337955
H	1.19015470038840	0.41232153780557	5.00688111764507
H	-0.26448285676614	1.31021523193381	5.46657613369046
H	-5.87278012408250	0.71232628297929	2.51888237019353
H	-0.90155861297036	1.64928456279487	3.18324385865024
H	0.86236114570632	1.58251877276800	2.94938084011796

Table S15. Atomic coordinates for optimized structure of **Np 3**^{C}.

PBE0:

final single point energy: -33107.048270131432 a.u.

final Gibbs free energy: -33106.66686959 a.u.

Np	-0.09139237003838	0.01747084984789	0.06638361650050
N	-1.06087019203986	-1.26006447093648	-1.85310236087054
N	-1.28515280701279	-2.04738503428342	0.99280814259685
N	0.89275685029503	1.10812749227739	-1.95058010699497
N	1.06756298230018	2.08496200615588	0.94821409849708
S	1.25036037907550	-2.15625930056766	-1.12762063472477
S	-2.77263117119942	0.08522539292815	0.90686252282818
S	-1.40999109468638	2.07775190269937	-1.27054362492339
S	2.67685518270789	0.06657318442995	0.73659657647682
O	0.04702438290471	-0.04240046197272	2.56451442307119
C	-0.18359136905458	-2.25076714466120	-2.08908934444603
C	-0.46027122822254	-3.23525628453745	-3.04344972976377
H	0.25768424423517	-4.02555371055084	-3.21823377022276
C	-1.64762350259750	-3.16856698975877	-3.74020776203901
H	-1.88013292488003	-3.92097199675229	-4.48501584214619
C	-2.54658148744381	-2.13339544862695	-3.48486507482483
H	-3.48499455161661	-2.05501620271301	-4.01648337627740
C	-2.21050941955837	-1.20027030248310	-2.52876810263934
H	-2.86014064810761	-0.37097034905683	-2.27781775500833
C	-2.49296160484285	-1.57119983325421	1.33927065413879
C	-3.42734502116478	-2.38944317358180	1.98129144551817
H	-4.39300735264587	-1.98343544527354	2.25185766740705
C	-3.09246258613090	-3.69967258907165	2.25045184792800
H	-3.80362650178728	-4.34905344143935	2.74772803586661
C	-1.83957691954767	-4.18242620978431	1.87987622240765
H	-1.54550664964465	-5.20502395398832	2.07294281098118
C	-0.96927224573177	-3.31705544623720	1.25114461989509
H	0.01743744233982	-3.62923913459328	0.92930035032832
C	0.02279217349911	2.08661705485041	-2.24974878926520
C	0.29438448284945	2.99479810783440	-3.27583509617629
H	-0.41718329947470	3.77888334712260	-3.49797193701125
C	1.47007292391256	2.86032916583511	-3.98481763597162
H	1.69872426350886	3.55192519432384	-4.78748990374502
C	2.35950030547613	1.83598169027847	-3.66726584416118
H	3.28788841498990	1.70526541434356	-4.20613292363294
C	2.02993998897309	0.98274495225077	-2.63586771285846
H	2.67405763561829	0.16865970547108	-2.32888174719961

C	2.31974601409589	1.68203720740771	1.23262970799876
C	3.21636981590154	2.54807692780453	1.87036880239748
H	4.21895574292266	2.20666472350777	2.09147380891782
C	2.79820075469068	3.81943779046650	2.19747430652127
H	3.48013464570853	4.50329183115065	2.68962939227610
C	1.49816129545545	4.22252251135558	1.89348337306858
H	1.14078977792687	5.21429467558575	2.13456949489343
C	0.66834326409960	3.31843936185963	1.26819213233271
H	-0.35087236991020	3.56825063416023	0.99817777823849
C	-0.55502670481007	0.85842861549059	3.50010331746761
H	-1.27582407480926	1.46626902539016	2.95869436104935
H	0.23181798521762	1.49784210575190	3.91256599287880
C	-1.17186314051079	-0.04584516835912	4.55796915845093
H	-1.10381830861065	0.40566569976388	5.54706175311519
H	-2.22392777359956	-0.22311397635837	4.33666104151500
C	-0.36568310292644	-1.35559201657885	4.44753541435157
H	0.12296771817086	-1.62832687608059	5.38211688292477
H	-1.01275388682697	-2.18111032773381	4.15103234324892
C	0.65558979443702	-1.06443546044242	3.35909339981852
H	1.59064565396533	-0.66891002973329	3.76618407587636
H	0.88499164115455	-1.90659091793295	2.71077563411971

Table S16. Atomic coordinates for optimized structure of **Pu 4^B^**.

PBE0:

final single point energy: -33996.690729328613 a.u.

final Gibbs free energy: -33996.31000835 a.u.

Pu	-0.11208604667352	-0.02097632807817	-0.01110561873651
N	-1.40358325210881	0.30245834152917	-2.09284489317398
N	-2.39385673609779	0.72180123036995	0.91800078348368
N	1.14240849541453	1.43669618658433	-1.62653457134179
N	1.19512374362286	-2.00345390880654	0.73332343806367
S	0.15613312327490	-1.76313825133357	-2.16532350887354
S	-2.20828652188902	-1.81517182762528	0.40588008866559
S	0.01988321375403	2.77366650334990	0.29326613452172
S	2.58816225444026	0.18782459209435	0.79737789707119
O	-0.11325834954578	-0.10153631142043	2.50822598419153
C	-1.01007832295380	-0.70461109015289	-2.89011783866309
C	-1.53788571167221	-0.83921454438119	-4.17624794848094
C	-2.46267374492330	0.08608184527459	-4.61362585507885
C	-2.85538113768613	1.12774264647539	-3.77504377354224
C	-2.29709003904462	1.19620449651214	-2.51694840482470
C	-3.02168823234899	-0.45116790848662	1.10128279919130
C	-4.22783952285460	-0.52216956660130	1.80339285142858
C	-4.78160500289659	0.64144120152448	2.29507728026057
C	-4.13223542289543	1.85419654684386	2.08517406864271
C	-2.93845133416101	1.84209703672803	1.39395396881437
C	0.97920118211371	2.67780692226758	-1.14943417168904
C	1.54793352940304	3.77667493318285	-1.79820573575737
C	2.28409960427976	3.56263713873903	-2.94467511073261
C	2.44743930744797	2.26672745781834	-3.42746924859992
C	1.85875066175431	1.23214090909774	-2.73158849756981
C	2.39576315691171	-1.50789601872828	1.07873130083554
C	3.38190876282088	-2.34721795243039	1.61094060932729
C	3.10542728236397	-3.68809758755231	1.76329017043560
C	1.85891676305460	-4.18755048338463	1.38730109382277
C	0.93476500473256	-3.30401202047591	0.87547018383276
C	-0.26256905245783	-1.24532428017354	3.36941986935851
C	-0.70548555650057	-0.66561568906595	4.69042673127980
C	0.11869685991038	0.61330105259310	4.74779112778640
C	0.09215819888421	1.08495035935289	3.30624955621704
H	3.02107393760824	2.06296663460511	-4.32124873855874
H	-2.55654827117642	1.98154657322402	-1.81680878553655

H	1.94879216343267	0.20107141062751	-3.05187980335155
H	-4.54088479298388	2.78750809296482	2.44758470309821
H	-1.21434770199793	-1.65912537733622	-4.80340748187603
H	4.34065845936736	-1.92983154360659	1.88862962933778
H	-2.39049263370511	2.75640644868059	1.19977318058368
H	3.85784935729260	-4.35197533227003	2.17298697875105
H	-3.57670889417869	1.86807413897422	-4.09255878030143
H	1.61319621265091	-5.23551580853489	1.48921282569117
H	-4.70524069931027	-1.48254941734728	1.94526097588806
H	-0.05203095974001	-3.62831586615608	0.56824599397046
H	1.40176562879046	4.76922787661813	-1.39347910118106
H	0.70484586595056	-1.74841722416419	3.45817165028177
H	-0.97900585291934	-1.91605780521051	2.89944758442091
H	-2.88213371735664	0.00166343998809	-5.60928202692986
H	-1.77375980198441	-0.43773043273565	4.66346646066267
H	-0.51197685249438	-1.34202677752964	5.52238752404162
H	2.73410731968051	4.40075898215651	-3.46383499287911
H	1.14175584689026	0.38777878085009	5.05580382723591
H	-0.28742556182321	1.36307984851772	5.42573695148731
H	-5.71866493694500	0.60842109464792	2.83868907979096
H	-0.74239881189404	1.76182121149209	3.11315793840770
H	1.01845633637210	1.55485231690351	2.97850887179871

Table S17. Atomic coordinates for optimized structure of **Pu 4**^{C}.

PBE0:

final single point energy: -33996.693237192907 a.u.

final Gibbs free energy: -33996.31156563 a.u.

Pu	-0.05157773488183	-0.03591359806106	0.13220906125880
N	-1.05181117049025	-1.20068406604011	-1.82576084990023
N	-1.23015587756161	-2.12807941028226	0.99029794031147
N	0.87569403860288	1.05956770694100	-1.90122726740413
N	1.11086486570483	2.07887451067441	0.89992588288109
S	1.25239733966906	-2.15882800639377	-1.14726484904902
S	-2.76627395240010	-0.03788419079913	0.86758572180299
S	-1.40510990236320	2.03675007698565	-1.17884883054453
S	2.68964002045784	0.02423547274058	0.73958343261828
O	0.03197659624087	-0.00802849604493	2.59805975557552
C	-0.19480028779019	-2.19821493511115	-2.09659104082865
C	-0.49287789615475	-3.15006696686963	-3.07648962544537
H	0.21040355966115	-3.94732121907264	-3.27737275814525
C	-1.68186693265493	-3.04194306327085	-3.76611736424804
H	-1.93131238514548	-3.76784902855975	-4.53142349884681
C	-2.55915559207851	-1.99874014001644	-3.47615476534601
H	-3.49879709648888	-1.88731619751024	-3.99970492665363
C	-2.20197570301918	-1.10073601065225	-2.49299239468431
H	-2.83488910394053	-0.26867702045583	-2.21249236137915
C	-2.45523568802648	-1.68095018966198	1.31666145562766
C	-3.37808100566819	-2.51959382772130	1.95044273557640
H	-4.35823488354500	-2.13737191177924	2.20300645368609
C	-3.01339303878200	-3.81833801477808	2.23306067472042
H	-3.71490856728443	-4.48327921989693	2.72338132044982
C	-1.74262293903731	-4.27125604478759	1.88380651678862
H	-1.42630497097146	-5.28493962789043	2.08811267980688
C	-0.88609788652282	-3.38846113420987	1.26135445896793
H	0.11197446674479	-3.67822812443126	0.95425306805846
C	0.00689818368832	2.04467748762444	-2.17933348287066
C	0.26638276404897	2.95858292975574	-3.20538475145370
H	-0.44552144428790	3.74715865355038	-3.41017570888449
C	1.42878320489067	2.82438268600338	-3.93435470057770
H	1.64817292592354	3.52034887151737	-4.73582974432142
C	2.31797187792356	1.79309470473110	-3.63758415745442
H	3.23729916072840	1.66150109950543	-4.19161631365006
C	2.00014812473815	0.93436999644219	-2.60706436895879
H	2.64537646207323	0.11491113608152	-2.31797237760749
C	2.35285873752750	1.65964473521795	1.19898448492043

C	3.26642796744054	2.51801027662900	1.81964326157771
H	4.26106332321593	2.16000510834751	2.05018470176751
C	2.87416123986651	3.80551199855564	2.11708058200987
H	3.56887164437323	4.48590411910353	2.59583028951683
C	1.58454203376173	4.22721689112903	1.79929693340500
H	1.24660943855601	5.23086985460285	2.01815449623523
C	0.73733740250560	3.32620507073242	1.19072257037063
H	-0.27538223151906	3.59016353635515	0.91018840371505
C	-0.63110533751962	0.90134510814643	3.48526224395414
H	-1.33139399910415	1.48733743078831	2.89489157746310
H	0.12589607069887	1.55880169618024	3.92350287450702
C	-1.28600830267862	0.00549535009342	4.52611619555141
H	-1.28582079689035	0.47908765739326	5.50698667500826
H	-2.31879054289126	-0.20150847500487	4.24739218217765
C	-0.44588628045505	-1.28711761014081	4.49074391492861
H	0.00539636250285	-1.52098825108340	5.45410449022405
H	-1.05979933938931	-2.13591042058510	4.19007415751577
C	0.61947294332015	-1.00480505697468	3.44263390817327
H	1.52946992862300	-0.58458259055786	3.87996667572845
H	0.88826165305427	-1.85725644418454	2.82350049237341

Table S18. Atomic coordinates for optimized structure of **[U(PyS)$_5$]$^-$**.

PBE0:

final single point energy: -32652.066629339326 a.u.

final Gibbs free energy: -32651.72377438 a.u.

U	0.03562738439169	-0.11064270711861	0.05409541831467
N	0.10124374785846	-1.91635859591252	-1.82028708272992
N	-1.76421995612522	1.17669659798144	-1.32696846928497
N	2.03242769005321	-1.72320899533219	0.22757228252576
N	2.35783706932627	1.13258807323449	0.35202278115512
N	-2.36576989645655	-1.28363171906472	-0.01648422063565
S	1.05095757932979	0.37427284867992	-2.60785242889803
S	-0.12142050178622	2.75826810831982	-0.09219874199283
S	-0.03718149713683	-2.62885671945328	1.50881290060985
S	1.22395148783689	0.47291675894477	2.57983944100943
S	-2.05280986927023	0.43163471412345	1.90511204643393
C	0.65290655674392	-1.28514952439325	-2.87028781889149
C	0.91108949025539	-1.97584508158721	-4.06328048695279
H	1.34088102469717	-1.44196709172645	-4.90041804292346
C	0.63293922944598	-3.32195014191145	-4.13261200250585
H	0.83544778211442	-3.87136083779370	-5.04492257487769
C	0.10448007941147	-3.97424394826811	-3.01954232261843
H	-0.10913125894546	-5.03427933051733	-3.02897786149318
C	-0.14435641486468	-3.22589876261881	-1.88996579110836
H	-0.54117204621392	-3.67292682794104	-0.98593805448328
C	-1.49486995473756	2.47244912957122	-1.09056657721890
C	-2.28175806213157	3.48234495495927	-1.66224080175301
H	-2.05379818008698	4.51686742285457	-1.44186457529559
C	-3.31811959395236	3.13489417301848	-2.49836260677886
H	-3.93238919495348	3.90449538712136	-2.95167912604563
C	-3.56604714716625	1.78830133369763	-2.76064431005932
H	-4.36277694322816	1.47392950020934	-3.42095853087682
C	-2.76312319015009	0.85191910079219	-2.14679897411272
H	-2.91784904427435	-0.20528151964743	-2.31705625608402
C	1.59102199416857	-2.75942121545317	0.96320189674525
C	2.42829382194014	-3.85569359724651	1.21506921127619
H	2.05901571847497	-4.67413727778942	1.81882527622591
C	3.69681275022285	-3.87010211363346	0.68071958817315
H	4.35279769086380	-4.71342965047537	0.86429842200097
C	4.12909556041527	-2.79880798674179	-0.10027435016604
H	5.11499883494412	-2.77908694757695	-0.54413496158639
C	3.25803006682701	-1.74907157888813	-0.29416672196818

H	3.53624231796573	-0.89146663494127	-0.89348144782772
C	2.51504228137661	1.18453842933672	1.68345715993983
C	3.64378664214196	1.78955428361188	2.25269080174589
H	3.74737047925178	1.81101212497394	3.32959666994167
C	4.59124979761867	2.35035748365571	1.42513497228033
H	5.47025358963476	2.82370282669894	1.84776564964880
C	4.40668650807676	2.30900278542123	0.04500655224491
H	5.12320944174257	2.74431964891828	-0.63835579059706
C	3.27255389077043	1.68968628976131	-0.43860157839107
H	3.07792228329919	1.62090092694900	-1.50264706361520
C	-2.99029124053014	-0.72280822435801	1.03240176787059
C	-4.30696620915134	-1.07529915190369	1.36183788399605
H	-4.78031603405658	-0.60674696534416	2.21467568631013
C	-4.96681529861486	-2.00711548863836	0.59315322110384
H	-5.98514112988551	-2.28988863777401	0.83433758097701
C	-4.31400741570059	-2.57678478215907	-0.49753478606336
H	-4.79619538679391	-3.30645299497984	-1.13380653728811
C	-3.01900468475826	-2.17846061866635	-0.75595027038784
H	-2.48113564022879	-2.58483823297923	-1.60274004501747

Table S19. Results of quantum chemical calculations. Average bond lengths (d) in the geometry optimization structures (isomer B). Ratio $|V(\boldsymbol{r}_b)|/G(\boldsymbol{r}_b)$ (where $V(\boldsymbol{r}_b)$: potential energy density and $G(\boldsymbol{r}_b)$: Lagrangian kinetic energy) and delocalization indices (DI) obtained from QTAIM analysis. Natural charges obtained from NBO/NLMO analysis. Bond energies (E), their ionic (E_{ion}) and covalent (E_{cov}) contributions, and percentage covalent bond contributions obtained from IQA analysis.

An	Th	Pa	U	Np	Pu
$d_{An–N}$ (Å)	2.59(2)	2.57(2)	2.54(2)	2.52(3)	2.51(4)
$d_{An–S}$) (Å)	2.89(1)	2.85(1)	2.83(1)	2.81(2)	2.80(2)
$d_{An–O}$ (Å)	2.56	2.52	2.51	2.52	2.52
$\|V(\boldsymbol{r}_b)\|/G(\boldsymbol{r}_b)_{An–N}$	1.210	1.194	1.182	1.177	1.166
$\|V(\boldsymbol{r}_b)\|/G(\boldsymbol{r}_b)_{An–S}$	1.365	1.368	1.363	1.343	1.325
$\|V(\boldsymbol{r}_b)\|/G(\boldsymbol{r}_b)_{An–O}$	1.085	1.083	1.068	1.056	1.038
$DI_{An–N}$	0.306(9)	0.325(14)	0.335(15)	0.343(22)	0.346(29)
$DI_{An–S}$	0.421(6)	0.457(12)	0.475(17)	0.489(27)	0.508(22)
$DI_{An–O}$	0.255	0.272	0.275	0.260	0.250
natural charge An (*e*)	0.86	0.68	0.57	0.53	0.51
natural charge N (*e*)	−0.51(1)	−0.49(1)	−0.49(1)	−0.48(1)	−0.48(1)
natural charge S (*e*)	−0.16(1)	−0.13(2)	−0.11(2)	−0.11(2)	−0.10(2)
natural charge O (*e*)	−0.50	−0.49	−0.47	−0.48	−0.48
$E_{An–N}$ (kcal·mol^{-1})	−486.8(48)	−474.5(74)	−463.0(77)	−454.5(110)	−442.0(111)
$E_{An–S}$ (kcal·mol^{-1})	−187.0(42)	−182.3(66)	−175.3(49)	−169.3(59)	−161.7(72)
$E_{An–O}$ (kcal·mol^{-1})	−411.7	−405.3	−392.6	−379.2	−364.2
$E_{An–N,ion}$ (kcal·mol^{-1})	−447.3(37)	−432.4(57)	−419.5(59)	−409.8(80)	−397.0(75)
$E_{An–S,ion}$ (kcal·mol^{-1})	−141.0(41)	−132.1(65)	−123.2(50)	−116.2(47)	−107.6(67)
$E_{An–O,ion}$ (kcal·mol^{-1})	−378.6	−369.8	−356.8	−345.4	−331.7
$E_{An–N,cov}$ (kcal·mol^{-1})	−39.5(12)	−42.1(19)	−43.5(20)	−44.7(30)	−45.0(38)
$E_{An–S,cov}$ (kcal·mol^{-1})	−46.1(6)	−50.1(12)	−52.1(17)	−53.2(23)	−54.1(19)
$E_{An–O,cov}$ (kcal·mol^{-1})	−33.1	−35.4	−35.8	−33.8	−32.5
$cov_{An–N}$ (%)	8.1(1)	8.9(1)	9.4(1)	9.8(1)	10.2(1)
$cov_{An–S}$ (%)	24.6(5)	27.5(11)	29.7(12)	31.4(11)	33.5(14)
$cov_{An–O}$ (%)	8.0	8.7	9.1	8.9	8.9

13. Infrared spectroscopy

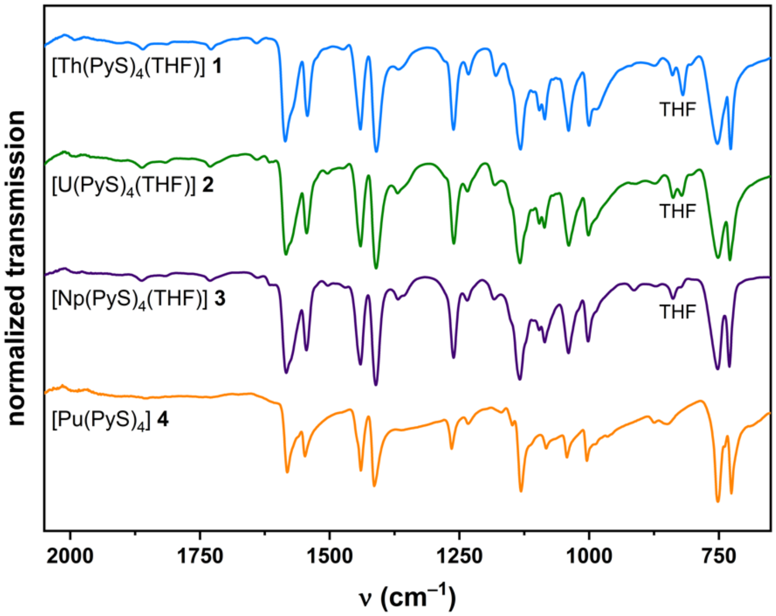

Figure S41. ATR-IR transmission spectra of the An complexes **1** – **4**. Significant vibrational bands of coordinating THF molecules (δ(C–H): 820 cm^{-1}, 840 cm^{-1}) are marked.[6,7]

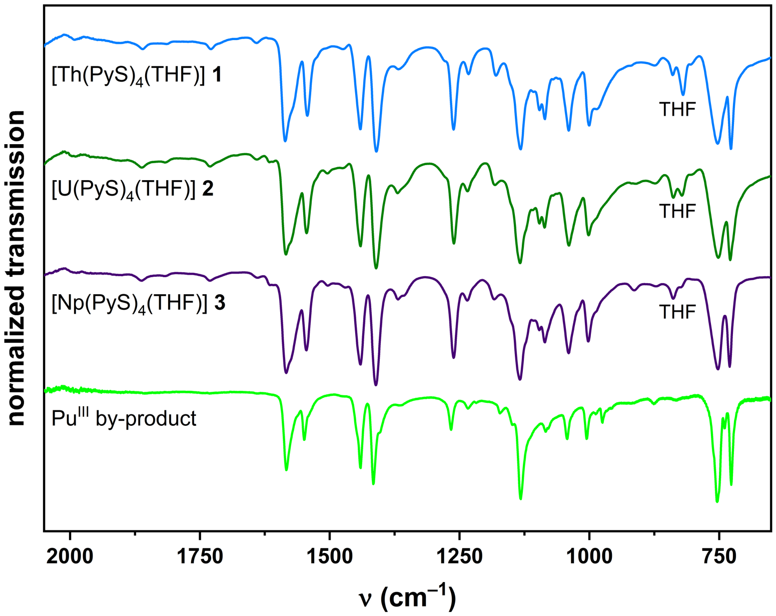

Figure S42. ATR-IR transmission spectra of the An complexes **1** – **3**. and the green trivalent by-product of the complex syntheses with Pu and PyS^{-}. Significant vibrational bands of coordinating THF molecules (δ(C–H): 820 cm^{-1}, 840 cm^{-1}) are marked.[6,7]

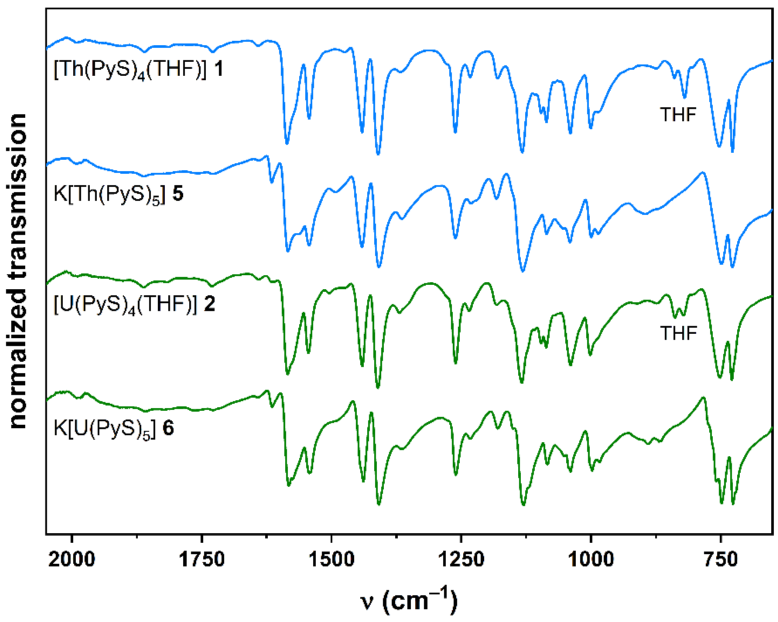

Figure S43. Comparison of different complex compositions: ATR-IR transmission spectra of the An complexes **1**, **2**, **5** and **6**. Significant vibrational bands of coordinating THF molecules (δ(C–H): 820 cm^{-1}, 840 cm^{-1}) are marked.[6,7]

14. Superconducting quantum interference device (SQUID) magnetization measurements

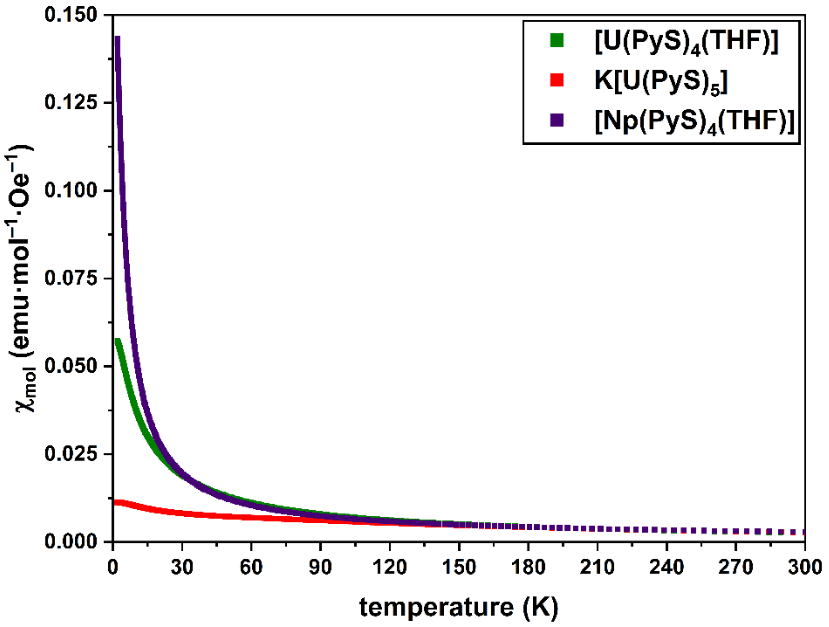

Figure S44. Molar magnetic susceptibilities χ_{mol} (emu·mol^{-1}·Oe^{-1}) vs T (K) in a range of 1.9 – 300 K of [U(PyS)$_4$(THF)] **2**, [Np(PyS)$_4$(THF)] **3** and K[U(PyS)$_5$] **6**. Selected data points see **Table 4**.

For the SQUID magnetization measurements and the calculation of magnetic parameters, CGS-emu and SI units as well as their conversions known from literature are used.[8] The temperature (K) and the magnetic moment (emu) were obtained from the measurements, where the *Squidlab* program (version: 2.9.1) was used for background correction and data adjustment using the *Levenberg-Marquardt* method.[9] By averaging 32 measurement points, the amount of data was reduced and the molar magnetic susceptibility χ_{mol} (cm^3/mol) and the effective magnetic moment μ_{eff} (μ_B) were calculated.

$$\chi_{mol}\left(\frac{cm^3}{mol}\right) = \frac{\mu(emu)}{H(Oe)\cdot\left(\frac{m(g)}{M(g\cdot mol^{-1})}\right)} \qquad (1)^{10}$$

$$\mu_{eff}(\mu_B) = \sqrt{\frac{3k_B}{N_A\mu_B^2}}\cdot\sqrt{\chi_{mol}\cdot T} = \sqrt{8\cdot\chi_{mol}\left(\frac{cm^3}{mol}\right)\cdot T(K)} \qquad (2)^{8,10}$$

To calculate the magnetization per formula unit, the magnetic field (Oe) and the magnetic moment (emu) were obtained from the measurements. By averaging two measurement points, the amount of data was reduced. The magnetic field μ_0H (T) was expressed in SI units.

$$M_{f.u.}(\mu_B) = \frac{\mu(emu)\cdot M(g\cdot mol^{-1})}{m(g)\cdot N_A\cdot\mu_B} \qquad (3)^{10,11}$$

15. ASAP-APCI mass spectrometry

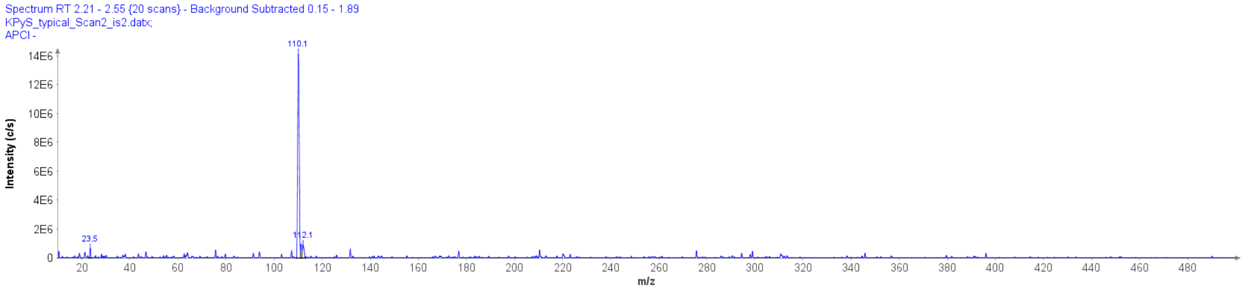

Figure S45. ASAP-APCI mass spectrum of KPyS in negative measurement mode with method-dependent fragments (*m/z* 110: PyS^{-}).

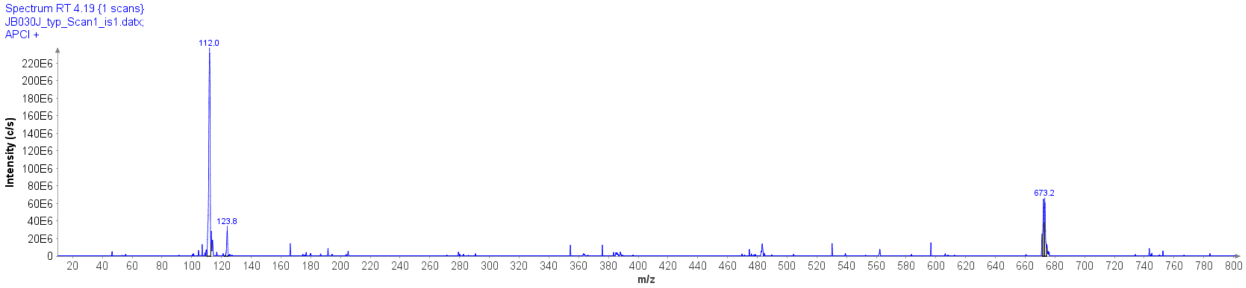

Figure S46. ASAP-APCI mass spectrum of **1** in positive measurement mode with method-dependent fragments (*m/z* 112: PySH + H^{+}, *m/z* 673: $^{232}Th(PyS)_4$ + H^{+}).

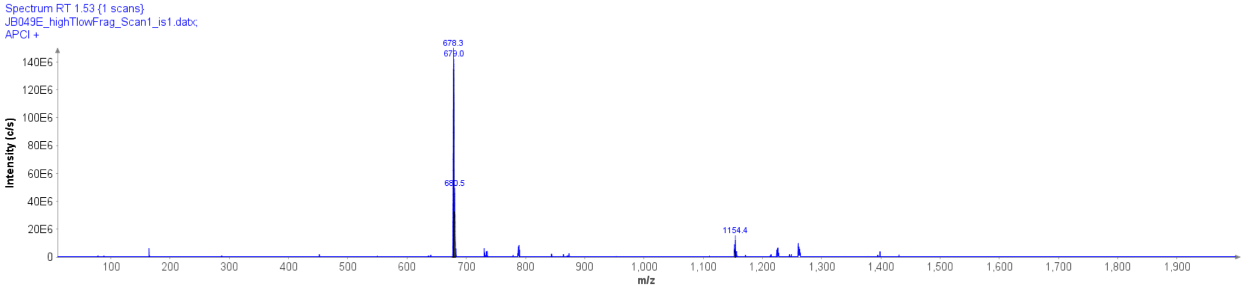

Figure S47. ASAP-APCI mass spectrum of **2** in positive measurement mode with method-dependent fragments (*m/z* 679: $^{nat}U(PyS)_4$ + H^{+}).

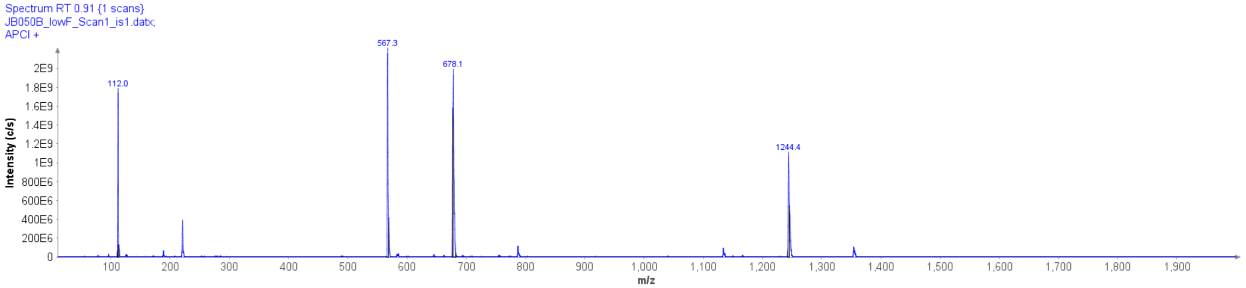

Figure S48. ASAP-APCI mass spectrum of **3** in positive measurement mode with method-dependent fragments (*m/z* 567: $^{237}Np(PyS)_3^{+}$, *m/z* 678: $^{237}Np(PyS)_4$ + H^{+}, *m/z* 1244: $(^{237}Np(PyS)_3)_2(PyS)^{+}$).

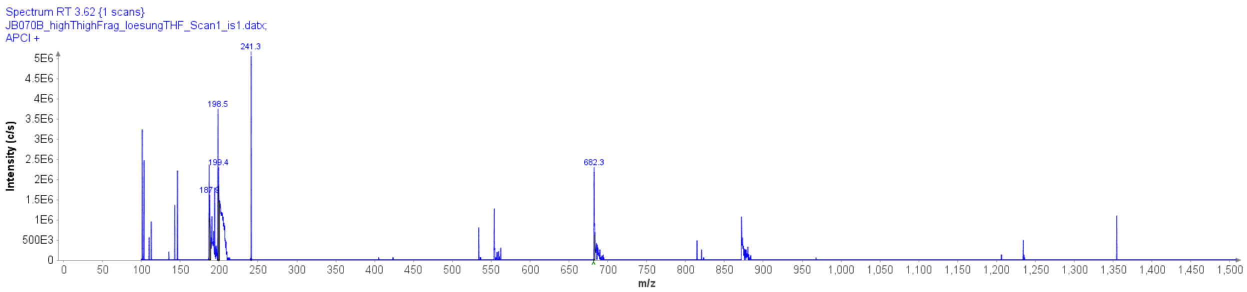

Figure S49. ASAP-APCI mass spectrum of **4** in positive measurement mode with method-dependent fragments (*m/z* 682: $^{242}Pu(PyS)_4 + H^+$).

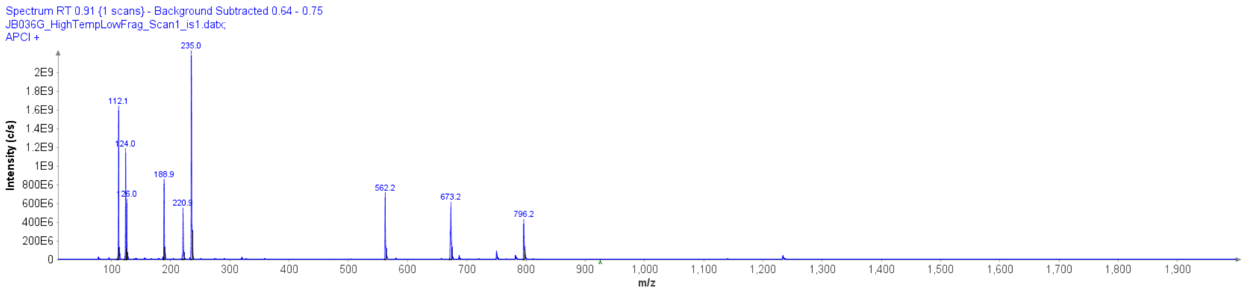

Figure S50. ASAP-APCI mass spectrum of **5** in positive measurement mode with method-dependent fragments (*m/z* 112: PySH + H^+, *m/z* 562 $^{232}Th(PyS)_3{}^+$, *m/z* 673: $^{232}Th(PyS)_4 + H^+$, *m/z* 796: $K^{232}Th(PyS)_4(C_3H_2NS) + H^+$).

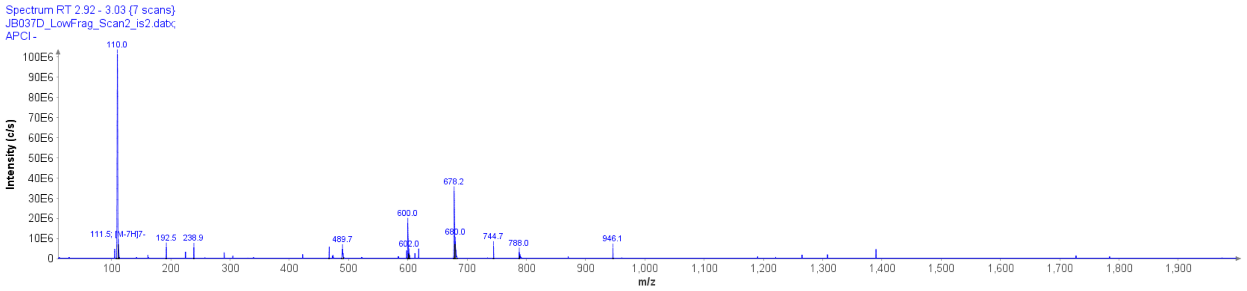

Figure S51. ASAP-APCI mass spectrum of **6** in negative measurement mode with method-dependent fragments (*m/z* 110: PyS^-, *m/z* 678: $^{nat}U(PyS)_4 + e^-$, *m/z* 788: $^{nat}U(PyS)_5{}^-$).

REFERENCES

(1) *The Chemistry of the Actinide and Transactinide Elements*; Morss, L. R., Edelstein, N. M., Fuger, J., Eds.; Springer Netherlands: Dordrecht, 2006. https://doi.org/10.1007/1-4020-3598-5.

(2) de Castro, V. D.; de Lima, G. M.; Filgueiras, C. A. L.; Gambardella, M. T. P. The Molecular Structure and Spectral Studies of Mercaptopyridyl-Based Ligands. *J. Mol. Struct.* **2002**, *609* (1–3), 199–203. https://doi.org/10.1016/S0022-2860(01)00976-0.

(3) Fulmer, G. R.; Miller, A. J. M.; Sherden, N. H.; Gottlieb, H. E.; Nudelman, A.; Stoltz, B. M.; Bercaw, J. E.; Goldberg, K. I. NMR Chemical Shifts of Trace Impurities: Common Laboratory Solvents, Organics, and Gases in Deuterated Solvents Relevant to the Organometallic Chemist. *Organometallics* **2010**, *29* (9), 2176–2179. https://doi.org/10.1021/om100106e.

(4) Shannon, R. D. Revised Effective Ionic Radii and Systematic Studies of Interatomic Distances in Halides and Chalcogenides. *Acta Crystallogr. Sect. A* **1976**, *32* (5), 751–767. https://doi.org/10.1107/S0567739476001551.

(5) Baker, R. J.; Hashem, E.; Motevalli, M.; Ogilvie, H. V.; Walshe, A. The Coupling of Pyridine and Dichloromethane Mediated by UO_2Cl_2. *Zeitschrift für Anorg. und Allg. Chemie* **2010**, *636* (3–4), 443–445. https://doi.org/10.1002/zaac.200900342.

(6) Socrates, G. *Infrared and Raman Characteristic Group Frequencies*, 1st ed.; Wiley: Weinheim, 2001.

(7) Steger, E. *Strukturanalytik*, 1st ed.; Verlag für Grundstoffindustrie: Leipzig, 1992.

(8) Lueken, H. Praktische Anleitung zur Messung und Interpretation magnetischer Eigenschaften. *Angew. Chemie* **2006**, *118* (47), 8233–8240. https://doi.org/10.1002/ange.200603275.

(9) Coak, M. J.; Liu, C.; Jarvis, D. M.; Park, S.; Cliffe, M. J.; Goddard, P. A. SquidLab — A User-Friendly Program for Background Subtraction and Fitting of Magnetization Data. *Rev. Sci. Instrum.* **2020**, *91* (2). https://doi.org/10.1063/1.5137820.

(10) Lueken, H. *Magnetochemie - Eine Einführung in Theorie Und Anwendung*, 1st ed.; B.G. Teubner Verlagsgesellschaft: Stuttgart, Leipzig, 1999.

(11) Grödler, D.; Weidemann, M. L.; Lichtenberg, A.; Greven, T.; Nickstadt, R.; Haydo, M.; Wickleder, M.; Klein, A.; Johrendt, D.; Mathur, S.; Zegke, M.; Raauf, A. Heterobimetallic Uranyl(VI) Alkoxides of Lanthanoids: Formation through Simple Ligand Exchange. *Chem. Commun.* **2022**, *58* (6), 835–838. https://doi.org/10.1039/D1CC05444A.
